# Proteomics Analysis of Dorsal Striatum Reveals Changes in Synaptosomal Proteins following Methamphetamine Self-Administration in Rats

**DOI:** 10.1371/journal.pone.0139829

**Published:** 2015-10-20

**Authors:** Peter J. Bosch, Lifeng Peng, Bronwyn M. Kivell

**Affiliations:** Centre for Biodiscovery, School of Biological Sciences, Victoria University of Wellington, Wellington, New Zealand; University of Leicester, UNITED KINGDOM

## Abstract

Methamphetamine is a widely abused, highly addictive drug. Regulation of synaptic proteins within the brain’s reward pathway modulates addiction behaviours, the progression of drug addiction and long-term changes in brain structure and function that result from drug use. Therefore, using large scale proteomics studies we aim to identify global protein expression changes within the dorsal striatum, a key brain region involved in the modulation of addiction. We performed LC-MS/MS analyses on rat striatal synaptosomes following 30 days of methamphetamine self-administration (2 hours/day) and 14 days abstinence. We identified a total of 84 differentially-expressed proteins with known roles in neuroprotection, neuroplasticity, cell cytoskeleton, energy regulation and synaptic vesicles. We identify significant expression changes in stress-induced phosphoprotein and tubulin polymerisation-promoting protein, which have not previously been associated with addiction. In addition, we confirm the role of amphiphysin and phosphatidylethanolamine binding protein in addiction. This approach has provided new insight into the effects of methamphetamine self-administration on synaptic protein expression in a key brain region associated with addiction, showing a large set of differentially-expressed proteins that persist into abstinence. The mass spectrometry proteomics data are available via ProteomeXchange with identifier PXD001443.

## Introduction

Methamphetamine is an addictive psychostimulant drug of abuse, with an estimated global annual prevalence of 0.7% and reports of increasing use [1], heightening the need for better understanding of long-term changes in the brain following repeated use. Methamphetamine causes extensive protein expression changes in the rodent and monkey brain, notably to dopaminergic markers of the mesolimbic system [2,3].

Many studies use experimenter-administered methamphetamine “binge” treatments that deliver between 10–40 mg/kg to experimental animals in a single day [4,5]. These studies consistently report reduced dopamine, serotonin, dopamine transporter, vesicular monoamine transporter binding and increased glial fibrillary acidic protein following binge regimens in rats, mice and monkeys [3]. Many of these changes occur in the striatum and can persist longer than 6 months [5].

In human chronic users, methamphetamine administration occurs in either consistent low-doses or high-dose binge cycles [6]. Methamphetamine is readily self-administered by rodents and is a method with greater face validity to experimenter-administered models [7]. Global protein expression changes are poorly understood as previous methamphetamine self-administration studies have focused on dopaminergic markers [7,8], with transient or reduced effects seen compared to binge regimens.

Proteomics has been used to study the effects of multiple drugs of abuse in animal models, producing a valuable resource to further study biochemical pathways and gene/protein networks [9]. Using proteomics techniques, changes in energy metabolism, oxidative stress, protein modification and degradation have been shown in the rat brain following methamphetamine administration [10,11]. Previous studies using neurotoxic doses of methamphetamine (i.e. >40 mg/kg/day) showed differential expression of proteins involved in oxidative stress, mitochondrial dysfunction, cell cytoskeleton and apoptosis [10,12,13]. Mass spectrometry-based proteomics has been applied to amphetamine [14], cocaine [15] and methamphetamine [16] self-administration, which identified a large number of proteins that persist into abstinence. A proteomics study of rat hippocampus during amphetamine self-administration, abstinence and relapse reported over-representation of cytoskeletal proteins during abstinence suggesting the utility of these techniques to identify proteins conferring individual vulnerability to relapse [14].

The synaptosome is a sub-cellular fraction containing the pre-synaptic terminal and post-synaptic density [17], enabling the study of synaptic processes in response to drug treatments [18]. Synaptic plasticity occurs following repeated exposure to all drugs of abuse [19]; therefore, synaptic protein regulation may provide insight regarding biochemical pathways altered following repeated drug administration.

Due to limited information on protein changes following methamphetamine self-administration, we used proteomics to identify and characterise persistent protein changes in synaptosomes following methamphetamine self-administration in rats following 14 days abstinence. Investigation of neurobiological changes during abstinence are an essential step towards developing new treatment strategies for drug addiction.

## Materials and Methods

All research was approved by the Animal Ethics Committee, Victoria University of Welligton, New Zealand (2012R34). Male Sprague-Dawley rats (Rattus norvegicus, 300–350 g) were housed individually in temperature (19–21°C) and humidity (55%) controlled hanging polycarbonate cages on a 12 hour light/dark cycle. At the start of the experiment, there were 8 control rats and 9 methamphetamine self-administration rats. Animals had ad libitum access to food and water except during self-administration. Animals were deeply anaesthetised with Ketamine (90 mg/kg, I.P.) and Xylazine (9 mg/kg, I.P.), fitted with chronic indwelling jugular catheters and assigned to control or methamphetamine self-administration groups. The study design isolated dorsal striatum (dStr) synaptosomes from control (n = 8) or methamphetamine self-administration (n = 7) rats. Proteins were subsequently extracted and analysed using LC-MS/MS, to compare between the two treatments. Synaptosomal proteins used for proteomics analysis were based on sufficient protein yield; therefore, n = 6 for each group was used.

### Methamphetamine self-administration

At the start of the experiment, there were 8 control rats and 9 methamphetamine self-administration rats. Rats were fitted with chronic indwelling jugular catheters and assigned to control or methamphetamine self-administration groups. Rats received training for methamphetamine in standard operant chambers 5 days post-surgery (Med Associates, ENV-001, St Albans, Vermont, USA) using previously reported methods [4]. Active lever depression lead to a 12 s, 0.1 mL infusion of methamphetamine-HCl (BDG Synthesis, Wellington, NZ, 0.1 mg/kg/infusion) dissolved in sterile heparinised (3 U/mL) physiological saline concurrent with light illumination above the active lever. Control animals received heparinised saline infusions upon depression of the active lever. Once on a fixed ratio-5 (FR-5) schedule, rats had daily 2 hour sessions for 6 days/week for 20 days followed by 14 days abstinence [20]. Responses were recorded using Med Associates software (MED-PC IV, version 4.2). Rats that completed the whole experiment (control, 8; methamphetamine self-administration, 7) were euthanised, brains rapidly removed and dStr rapidly dissected using an acrylic stereotaxic brain matrixes block (Alto, AgnTho’sAB, Sweden) and coordinates from Paxinos and Watson [21].

### Synaptosome purification

Synaptosome purification was performed using methods previously described [17]. Briefly, Percoll (pH 7.4, GE Healthcare, Auckland, NZ) gradients were mixed with 50 mM DTT (Merck Ltd, NZ) and gradient buffer (1.28 M sucrose, 20 mM Tris, 4 mM EDTA, pH 7.4) to make 23%, 10% and 3% solutions in 10.4 mL polycarbonate tubes (Cat# 355651, 10.4 mL, Beckman Coulter, Palo Alto, CA, USA).

The dStr from both sides of the rat brain were combined and homogenised in a glass-teflon homogeniser (10 strokes) (Wheaton Scientific, NJ, USA) in gradient buffer (9 mL/g). Homogenates were centrifuged at 1,000 x g for 10 min (4°C), the supernatant S1 was removed and diluted to 4–5 mg/mL and layered onto the 3% Percoll layer. Samples were centrifuged at 30,000 x g for 5 min at maximum speed (4°C) (Beckman Coulter Optima^TM^ L-100 XP Ultracentrifuge). The synaptosome fraction at the 23%/10% interface was removed, diluted in gradient buffer and centrifuged at 20,000 x g for 30 min (4°C). Synaptosome pellets were immediately removed and stored at -80°C until further use.

### Transmission electron microscopy (TEM)

TEM was used to directly visualise the synaptosome preparation using a standard TEM protocol [22].

### Glutamate release assay

The glutamate release assay was developed using previously described methods [23]. Briefly, dStr synaptosomes (n = 3, 0.3 mg/mL) were loaded into wells of a 96-well plate, placed into a fluorescent plate reader (Perkin Elmer EnSpire 2300) and incubated at 30°C for 3 min. NAD^+^ (1 mM), glutamate dehydrogenase (50 U/mL), and CaCl_2_ (1 mM) (Sigma-Aldrich NZ Ltd, Auckland) were added and baseline fluorescent recordings (excitation/emission, 340/460 nm) made every 2 s for 10 min. The K^+^-channel blocker, 4-aminopyridine (4-AP, 1 mM) was added and glutamate release measured for 5 min. A standard solution of 5 nmol of glutamate was added to quantify values.

### Protein extraction

The synaptosome pellet was dissolved in lysis buffer (40 mM Tris, 7 M urea, 2 M thiourea, 4% w/v CHAPS, and 1% protease inhibitor cocktail, P8340, Sigma-Aldrich), incubated for 60 min (4°C) with shaking, centrifuged at 15,000 x g (10 min, 4°C) and supernatants collected, protein concentrations measured using the Bradford method and stored at -20°C.

### Protein digestion

Protein samples were pooled into control or methamphetamine groups for LC-MS/MS as performed previously [13]. Briefly, proteins of equal amount (μg) from each individual were pooled (n = 6), based on sufficient protein yield from synaptosome purification for LC-MS/MS analysis A 20 μg pooled aliquot was precipitated using a Protein Precipitation Kit (Calbiochem, Germany), re-dissolved in 50 μl of buffer (8 M urea, 0.1 M Tris-HCl, pH 8.5), reduced and alkylated as previously described [24]. Proteins were diluted 3-fold with 100 mM Tris-HCl, pH 8.5, and digested with trypsin at an enzyme-to-substrate ratio of 1:50 (wt/wt, modified sequencing grade, Roche Diagnostics, NZ) in the presence of 1 mM CaCl_2_ for 16 hours (37°C). Formic acid was added (4% final concentration) and the resulting tryptic peptides purified with OMIX C18 tips (Agilent Technologies Inc, USA) and eluted into 10 μl of 70% ACN, 0.1% formic acid solution. The eluted peptides were dried and reconstructed in 0.1% formic acid enabling triplicate LC MS/MS analysis.

### LC-MS/MS

LC-MS/MS experiments used a Dionex UltiMate^TM^ 3000 RSLCnano system coupled to a LTQ Orbitrap XL via a nanospray ion source (Thermo Fisher Scientific, USA). Peptides were separated on a 75 μm×15 cm PepMap C18 column (3 μm, 300 Å Dionex) at a flow rate of 200 nL/min. A buffer gradient was constructed from 0.1% formic acid (Buffer A) and 0.1% formic acid in 80% ACN (Merck) (Buffer B): 2% B to start, 2–20% B for 60 min, 20–30% B for 162 min, 30–42% for 30 min, 42–65% for 60 min, 65–98% for 30 min, 98–2% for 5 min. The spray voltage was set at 1.8 kV, and the temperature of the heated capillary was set at 200°C. Full MS scan (*m*/*z* 200–1850) in profile mode was acquired in the Orbitrap with 30,000 resolution. The six most intense peptide ions from the full scan were selected and fragmented using CID (normalised collision energy, 35%; activation Q, 0.250; and activation time, 30 ms). Dynamic exclusion was enabled with the following settings: repeat count, 2; repeat duration, 30 s; exclusion list size, 500; exclusion duration, 90 s. The spectra were acquired using Xcalibur (version 2.1.0 SP1, Thermo Fisher Scientific). The LC-MS/MS experiments were performed in triplicate.

### Protein identification and label-free quantitation

The LC-MS/MS spectra were searched using the *Rattus norvegicus* protein database (Uniprot Knowledgebase; 37,173 entries, downloaded November 2012) using Proteome Discoverer (PD, version 1.2.0.208, Thermo Fisher Scientific). The search allowed carboxyamidomethylation of C as a fixed modification. The dynamic side chain modifications were Oxidation on M, Carbamylation on K, Acetylation on K, Deamidation on N, Q and R, Phosphorylation on R, S, T and Y, N-terminal Carbamylation and Sulfation on S, T and Y. Missed tryptic cleavage sites was 2, mass tolerance was 0.80 Da for fragment and 10.0 ppm for parent ions in monoisotopic mode. A positive identification was a peptide with high confidence and at least one peptide at rank 1 matched to a protein with the top score. The false discovery rate (FDR) was <1% using a decoy database strategy.

The PD search result files (.msf) were uploaded into Scaffold (version 4.3.2, Proteome Software Inc., USA) for protein identification and label-free quantitation based on spectral counts. The three LC-MS/MS technical replicates (n = 3) of each of the two groups (methamphetamine and control) were uploaded and combined in Scaffold and the total number of MS/MS spectra calculated [25]. The in-built spectral count normalisation function and Fischer’s exact test, an appropriate test for small sample sizes [25] were used to calculate the fold changes and p values of protein abundances between the pooled control and methamphetamine groups. Proteins detected with ≥95% probability (Protein FDR = 0.1%) assigned by ProteinProphet [26] containing at least one peptide that were detected with ≥95% probability (Peptide FDR = 0.6%) assigned by PeptideProphet [27] were considered positive identifications and quantified. A protein was considered significantly differentially-expressed if p≤0.05 by Fisher’s Exact Test [25] and the fold change was ≥ ±1.2 [15].

### Bioinformatics

The UniProt batch retrieval tool (http://uniprot.org/batch) was used to create a fasta file of the identified synaptosome proteins, which was uploaded into Wolf pSORT (http://wolfpsort.org) to classify their subcellular localisation.

WebGestalt (http://bioinfo.vanderbilt.edu/webgestalt) was used for interpreting the differentially-expressed proteins in a biological context. The protein list was uploaded into WebGestalt and searched for enrichment for GO terms using the following parameters: Reference set, *Rattus norvegicus* genome; Hypergeometric test with Benjamini and Hochberg adjustment; significance level p<0.05 and Minimum number of genes per category, 3.

Ingenuity Pathways Analysis (IPA, Ingenuity® Systems, Redwood City, CA) was used for identifying biological networks (cut-off score 20) and diseases and functions associated with the differentially-expressed proteins (p<1.00E-03). The differentially-expressed proteins and corresponding fold changes were uploaded into the Ingenuity Knowledge database. Networks of the molecular interactions between proteins, in association with biological functions and/or diseases, were reported. Proteins are displayed with their corresponding gene names and represented as nodes, whereas biological relationship between two nodes is represented with an edge (line). All edges are supported by at least one publication from information in the Ingenuity Knowledge database. The intensity of node color indicates increased (red) or decreased (green) abundance. Nodes are displayed using various shapes that represent the functional class of the protein.

### Western blotting

Supernatant S1 fractions (10 μg protein) from synaptosome purifications were used for Western blotting (control n = 5, methamphetamine self-administration n = 5). All of these samples were represented in the LC-MS/MS analysis and were chosen based on sufficient protein amount for Western blot analyses. One sample from each group had insufficient protein amount and therefore were excluded. Electrophoresed proteins were transferred to an Immobilon-FL PVDF membrane (Millipore, Thermo Fisher Scientific), blocked with 5% BSA and probed using anti-phosphatidylethanolamine binding protein-1 (Pebp1, 1/750 dilution, ab76582, Abcam) and anti-amphiphysin (Amph, 1/25,000 dilution, ab52646, Abcam) followed by anti-rabbit Cy5 (PA45011, GE Healthcare) and imaged using a Fujifilm FLA-5000 fluorescent scanner. Membranes were re-probed and normalised to α-tubulin (ab18251, Abcam). One-tailed t-tests were used to identify significant changes between control and methamphetamine groups (p<0.05).

## Results

### Methamphetamine self-administration

**Fig 1:**
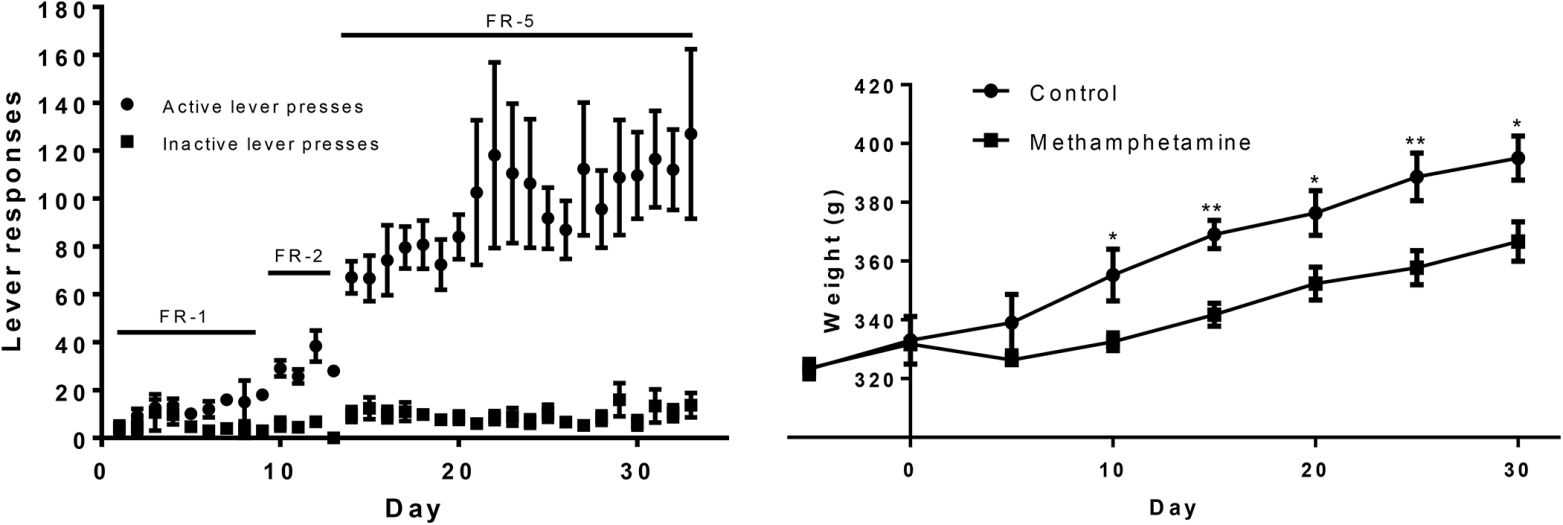
Methamphetamine self-administration. Methamphetamine self-administration lever responses showing preference for the active lever (A). Rat weight during the self-administration study (B). Methamphetamine self-administration rats (n = 8) gained less weight than controls (n = 7) with significantly reduced body weight from day 10 (*p<0.05, **p<0.01, t-test).

The acquisition rate for stable methamphetamine responding was 8/9 and one of the control rats lost catheter patency before the end of the experiment; therefore, 8 methamphetamine self-administration and 7 control rats completed the self-administration phase. Two-way ANOVA revealed significant effect between active and inactive lever responses [F(1,266) = 24.68, p = 0.0002], with significant effect over the 20 sessions [F(19,266) = 2.21, p = 0.003]. There was significant interaction between lever and time [F(19,266) = 2.12, p = 0.0047] (Fig 1A).

Rats did not display escalation of drug intake and total methamphetamine intake across the 30 days of self-administration was 12.5±0.89 mg (1.5±0.2 mg/kg/day). The control group did not develop preference for the active lever over the inactive lever.

Methamphetamine-exposed rats gained weight slower than controls during the study (Fig 1B). Two-way ANOVA showed significant effect of treatment between control and methamphetamine [F(1,396) = 8.02, p = 0.0163] over the course of the study [F(36,396) = 91.66, p<0.0001]. There was also significant interaction between treatment and time [F(36,396) = 8.26, p<0.0001]. Student’s t-test showed that body weight was significantly different between control and methamphetamine from day 10 (p<0.05).

### Synaptosome purification

**Fig 2:**
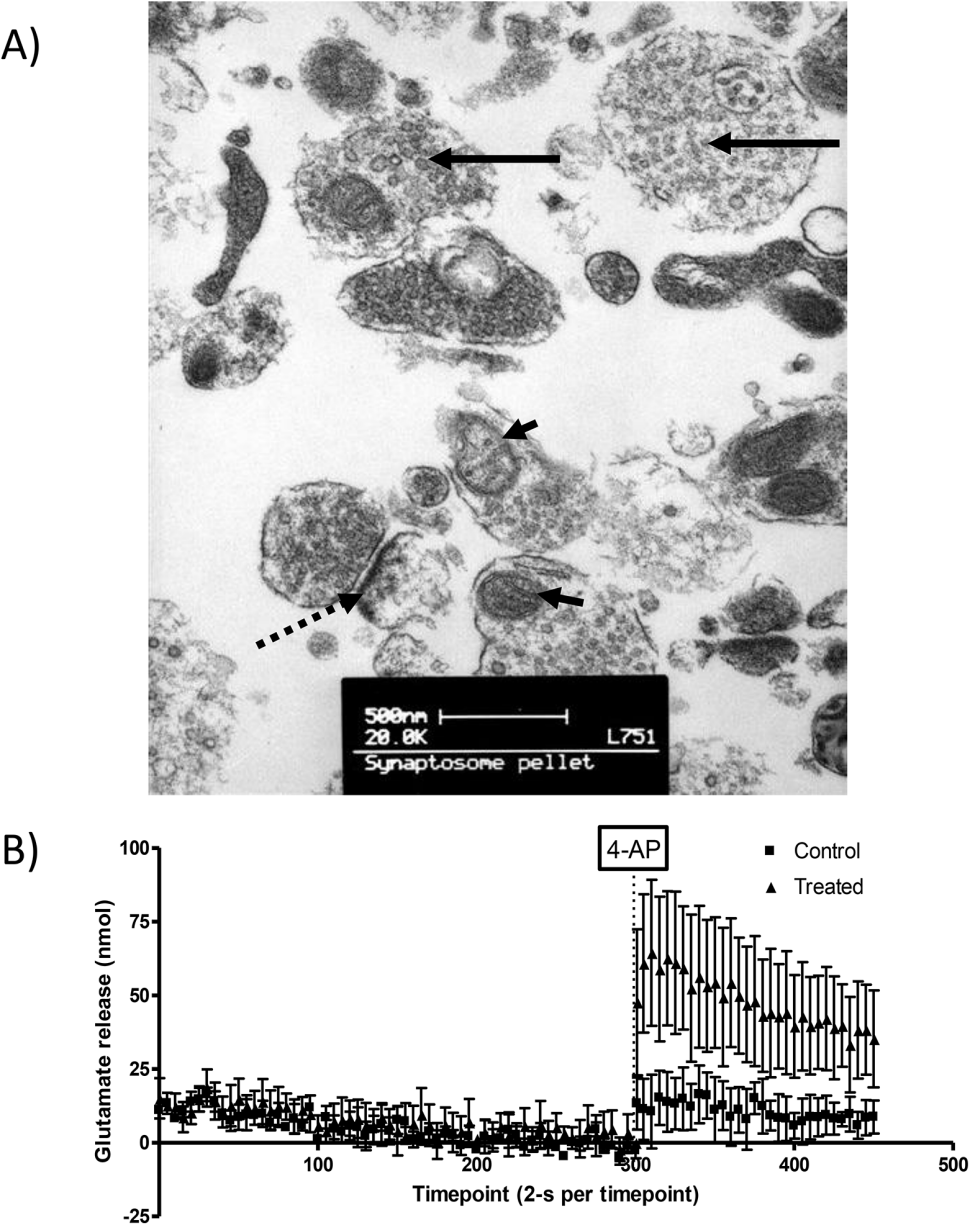
Synaptosome validation. TEM image of synaptosomes **(**A). Synaptosomes have 0.5–1 mm diameters, contain 1–2 mitochondria (arrowheads) and have many synaptic vesicles (solid arrows). There is also evidence of post-synaptic density attached to the pre-synaptic terminals (dashed arrow). Glutamate is released from dStr synaptosomes in response to the K^+^ channel blocker, 4-AP, confirming the presence of functional synaptosomes (B).

We utilised synaptosomal proteomics to identify changes in synaptic proteins following methamphetamine exposure. Synaptosomal proteomics has been successfully used to study ischaemic brain injury [28] and oxidative stress [29]. Synaptosomes imaged using TEM revealed diameters of 0.5–1 μm with visible mitochondria, synaptic vesicles and post-synaptic density (Fig 2A). A glutamate release assay (Fig 2B) showed that synaptosomes released glutamate in response to 4-AP, demonstrating synaptosomes used for proteomics analysis have maintained membrane integrity and synaptic function. Purified tissue fractions from each group of animals was pooled before being subjected to proteomics analysis of 3 technical replicates using LC-MS/MS.

### Protein identification of synaptosome samples

A total of 423 (control) and 441 (methamphetamine) proteins were unambiguously identified in the synaptosome samples (S1 Table). The datasets are available at ProteomeXchange (http://proteomecentral.proteomexchange.org/cgi/GetDataset) with accession numbers via the PRIDE partner repository [30] with the dataset identifier PXD001443 and DOI 10.6019/PXD001443.

Of these, 406 were common to both sets, accounting for 92% and 96% of the overall proteins identified (S1 Fig), indicating high correlation between the two biological conditions. Functional analyses using Wolf pSORT showed that the composition of control and methamphetamine samples are comparable to each other (S1 Fig) and reflected the expected composition of synaptosomes from previous reports [31]. For control samples, the composition was 2% cytoskeleton, 19% mitochondria, 7% membrane and 64% cytoplasm and for methamphetamine samples, the composition was 2% cytoskeleton, 18% mitochondria, 8% membrane and 66% cytoplasm.

### Differentially-expressed proteins

Label-free quantitation based on spectral counts revealed 84 differentially-expressed proteins, with 42 upregulated and 42 downregulated between control and methamphetamine (Table 1 and S2 Table). Most of these proteins were unambiguously identified with more than two unique peptides. We did not identify any significantly different post-translational modifications. The spectra of the three proteins, Guk1, Pdxk, Vcan, that were identified with one peptide in the control sample were manually examined and confirmed the positive identifications of these peptides (S2 Fig).

**Table 1:** Differentially-expressed proteins in synaptosomes in rats following methamphetamine self-administration.

				Normalised spectral count		Unique peptides				
Protein	Gene name	Accession number	M_r_ (kDa)	Control	Meth	Control	Meth	p-value*	ER	Fold change
***Mitochondria/Energy***										
ATPase inhibitor, mitochondrial	Atpif1	Q03344	12	0.00	7.72	0	4	0.0052	N/A	INF
NAD-dependent protein deacylase sirtuin-5, mitochondrial	Sirt5	Q68FX9	34	0.00	7.72	0	4	0.0052	N/A	INF
Fructose-bisphosphate aldolase A	Aldoa	P05065	39	240.88	332.73	19	20	< 0.00010	1.4	1.4
ATP synthase subunit beta, mitochondrial	Atp5b	P10719	56	500.46	384.81	25	24	< 0.00010	0.77	-1.3
Malate dehydrogenase, mitochondrial	Mdh2	P04636	36	133.94	98.37	12	12	0.011	0.71	-1.4
Aconitate hydratase, mitochondrial	Aco2	Q9ER34	85	125.63	87.76	23	20	0.0055	0.71	-1.4
Cytochrome b-c1 complex subunit 1, mitochondrial	Uqcrc1	QCR1	53	48.80	30.86	11	9	0.028	0.59	-1.7
NADH-ubiquinone oxidoreductase 75 kDa subunit, mitochondrial	Ndufs1	Q66HF1	79	52.95	32.79	15	11	0.019	0.59	-1.7
Aspartate aminotransferase, mitochondrial	Got2	P00507	47	18.69	8.68	8	3	0.041	0.5	-2.0
10 kDa heat shock protein, mitochondrial	Hspe1	P26772	11	35.30	19.29	3	3	0.02	0.5	-2.0
Acetyl-CoA acetyltransferase, mitochondrial	Acat1	P17764	45	30.11	14.47	8	6	0.013	0.5	-2.0
NADH dehydrogenase (Ubiquinone) flavoprotein 1	Ndufv1	Q5XIH3	51	21.80	7.72	6	5	0.0071	0.4	-2.5
Dihydrolipoyl dehydrogenase, mitochondrial	Dld	Q6P6R2	54	21.80	7.72	6	2	0.0071	0.4	-2.5
Cytochrome b-c1 complex subunit Rieske, mitochondrial	Uqcrfs1	P20788	29	11.42	3.86	3	2	0.044	0.3	-3.3
Mitochondrial inner membrane protein (Fragment)	Immt	Q3KR86	67	15.57	4.82	4	3	0.014	0.3	-3.3
NADH dehydrogenase (Ubiquinone) Fe-S protein 5	Ndufs5	B5DEL8	13	19.73	34.72	3	5	0.029	1.8	1.8
Triosephosphate isomerase	Tpi1	P48500	27	196.24	245.93	15	15	0.0099	1.3	1.3
Cytochrome c oxidase subunit 2	Mtco2	P00406	26	6.23	0.00	2	0	0.012	N/A	−INF
Aspartate aminotransferase, cytoplasmic	Got1	P13221	46	43.61	21.22	10	8	0.0036	0.5	-2.0
Guanylate kinase	Guk1	Q71RR7	22	1.04	7.72	1	3	0.025	7.4	7.4
Sodium/potassium-transporting ATPase subunit alpha-3	Atp1a3	P06687	112	49.84	19.29	13	7	0.00015	0.4	-2.5
Glyceraldehyde-3-phosphate dehydrogenase	Gapdh	G3P	36	201.43	168.78	14	15	0.049	0.77	-1.3
***Synaptic vesicles***										
Endophilin-B2	Sh3glb2	D4A7V1	45	21.80	48.22	3	11	0.0011	2.2	2.2
Endophilin-A1 (fragment)	Sh3gl2	F1LQ05	38	94.48	72.33	10	10	0.05	0.77	-1.3
Transitional endoplasmic reticulum ATPase	Vcp	P46462	89	52.95	81.98	18	21	0.0078	1.5	1.5
Amphiphysin	Amph	O08838	75	59.18	94.52	15	20	0.0027	1.6	1.6
Dynactin subunit 2	Dctn2	Q6AYH5	44	32.19	49.19	10	9	0.038	1.5	1.5
Cofilin-1	Cfl1	P45592	19	124.59	155.27	10	13	0.038	1.2	1.2
V-type proton ATPase subunit B, brain isoform	Atp6v1b2	P62815	57	248.15	303.80	23	27	0.0095	1.2	1.2
Protein bassoon	Bsn	G3V984	418	47.76	68.48	17	25	0.034	1.4	1.4
Dynamin-1	Dnm1	P21575	97	123.56	95.48	27	27	0.033	0.77	-1.3
Synaptotagmin-1	Syt1	P21707	47	5.19	0.00	3	0	0.026	N/A	−INF
Rab GDP dissociation inhibitor beta	Gdi2	P50399	51	0.00	26.04	0	2	< 0.00010	N/A	INF
Toll-interacting protein	Tollip	A2RUW1	30	0.00	7.72	0	3	0.0052	N/A	INF
Protein Tom1	Tom1	Q5XI21	54	3.11	10.61	2	4	0.039	3.4	3.4
Isoform Glt-1A of Excitatory amino acid transporter 2	Slc1a2	P31596-2	62	31.15	5.79	7	3	< 0.00010	0.2	-5.0
***Cytoskeleton***										
A-kinase anchor protein 5	Akap5	P24587	76	2.08	8.68	2	2	0.043	4.2	4.2
Tropomyosin alpha-3 chain	Tpm3	Q63610	29	41.53	61.72	11	15	0.029	1.5	1.5
Tubulin alpha-4A chain	Tuba4a	Q5XIF6	50	220.12	179.38	4	4	0.023	0.77	-1.3
Tubulin alpha-1A chain	Tuba1a	P68370	50	230.50	187.10	19	20	0.018	0.77	-1.3
Tubulin alpha-1B chain	Tuba1b	Q6P9V9	50	242.96	192.89	2	2	0.0089	0.77	-1.3
Actin, cytoplasmic 1	Actb	P60711	42	232.58	192.89	19	18	0.029	0.77	-1.3
14-3-3 protein epsilon	Ywhae	P62260	29	31.15	16.40	4	4	0.022	0.5	-2.0
14-3-3 protein eta	Ywhah	P68511	28	29.07	14.47	3	4	0.019	0.5	-2.0
14-3-3 protein theta	Ywhaq	P68255	28	18.69	4.82	4	3	0.0032	0.3	-3.3
Isoform 5 of Tropomyosin alpha-1 chain	Tpm1	P04692-5	28	27.00	54.01	4	5	0.0018	2.0	2.0
Protein Tppp	Tppp	D3ZQL7	24	70.60	96.44	8	10	0.027	1.4	1.4
2',3'-cyclic-nucleotide 3'-phosphodiesterase	Cnp	P13233	47	12.46	2.89	4	2	0.012	0.2	-5.0
Protein Cttn	Cttn	D3ZGE6	53	15.57	33.76	4	9	0.007	2.2	2.2
***Neuroprotection***										
Superoxide dismutase [Cu-Zn]	Sod1	Q6LDS4	16	45.69	81.98	5	10	0.00085	1.8	1.8
Oxidation resistance protein 1	Oxr1	Q4V8B0	93	24.92	40.51	8	10	0.036	1.6	1.6
Stress-induced-phosphoprotein 1	Stip1	R9PXW7	63	18.69	37.61	7	13	0.0082	2.0	2.0
Prohibitin	Phb	P67779	30	10.38	1.93	3	2	0.014	0.2	-5.0
***Neuroplasticity***										
Dihydropyrimidinase-related protein 4 (Fragment)	Dpysl4	Q62951	61	17.65	32.79	8	12	0.023	1.9	1.9
Dihydropyrimidinase-related protein 5	Dpysl5	Q9JHU0	62	21.80	10.61	6	6	0.035	0.5	-2.0
***Cell signalling***										
Calcium/calmodulin-dependent protein kinase type II subunit alpha	Camk2a	P11275	54	125.63	95.48	12	12	0.024	0.77	-1.3
Protein kinase C gamma type	Prkcg	P63319	78	0.00	4.82	0	2	0.037	N/A	INF
Serine/threonine-protein phosphatase 2A 55 kDa regulatory subunit B alpha isoform	Ppp2r2a	P36876	52	5.19	23.15	3	7	0.00055	4.5	4.5
Protein phosphatase 1 regulatory subunit 1B	Ppp1r1b	Q6J4I0	23	27.00	44.36	6	6	0.026	1.6	1.6
Guanine nucleotide-binding protein subunit beta-5	Gnb5	P62882	39	5.19	0.00	2	0	0.026	N/A	−INF
MOB-like protein phocein	Mob4	Q9QYW3	26	0.00	4.82	0	3	0.037	N/A	INF
Serine/threonine-protein kinase PAK 1	Pak1	P35465	61	4.15	25.08	2	7	< 0.00010	6.0	6.0
RCG61894, isoform CRA_a	Strn	G3V6L8	86	6.23	20.25	3	8	0.0052	3.3	3.3
***Neuroconnections***										
Neural cell adhesion molecule 1 (Fragment)	Ncam1	F1LNY3	93	9.34	1.93	4	2	0.025	0.2	-5.0
Neuronal cell adhesion molecule long isoform Nc17	Nrcam	Q6PW34	143	14.54	5.79	6	4	0.041	0.4	-2.5
Synapsin-1	Syn1	P09951	74	127.71	95.48	22	19	0.018	0.71	-1.4
Synapsin-2	Syn2	G3V733	61	90.33	59.80	12	12	0.0076	0.71	-1.4
Isoform 1 of SH3 and multiple ankyrin repeat domains protein 3	Shank3	Q9JLU4-2	192	37.38	54.97	13	18	0.042	1.5	1.5
***Protein folding/degradation/repair***										
Ubiquitin carboxyl-terminal hydrolase isozyme L1	Uchl1	Q00981	25	74.76	113.80	10	10	0.0027	1.5	1.5
Peptidyl-prolyl cis-trans isomerase A	Ppia	P10111	18	101.75	130.20	9	8	0.035	1.3	1.3
T-complex protein 1 subunit epsilon	Cct5	Q68FQ0	60	5.19	0.00	3	0	0.026	N/A	−INF
Protein Ubqln2	Ubqln2	D4AA63	67	12.46	26.04	4	7	0.021	2.1	2.1
Protein-L-isoaspartate(D-aspartate) O-methyltransferase	Pcmt1	P22062	25	22.84	8.68	5	3	0.0086	0.4	-2.5
***Other***										
Phosphatidylethanolamine-binding protein 1	Pebp1	P31044	21	118.36	156.24	10	15	0.013	1.3	1.3
Nucleoside diphosphate kinase A	Nme1	Q05982	17	11.42	0.00	4	0	0.00032	N/A	−INF
Pyridoxal kinase	Pdxk	G3V647	35	1.04	6.75	1	4	0.044	6.5	6.5
Serum albumin	Alb	P02770	69	0.00	6.75	0	3	0.01	N/A	INF
Isoform V3 of Versican core protein	Vcan	Q9ERB4-2	74	3.11	11.57	1	6	0.025	3.7	3.7
Cysteine-rich protein 2	Crip2	P36201	23	5.19	19.29	2	4	0.0035	3.7	3.7
Protein RGD1559864	RGD1559864	D3ZB78	41	6.23	17.36	5	6	0.018	2.8	2.8
Uncharacterized protein	4 SV	D4A269	14	7.27	17.36	3	4	0.033	2.4	2.4
Carbonic anhydrase 2	Ca2	P27139	29	6.23	0.00	2	0	0.012	N/A	−INF

*Fischer’s exact test. Fold change: the ratio of normalized spectral counts between Meth and Control when the protein is upregulated in Meth, or the negative reciprocal of the above ratio when the protein is downregulated in Meth; ER: Expression ratio; INF: infinite.

### Western blotting

**Fig 3:**
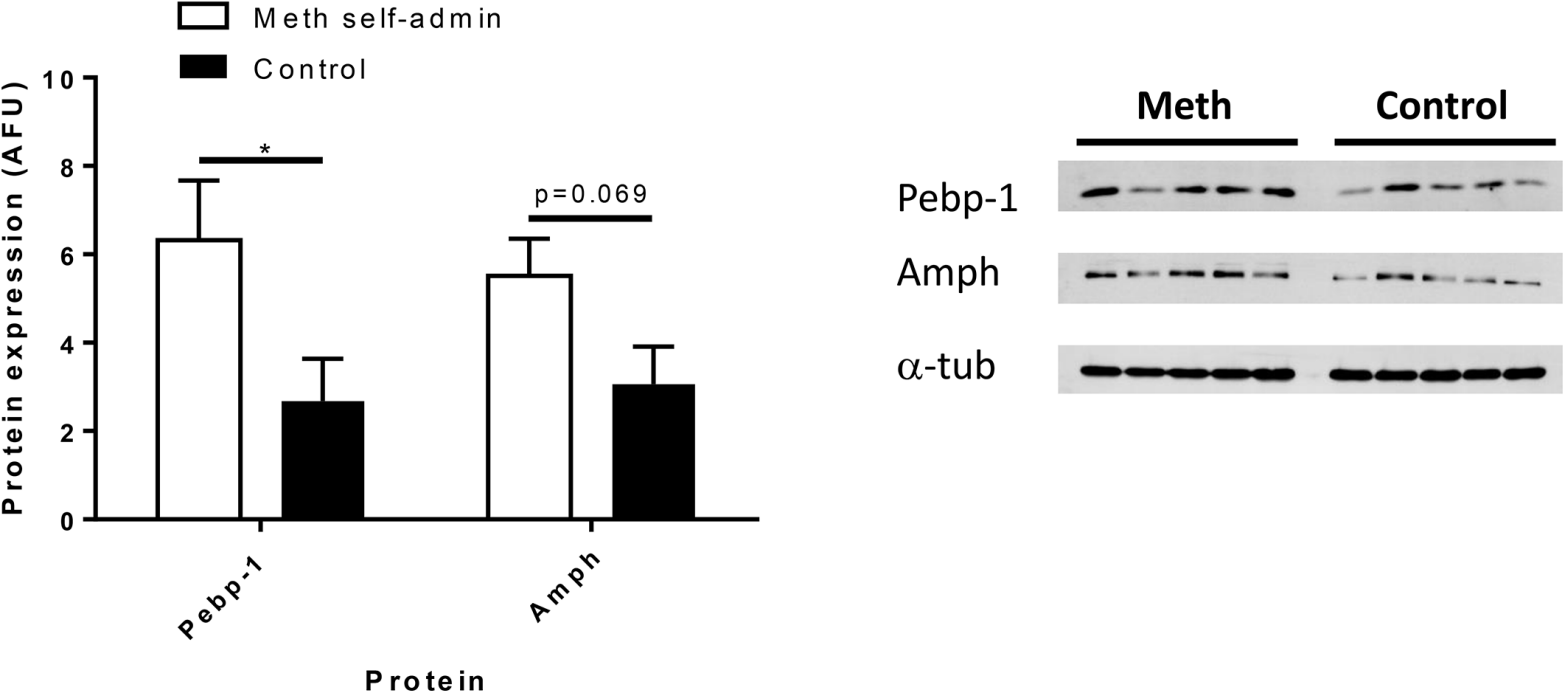
Validation of protein expression. Western blotting showed upregulation of phosphatidylethanolamine binding protein (*p<0.05, 1-tailed t-test, n = 5) and a trend towards increased amphiphysin (p = 0.069, 1-tailed t-test, n = 5). Protein expression was normalized to alpha-tubulin. Key: Amph, amphiphysin; Pebp1, phosphatidylethanolamine binding protein; α-tub, alpha tubulin.

Two differentially-expressed synaptic proteins, Amphiphysin (Amph) and Phosphatidylethanolamine binding protein-1 (Pebp1), detected by LC-MS/MS were chosen for validation of the quantitative LC-MS/MS data using Western blotting. These were chosen based on previous links to the literature and represented proteins important for synaptic vesicles and synapse formation. Experiments showed agreement with the LC-MS/MS results for Amph (p = 0.069, 1-tailed t-test, n = 5) and Pebp1 (p<0.05, 1-tailed t-test, n = 5) using proteins from the supernatant S1 fraction (Fig 3, Table 1), indicating the reliability of the LC-MS/MS data. Additional validation of Amph utilising dStr homogenate samples also revealed a significant increase in protein expression following methamphetamine when compared to controls (p<0.05) (data not shown.).

### Functional analysis of the differentially-expressed proteins

The GO functions of the differentially-expressed proteins annotated with WebGestalt were, mitochondria/energy, cytoskeleton, synaptic vesicles, cell signalling, neuroplasticity, protein folding/degradation, neuroprotection and others (Table 1). Pathways analysis of the differentially-expressed proteins using IPA revealed involvement of these proteins in molecular interaction networks, including cell-to-cell signalling and interaction, nervous system development and function, cellular assembly and organisation, cell morphology, cellular development and cellular compromise (S3 Fig). These networks are associated with neurological disease, microtubule dynamics and cellular protrusions (S3 Table). The core group of proteins participating in these networks are primarily synaptic vesicle and cytoskeletal proteins.

## Discussion

This study is the first to report the use of MS-based proteomics to examine changes in synaptic protein abundance in the dorsal striatum following methamphetamine self-administration in rats. Methamphetamine is transported into the synapse through the dopamine transporter and has extensive detrimental effects, particularly on mitochondrial function [3]. The dStr is important as it assumes greater control over drug addiction behaviour as drug taking becomes compulsive [32]. We employed subcellular fractionation to enrich for synaptosomal proteins and label-free LC-MS/MS to determine the differentially-expressed proteins following methamphetamine exposure. We applied the pooling strategy [13] to identify the largest changes between the two groups. We chose this strategy to reduce technical variation in the label-free quantification of the samples introduced during LC-MS/MS runs due to chromatographical drift and instrumental maintenance requirements. Each LC-MS/MS run requires approximately 6 hours and the mass spectrometer also requires weekly calibration, which interrupts the sample analysis. Because this approach limits between-animal analysis, selected proteins from individual animals were validated using Western blot analysis.

Amongst the interesting differentially-expressed proteins were Amphiphysin (Amph), Phosphatidylethanolamine binding protein-1 (Pebp1) and stress-induced phosphoprotein. Amph is a Bin-amphiphysin-Rvs (BAR) domain protein, which are a group of proteins that function to create a bend in the plasma membrane prior to vesicle fusion, and also aid in the scaffolding of synaptic vesicles [33]. Amph mRNA has been reported to increase following methamphetamine administration in the cerebrum and cerebellum of the rat [34]. The importance of Amph in the brain is exemplified by learning deficits in knockout mice [35], which also display defects in synaptic recycling and cognitive impairment [36]. Other synaptic vesicle proteins were up-regulated that have demonstrated roles in synaptic vesicle budding and fusion and acidification of vesicles, including, endophilin-B2, transitional ER ATPase and the B-subunit of V-type proton ATPase [33,37,38]. The upregulation of these proteins suggests increased synaptic vesicle production or activity and may be a consequence of altered accumulation and storage of dopamine [38] following repeated methamphetamine exposure.

Pebp1, a serine protease inhibitor implicated in neuronal growth, differentiation and synapse production was upregulated [39]. Although previous reports show downregulation with methamphetamine behavioural sensitisation [40] and self-administration following extinction [16], up-regulation following cocaine self-administration followed by 100 days abstinence has been reported [15]. Interestingly, our results vary from a recent publication which found reduced Pebp1 following methamphetamine self-administration and extinction training [16]. It is possible that this difference is due to different brain regions examined (pre-frontal cortex vs dorsal striatum), although this may also reflect the new learning of a non-reinforcer in extinction when compared with abstinence, where all association with the self-administration chamber is removed. Pebp1 may therefore represent an important response to psychostimulant self-administration persisting into abstinence. Future investigations into the signalling pathways regulating Pebp1 may help elucidate the role of Pebp1 in methamphetamine addiction and abstinence.

Proteins with known neuroprotective roles, peroxiredoxin-6, oxidation resistance protein and superoxide dismutase were upregulated along with Stip1, a protein that interacts with the prion protein PrP(C) to stimulate protein synthesis in neurons. Stress-induced phosphoprotein -PrP(C) is involved in neuroprotection and neuron plasticity [41] and protects astrocytes from cell death [42].

Rearrangement of synaptic architecture occurs during both self-administration and abstinence [43], and increased arborisation of viable synaptic terminals may occur during abstinence following methamphetamine [44]. Amphetamines increase dendritic branching in the brain due to restructuring of the cell cytoskeleton [45], and methamphetamine behavioural sensitisation increases synaptic density in the nucleus accumbens [43]. Several proteins associated with axon branching, Pak1, Cttn, Phocein, and Shank3 were up-regulated.

Cytoskeleton and associated proteins including increased cofilin-1, tubulin polymerisation-promoting protein (Tppp), Cttn and tropomyosin-α; and decreased 14-3-3 isoforms, plus tubulin and actin with methamphetamine administration were also seen. Previous studies found large decreases in tubulin with methamphetamine behavioural sensitisation [40], and overexpression of cytoskeletal proteins following abstinence from amphetamine self-administration [14]. Cofilin-1 and Tppp were upregulated with methamphetamine self-administration, which bind to actin and tubulin monomers respectively and are thus involved in cytoskeletal protein stabilisation. Little is known about Tppp, as no links with methamphetamine administration have been reported.

Mitochondrial dysfunction is a well-established consequence of methamphetamine exposure as neurons are very sensitive to reduced ATP [3]. Our study supports this, with differentially-expressed proteins associated with mitochondria and energy regulation.

### Significance

Our results correlate well with a previous cocaine self-administration and abstinence study with 4 of the 12 proteins (Pebp1, ATP synthase beta subunit, Malate dehydrogenase and dynamin-1) corresponding [15]. Our results also correspond well with previous studies of brain proteomics in rats trained for methamphetamine conditioned place preference [46], and methamphetamine behavioural sensitisation [40], which identified differentially-expressed proteins involved in cytoskeletal rearrangement, signal transduction and synaptic function. Higher dosing regimens traditionally associated with neurotoxicity, in contrast, displayed a different proteomics profile in the prefrontal cortex following methamphetamine (8x1 mg/kg), where protein degradation, energy metabolism, synaptic function and cytoskeletal rearrangement pathways were highly represented [10]. In addition, a further proteomics study identified differentially-expressed proteins in the striatum (14), hippocampus (12) and frontal cortex (4) following methamphetamine administration (8x15 mg/kg, 12 hours apart), with common proteins altered in the different regions [12].

## Conclusions

The methamphetamine self-administration model employed in this proteomics study identified changes that suggest a combination of cell stress with synaptic plasticity and neuroadaptation. The model used represents drug-taking, and in future could include compulsive drug-taking and drug-seeking models. Further studies will aim to utilise yoking controls to separate protein expression changes related to the motivational aspects of drug-taking behaviour and also to identify those important during abstinence. This study provides a number of key targets which provide useful mechanistic insight into the effects of methamphetamine on dStr synaptic proteins, which can be pursued in detail with a model of drug addiction that more accurately reflects human experience. The detailed understanding of synaptic proteins in response to drugs of addiction may also allow the identification of future therapeutic targets.

## Supporting Information

S1 Fig Protein identifications of the purified synaptosome samples of the control and methamphetamine treated rats (A). Subcellular localisation of synaptosome proteins for control and methamphetamine treated rats analysed using Wolf pSORT showing a distribution of proteins that is consistent with being synaptosomal (B). (PDF)

S2 Fig Spectra for single-peptide identifications. Spectra for single-peptide identifications shown in Table 1 and S2 Table.
(PDF)

S3 Fig Networks of differentially-expressed proteins. Proteins shaded in green indicate down-regulation and red means up-regulation. The intensity of the shade corresponds to the degree of up (red) or down (green) regulation. Proteins in white are those identified through the Ingenuity Pathways Knowledge Base. The shapes denote the molecular class of the protein. A solid line indicates a direct molecular interaction, and a dashed line indicates an indirect molecular interaction. The cut-off score of network identification was 20. Cell-to-cell signaling and interaction, nervous system development and function and cellular assembly and organization, IPA score = 55 (A); Cell morphology, cellular assembly and organization and cellular development, IPA score = 31 (B); Cellular compromise, cell morphology, cellular assembly and organization, IPA score = 21 (C).
(PDF)

S1 Table All proteins identified. (PDF)

S2 Table Differentially-expressed proteins. (PDF)

S3 Table Diseases and functions associated with the differentially expressed proteins. (PDF)

S4 Table ARRIVE Guidelines Checklist. (PDF)
