# Supplementary material for: Proteomics Analysis of Dorsal Striatum Reveals Changes in Synaptosomal Proteins following Methamphetamine Self-Administration in Rats
Source: PLoS One. 2015 Oct 20;10(10):e0139829. doi: 10.1371/journal.pone.0139829 (PMC4618287; doi:10.1371/journal.pone.0139829)

Supplementary Figure S1: A) Protein identifications of the purified synaptosome samples of the control and methamphetamine treated rats. B) Subcellular localisation of synaptosome proteins for control and methamphetamine treated rats analysed using Wolf pSORT showing a distribution of proteins that is consistent with being synaptosomal.

A)

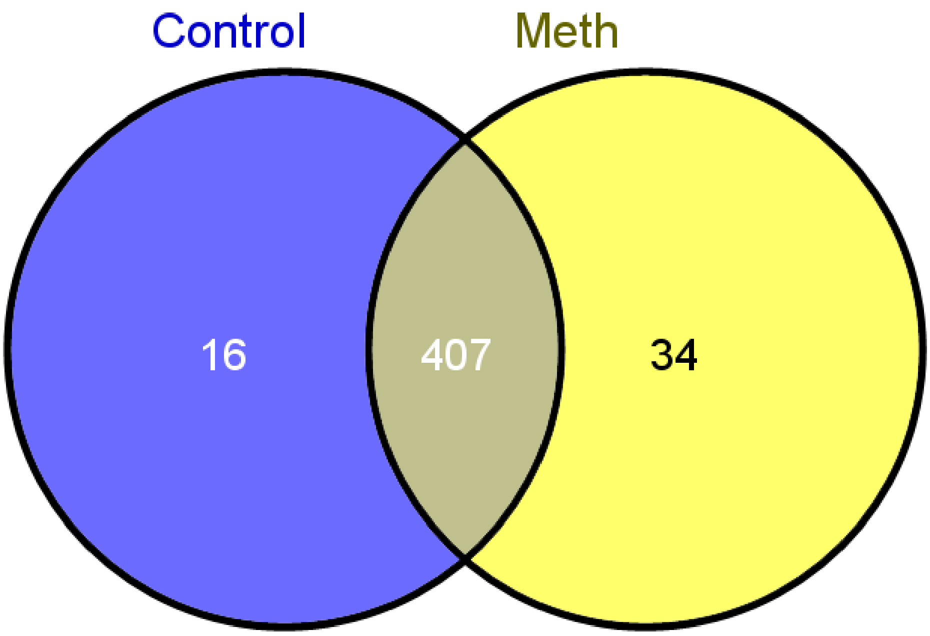

B)

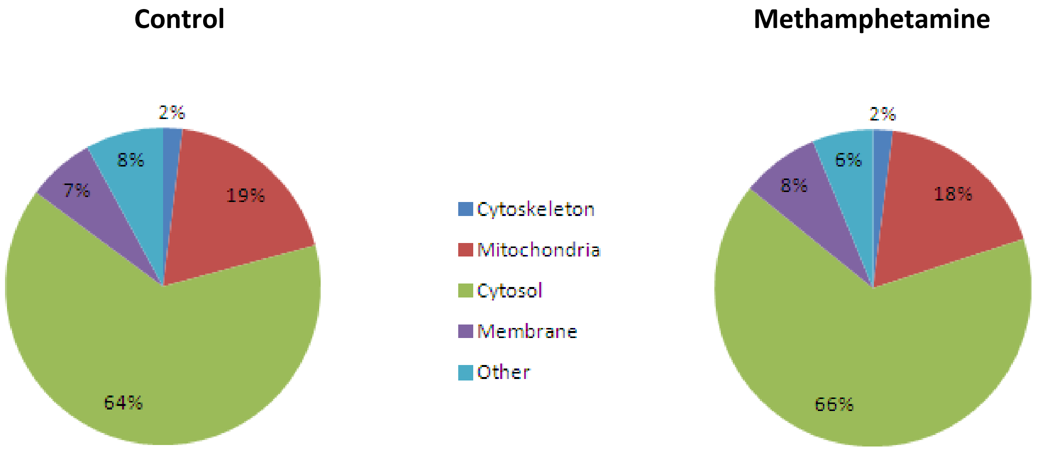

Supplement: S1 Fig — (PDF) [file pone.0139829.s001.pdf]
