# Supplementary material for: Proteomics Analysis of Dorsal Striatum Reveals Changes in Synaptosomal Proteins following Methamphetamine Self-Administration in Rats
Source: PLoS One. 2015 Oct 20;10(10):e0139829. doi: 10.1371/journal.pone.0139829 (PMC4618287; doi:10.1371/journal.pone.0139829)
Supplement: S2 Fig — Spectra for single-peptide identifications shown in Table 1 and S2 Table. (PDF) [file pone.0139829.s002.pdf]

<u>S2 Figure</u>

Spectra for single-peptide identifications

Isoform V3 of Versican core protein – Control sample

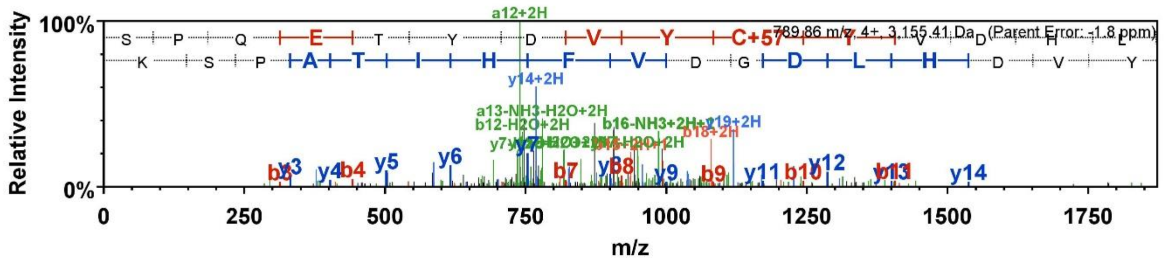

B	B Ions	B+2H	B-NH3	B-H2O	AA	Y Ions	Y+2H	Y-NH3	Y-H2O	Y
1	88.0	44.5		70.0	S	3,156.4	1,578.7	3,139.4	3,138.4	27
2	185.1	93.0		167.1	P	3,069.4	1,535.2	3,052.4	3,051.4	26
3	313.2	157.1	296.1	295.1	Q	2,972.3	1,486.7	2,955.3	2,954.3	25
4	442.2	221.6	425.2	424.2	E	2,844.3	1,422.6	2,827.3	2,826.3	24
5	543.2	272.1	526.2	525.2	T	2,715.2	1,358.1	2,698.2	2,697.2	23
6	706.3	353.7	689.3	688.3	Y	2,614.2	1,307.6	2,597.2	2,596.2	22
7	821.3	411.2	804.3	803.3	D	2,451.1	1,226.1	2,434.1	2,433.1	21
8	920.4	460.7	903.4	902.4	V	2,336.1	1,168.6	2,319.1	2,318.1	20
9	1,083.5	542.2	1,066.4	1,065.5	Y	2,237.0	1,119.0	2,220.0	2,219.0	19
10	1,243.5	622.3	1,226.5	1,225.5	C+57	2,074.0	1,037.5	2,056.9	2,056.0	18
11	1,406.6	703.8	1,389.5	1,388.5	Y	1,913.9	957.5	1,896.9	1,895.9	17
12	1,505.6	753.3	1,488.6	1,487.6	V	1,750.9	875.9	1,733.8	1,732.9	16
13	1,620.7	810.8	1,603.6	1,602.6	D	1,651.8	826.4	1,634.8	1,633.8	15
14	1,757.7	879.4	1,740.7	1,739.7	H	1,536.8	768.9	1,519.8	1,518.8	14
15	1,870.8	935.9	1,853.8	1,852.8	L	1,399.7	700.4	1,382.7	1,381.7	13
16	1,985.8	993.4	1,968.8	1,967.8	D	1,286.6	643.8	1,269.6	1,268.6	12
17	2,042.8	1,021.9	2,025.8	2,024.8	G	1,171.6	586.3	1,154.6	1,153.6	11
18	2,157.9	1,079.4	2,140.8	2,139.9	D	1,114.6	557.8	1,097.6	1,096.6	10
19	2,256.9	1,129.0	2,239.9	2,238.9	V	999.6	500.3	982.5	981.6	9
20	2,404.0	1,202.5	2,387.0	2,386.0	F	900.5	450.8	883.5	882.5	8
21	2,541.1	1,271.0	2,524.0	2,523.1	H	753.4	377.2	736.4	735.4	7
22	2,654.2	1,327.6	2,637.1	2,636.1	I	616.4	308.7	599.3	598.4	6
23	2,755.2	1,378.1	2,738.2	2,737.2	T	503.3	252.1	486.3	485.3	5
24	2,826.2	1,413.6	2,809.2	2,808.2	A	402.2	201.6	385.2	384.2	4
25	2,923.3	1,462.1	2,906.3	2,905.3	P	331.2	166.1	314.2	313.2	3
26	3,010.3	1,505.7	2,993.3	2,992.3	S	234.1	117.6	217.1	216.1	2
27	3,156.4	1,578.7	3,139.4	3,138.4	K	147.1	74.1	130.1		1

Pyridoxal kinase – Control sample

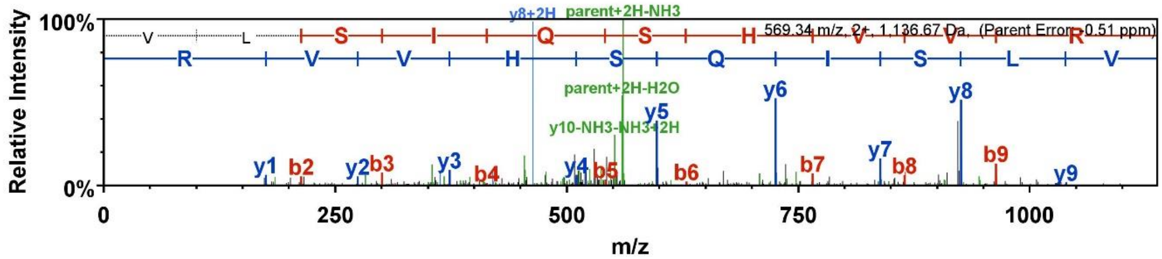

B	B Ions	B+2H	B-NH3	B-H2O	AA	Y Ions	Y+2H	Y-NH3	Y-H2O	Y
1	100.1				V	1,137.7	569.3	1,120.6	1,119.7	10
2	213.2				L	1,038.6	519.8	1,021.6	1,020.6	9
3	300.2			282.2	S	925.5	463.3	908.5	907.5	8
4	413.3			395.3	I	838.5	419.7	821.5	820.5	7
5	541.3		524.3	523.3	Q	725.4	363.2	708.4	707.4	6
6	628.4	314.7	611.3	610.4	S	597.3	299.2	580.3	579.3	5
7	765.4	383.2	748.4	747.4	H	510.3	255.7	493.3		4
8	864.5	432.8	847.5	846.5	V	373.3		356.2		3
9	963.6	482.3	946.5	945.6	V	274.2		257.2		2
10	1,137.7	569.3	1,120.6	1,119.7	R	175.1		158.1		1

Guanylate kinase – Control sample

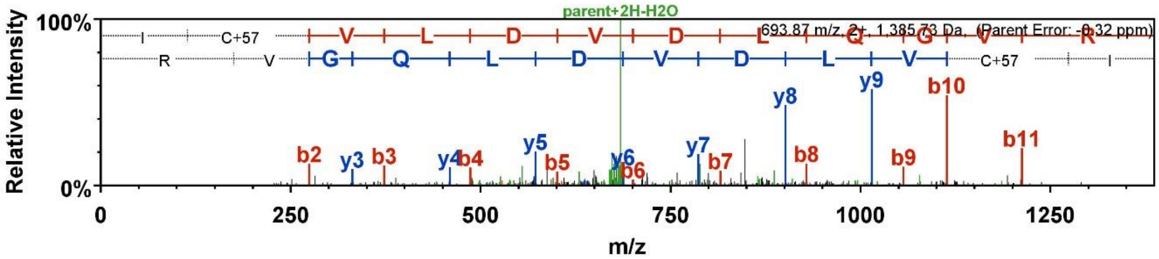

B	B Ions	B+2H	B-NH3	B-H2O	AA	Y Ions	Y+2H	Y-NH3	Y-H2O	Y
1	114.1				I	1,386.7	693.9	1,369.7	1,368.7	12
2	274.1				C+57	1,273.7	637.3	1,256.6	1,255.6	11
3	373.2				V	1,113.6	557.3	1,096.6	1,095.6	10
4	486.3				L	1,014.6	507.8	997.5	996.5	9
5	601.3			583.3	D	901.5	451.2	884.4	883.5	8
6	700.4	350.7		682.4	V	786.4	393.7	769.4	768.4	7
7	815.4	408.2		797.4	D	687.4	344.2	670.4	669.4	6
8	928.5	464.7		910.5	L	572.4		555.3		5
9	1,056.5	528.8	1,039.5	1,038.5	Q	459.3		442.2		4
10	1,113.6	557.3	1,096.5	1,095.6	G	331.2		314.2		3
11	1,212.6	606.8	1,195.6	1,194.6	V	274.2		257.2		2
12	1,386.7	693.9	1,369.7	1,368.7	R	175.1		158.1		1
