# Supplementary material for: Proteomics Analysis of Dorsal Striatum Reveals Changes in Synaptosomal Proteins following Methamphetamine Self-Administration in Rats
Source: PLoS One. 2015 Oct 20;10(10):e0139829. doi: 10.1371/journal.pone.0139829 (PMC4618287; doi:10.1371/journal.pone.0139829)
Supplement: S3 Fig — Proteins shaded in green indicate down-regulation and red means up-regulation. The intensity of the shade corresponds to the degree of up (red) or down (green) regulation. Proteins in white are those identified through the Ingenuity Pathways Knowledge Base. The shapes denote the molecular class of the protein. A solid line indicates a direct molecular interaction, and a dashed line indicates an indirect molecular interaction. The cut-off score of network identification was 20. Cell-to-cell signaling and interaction, nervous system development and function and cellular assembly and organization, IPA score = 55 (A); Cell morphology, cellular assembly and organization and cellular development, IPA score = 31 (B); Cellular compromise, cell morphology, cellular assembly and organization, IPA score = 21 (C). (PDF) [file pone.0139829.s003.pdf]

Supplementary Figure S3. Networks of differentially expressed proteins involved. (A) Cell-to-cell signaling and interaction, nervous system development and function and cellular assembly and organization, IPA score = 55; **(B)** Cell morphology, cellular assembly and organization and cellular development, IPA score = 31; (C) Cellular compromise, cell morphology, cellular assembly and organization, IPA score = 21. Proteins shaded in green indicate down-regulation and red means up-regulation. The intensity of the shade corresponds to the degree of up (*red*) or down (*green*) regulation. Proteins in white are those identified through the Ingenuity Pathways Knowledge Base. The shapes denote the molecular class of the protein. A solid line indicates a direct molecular interaction, and a dashed line indicates an indirect molecular interaction. The cut-off score of network identification was 20.

Network (A)

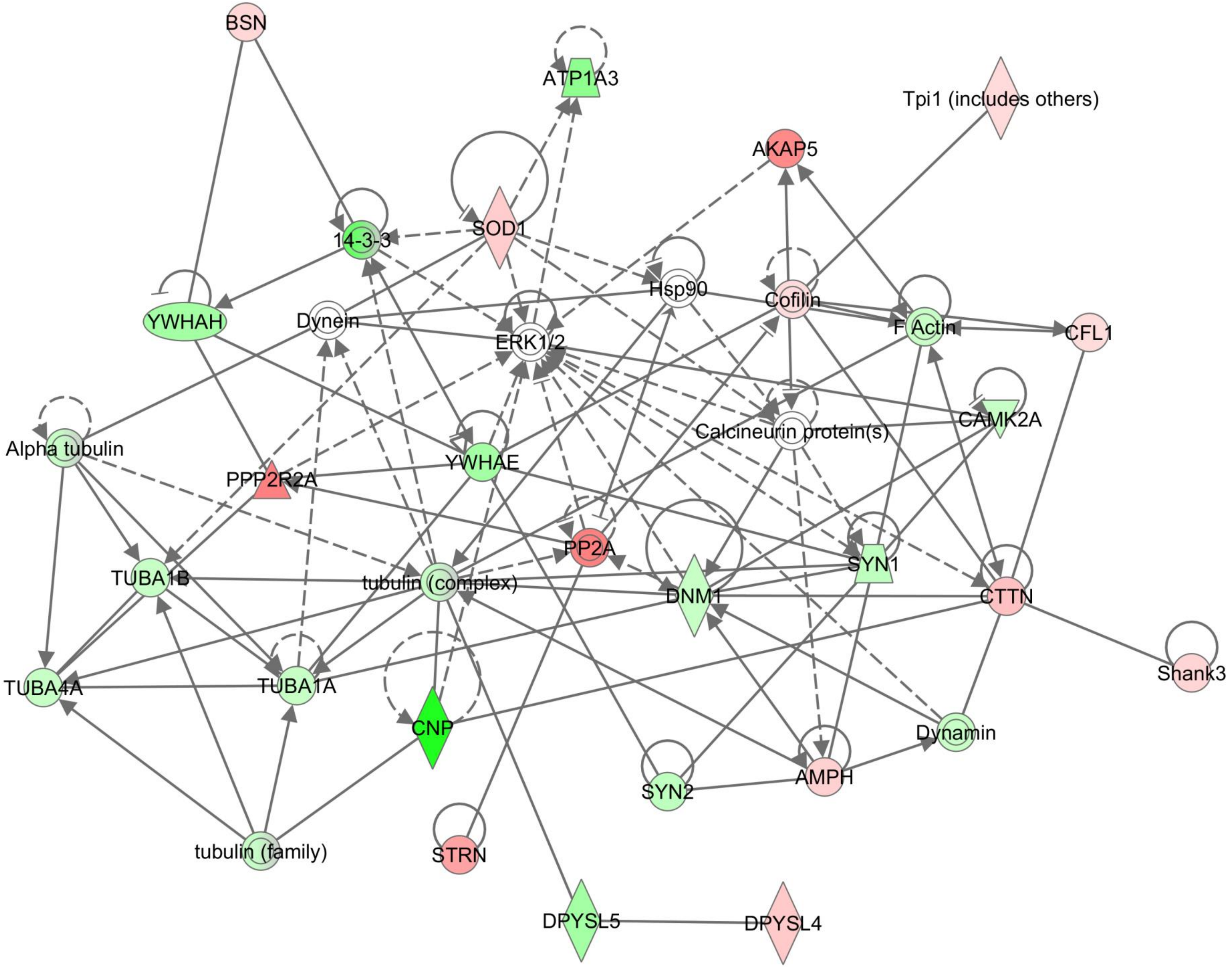
BSN
ATP1A3
Tpi1 (includes others)
AKAP5
SOD1
14-3-3
Hsp90
Cofilin
YWHAH
Dynein
ERK1/2
F Actin
CFL1
CAMK2A
Calcineurin protein(s)
Alpha tubulin
YWHAE
PPP2R2A
PP2A
SYN1
TUBA1B
tubulin (complex)
DNM1
CTTN
Shank3
TUBA4A
TUBA1A
CNP
Dynamin
AMPH
SYN2
tubulin (family)
STRN
DPYSL5
DPYSL4

Network (B)

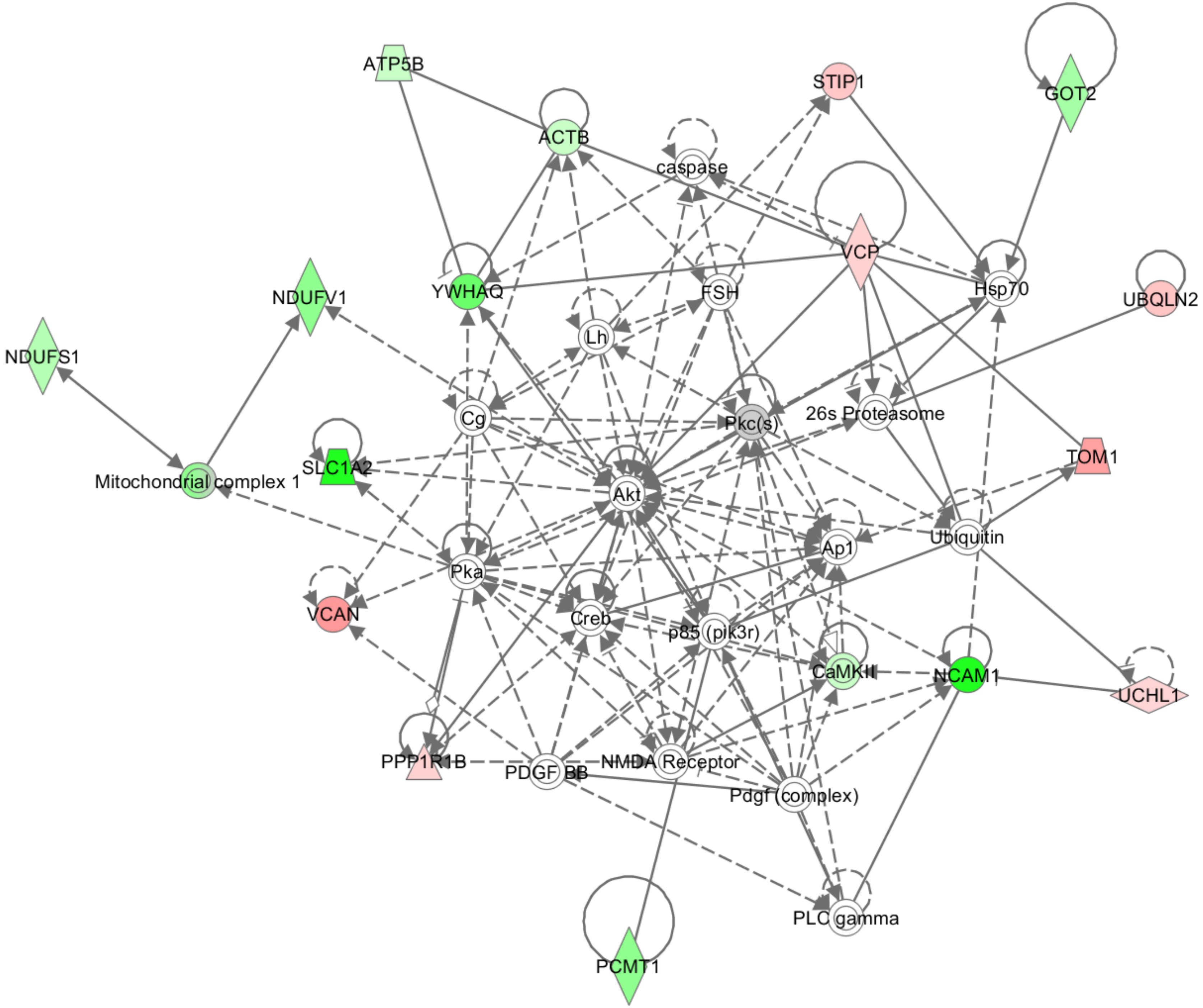
ATP5B
ACTB
caspase
STIP1
GOT2
VCP
YWHAQ
FSH
Hsp70
UBQLN2
NDUFV1
NDUFS1
Lh
Cg
Pkc(s)
26s Proteasome
TOM1
SLC1A2
Mitochondrial complex 1
Akt
Ap1
Ubiquitin
Pka
VCAN
Creb
p85 (pik3r)
CaMKII
NCAM1
UCHL1
PPP1R1B
PDGF BB
NMDA Receptor
Pdgf (complex)
PLC gamma
PCMT1

Network (C)

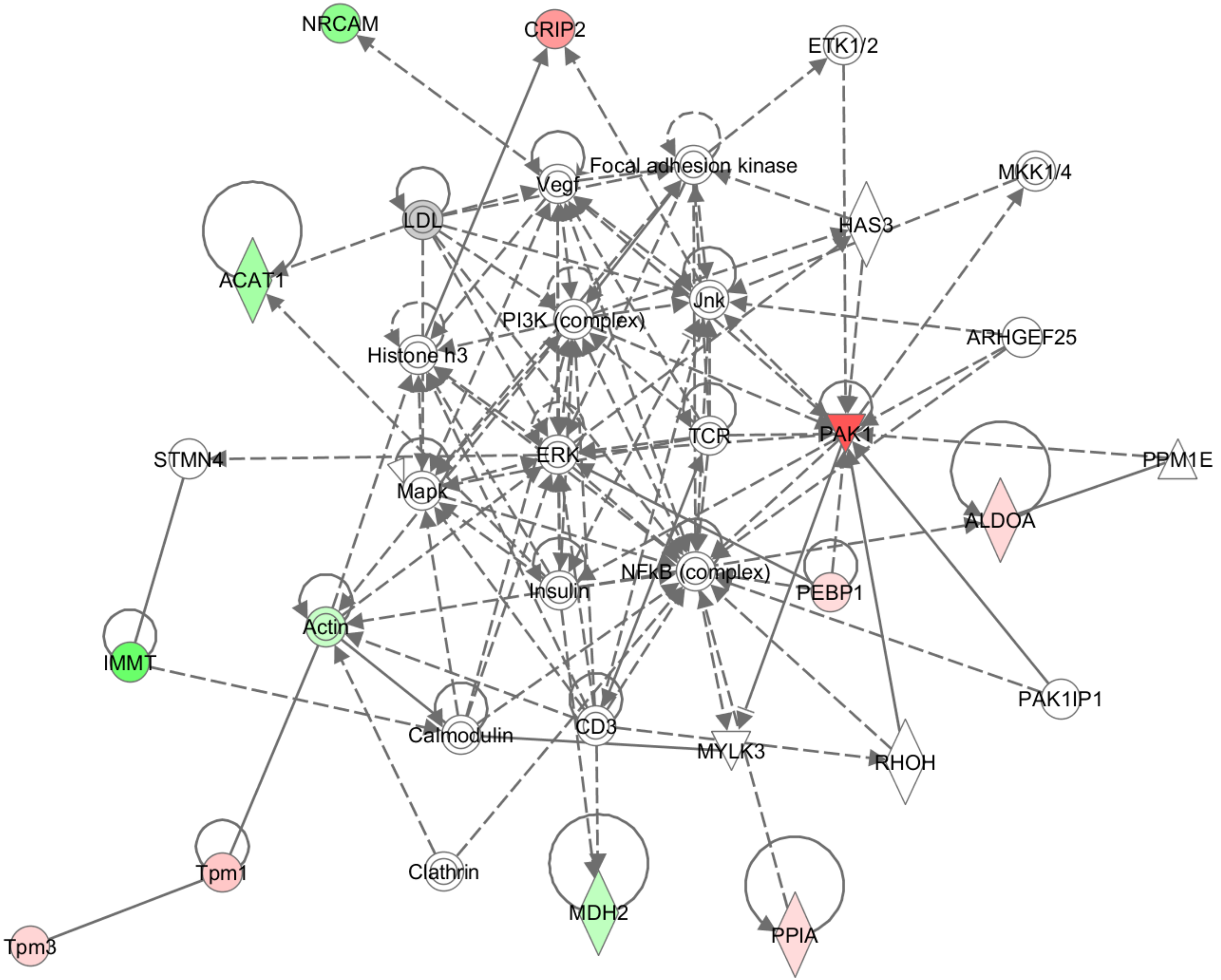
NRCAM
CRIP2
ETK1/2
Focal adhesion kinase
Vegf
MKK1/4
LDL
HAS3
ACAT1
Jnk
PI3K (complex)
ARHGEF25
Histone h3
TCR
PAK1
STMN4
ERK
PPM1E
Mapk
ALDOA
NFkB (complex)
PEBP1
Insulin
Actin
IMMT
PAK1IP1
CD3
Calmodulin
MYLK3
RHOH
Tpm1
Clathrin
MDH2
PPIA
Tpm3
