# Supplementary material for: Proteomics Analysis of Dorsal Striatum Reveals Changes in Synaptosomal Proteins following Methamphetamine Self-Administration in Rats
Source: PLoS One. 2015 Oct 20;10(10):e0139829. doi: 10.1371/journal.pone.0139829 (PMC4618287; doi:10.1371/journal.pone.0139829)
Supplement: S1 Table — (PDF) [file pone.0139829.s004.pdf]

Supplementary Table 1. All proteins identified										
Identified protein	Accession number	Molecular weight	Quantitative valune (Normalized spectral count)		Fold Change (Meth/Control)	P-Value	Unique peptide number		Protein sequence coverage	
			Control	Meth			Control	Meth	Control	Meth
Tubulin beta-2A chain	TBB2A_RAT	50 kDa	471.38	426.28	0.9	0.068	27	28	71.0%	71.9%
ATP synthase subunit beta, mitochondrial	ATPB_RAT (+1)	56 kDa	500.46	384.81	0.8	< 0.00010	25	24	71.1%	71.1%
Spectrin alpha chain, non-erythrocytic 1	SPTN1_RAT	285 kDa	344.71	379.99	1.1	0.099	79	94	48.4%	55.5%
Fructose-bisphosphate aldolase A	ALDOA_RAT	39 kDa	240.88	332.73	1.4	< 0.00010	19	20	74.5%	73.1%
Dihydropyrimidinase-related protein 2	DPYL2_RAT	62 kDa	265.80	303.80	1.1	0.059	24	28	72.0%	75.5%
V-type proton ATPase subunit B, brain isoform	VATB2_RAT	57 kDa	248.15	303.80	1.2	0.0095	23	27	71.8%	75.9%
Actin, cytoplasmic 1	ACTB_RAT	42 kDa	232.58	192.89	0.8	0.029	19	18	65.6%	65.6%
Triosephosphate isomerase	TPIS_RAT	27 kDa	196.24	245.93	1.3	0.0099	15	15	83.1%	76.7%
Creatine kinase B-type	KCRB_RAT	43 kDa	245.04	219.89	0.9	0.13	19	20	71.4%	74.0%
Guanine nucleotide-binding protein G(I)/G(S)/G(T) subunit beta-1	GBB1_RAT	37 kDa	190.01	221.82	1.2	0.063	13	15	60.6%	64.1%
Tubulin alpha-1A chain	TBA1A_RAT	50 kDa	230.50	187.10	0.8	0.018	19	20	71.0%	73.6%

Gamma-enolase	ENOG_RAT	47 kDa	233.62	212.18	0.9	0.16	18	19	69.4%	73.0%
Brain acid soluble protein 1	BASP1_RAT	22 kDa	179.62	202.53	1.1	0.13	18	21	79.5%	79.5%
ATP synthase subunit alpha, mitochondrial	ATPA_RAT	60 kDa	190.01	168.78	0.9	0.14	18	20	44.1%	45.9%
Glyceraldehyde-3-phosphate dehydrogenase	G3P_RAT	36 kDa	201.43	168.78	0.8	0.049	14	15	52.9%	55.3%
Protein kinase C and casein kinase substrate in neurons protein 1	PACN1_RAT	50 kDa	158.86	165.88	1	0.37	18	17	57.6%	60.1%
Heat shock cognate 71 kDa protein	HSP7C_RAT	71 kDa	156.78	148.52	0.9	0.34	22	23	46.1%	44.0%
Pyruvate kinase PKM	sp|P11980|KPYM_RAT	58 kDa	158.86	152.38	1	0.38	23	26	58.8%	65.0%
Phosphatidylethanolamine-binding protein 1	PEBP1_RAT	21 kDa	118.36	156.24	1.3	0.013	10	15	82.4%	87.2%
Fructose-bisphosphate aldolase C	ALDOC_RAT	39 kDa	172.36	187.10	1.1	0.23	15	14	70.5%	64.5%
Cofilin-1	COF1_RAT	19 kDa	124.59	155.27	1.2	0.038	10	13	49.4%	62.7%
Alpha-synuclein	sp|P37377|SYUA_RAT	15 kDa	104.87	124.41	1.2	0.11	9	13	85.0%	85.0%
Malate dehydrogenase, mitochondrial	MDHM_RAT	36 kDa	133.94	98.37	0.7	0.011	12	12	50.6%	45.9%
Microtubule-associated protein	D3ZRX4_RAT	52 kDa	99.68	107.05	1.1	0.33	16	15	50.8%	51.2%
Peptidyl-prolyl cis-trans isomerase A	PPIA_RAT	18 kDa	101.75	130.20	1.3	0.035	9	8	66.5%	66.5%
60 kDa heat shock protein, mitochondrial	CH60_RAT	61 kDa	105.91	130.20	1.2	0.064	23	29	54.3%	66.3%
Synapsin-1	sp|P09951|SYN1_RAT	74 kDa	127.71	95.48	0.7	0.018	22	19	57.0%	48.4%
Alpha-enolase	ENOA_RAT (+2)	47 kDa	175.47	145.63	0.8	0.053	12	14	54.1%	62.9%

Myelin basic protein transcript variant 1	I7FKL4_RAT (+1)	22 kDa	49.84	45.33	0.9	0.36	8	8	45.1%	45.1%
Calcium/calmodulin-dependent protein kinase type II subunit alpha	KCC2A_RAT	54 kDa	125.63	95.48	0.8	0.024	12	12	38.3%	39.5%
Protein Atp6v1a	D4A133_RAT	68 kDa	113.17	116.70	1	0.43	18	19	49.9%	50.2%
Dynamin-1	sp|P21575|DYN1_RAT	97 kDa	123.56	95.48	0.8	0.033	27	27	40.3%	40.3%
Aconitate hydratase, mitochondrial	ACON_RAT	85 kDa	125.63	87.76	0.7	0.0055	23	20	47.1%	37.9%
Tubulin beta-3 chain	TBB3_RAT	50 kDa	348.87	317.30	0.9	0.12	10	11	68.2%	66.2%
RCG45400	G3V7C6_RAT	50 kDa	474.50	416.64	0.9	0.027	8	8	71.0%	71.0%
Rab GDP dissociation inhibitor alpha	GDIA_RAT	51 kDa	112.14	96.44	0.9	0.15	17	21	55.7%	62.2%
Ubiquitin carboxyl-terminal hydrolase isozyme L1	UCHL1_RAT	25 kDa	74.76	113.80	1.5	0.0027	10	10	70.9%	64.1%
14-3-3 protein zeta/delta	1433Z_RAT	28 kDa	104.87	94.52	0.9	0.25	10	11	43.7%	46.5%
Protein Tppp	D3ZQL7_RAT	24 kDa	70.60	96.44	1.4	0.027	8	10	33.9%	45.0%
14-3-3 protein gamma	1433G_RAT	28 kDa	82.03	71.37	0.9	0.22	12	11	64.0%	62.8%
cAMP-dependent protein kinase type II-beta regulatory subunit	KAP3_RAT	46 kDa	85.14	87.76	1	0.45	13	13	44.5%	44.5%
Protein LOC685778	D4A5G8_RAT (+1)	44 kDa	73.72	86.80	1.2	0.17	11	11	43.0%	43.0%
Microtubule-associated protein 6	sp|Q63560|MAP6_RAT	100 kDa	73.72	94.52	1.3	0.063	17	20	29.5%	33.9%
Guanine nucleotide-binding protein G(I)/G(S)/G(T) subunit beta-2	GBB2_RAT	37 kDa	150.55	170.70	1.1	0.14	8	9	55.0%	62.1%
Beta-synuclein	SYUB_RAT	15 kDa	102.79	102.23	1	0.51	4	5	46.7%	54.0%
Isoform 2 of Syntaxin-binding protein 1	sp|P61765-2|STXB1_RAT (+1)	69 kDa	94.48	79.08	0.8	0.14	21	20	49.1%	49.1%
Phosphoglycerate kinase 1	PGK1_RAT	45 kDa	86.18	88.73	1	0.45	14	15	55.6%	59.7%
Amphiphysin	AMPH_RAT (+1)	75 kDa	59.18	94.52	1.6	0.0027	15	20	32.9%	36.0%
Transitional endoplasmic reticulum ATPase	TERA_RAT	89 kDa	52.95	81.98	1.5	0.0078	18	21	38.1%	40.4%
Endophilin-A1 (Fragment)	F1LQ05_RAT (+1)	38 kDa	94.48	72.33	0.8	0.05	10	10	37.0%	47.6%

Superoxide dismutase [Cu-Zn]	Q6LDS4_RAT (+1)	16 kDa	45.69	81.98	1.8	0.00085	5	10	64.5%	80.9%
Succinate dehydrogenase [ubiquinone] flavoprotein subunit, mitochondrial	DHSA_RAT	72 kDa	57.11	62.69	1.1	0.34	12	20	31.2%	50.2%
Phosphoglycerate mutase 1	PGAM1_RAT	29 kDa	68.53	67.51	1	0.5	11	12	53.5%	64.2%
Septin 5, isoform CRA_c	D3ZT07_RAT (+3)	43 kDa	48.80	59.80	1.2	0.17	9	10	35.5%	42.5%
Isocitrate dehydrogenase [NAD] subunit beta, mitochondrial	IDH3B_RAT	42 kDa	57.11	56.90	1	0.53	8	8	31.7%	31.7%
Synapsin-2	G3V733_RAT (+1)	61 kDa	90.33	59.80	0.7	0.0076	12	12	44.1%	41.5%
Creatine kinase, mitochondrial 1, ubiquitous	Q5BJT9_RAT	47 kDa	80.99	63.65	0.8	0.086	11	11	41.1%	42.3%
Isocitrate dehydrogenase [NAD] subunit alpha, mitochondrial	F1LNF7_RAT (+1)	40 kDa	43.61	48.22	1.1	0.35	7	8	26.5%	31.7%
Neuromodulin	NEUM_RAT	24 kDa	43.61	45.33	1	0.47	10	9	61.9%	57.1%
Protein bassoon	G3V984_RAT	418 kDa	47.76	68.48	1.4	0.034	17	25	8.8%	12.4%
Dihydropyrimidinase-related protein 1	DPYL1_RAT	62 kDa	77.87	64.62	0.8	0.15	15	13	53.8%	51.9%
Isoform AMPH2-2 of Myc box-dependent-interacting protein 1	sp|O08839-2|BIN1_RAT (+1)	61 kDa	56.07	63.65	1.1	0.27	10	14	34.0%	41.8%
Septin 7	A2VCW8_RAT (+3)	51 kDa	50.88	61.72	1.2	0.18	7	12	25.2%	38.7%
Pyruvate dehydrogenase E1 component subunit beta, mitochondrial	ODPB_RAT	39 kDa	60.22	47.26	0.8	0.12	10	7	39.3%	34.0%
Tropomyosin alpha-3 chain	sp|Q63610|TPM3_RAT	29 kDa	41.53	61.72	1.5	0.029	11	15	47.6%	52.4%
ATP synthase subunit gamma, mitochondrial	ATPG_RAT (+2)	30 kDa	38.42	36.65	1	0.46	7	7	30.8%	30.8%
NADH dehydrogenase [ubiquinone] 1 alpha subcomplex subunit 10, mitochondrial	NDUAA_RAT	40 kDa	49.84	36.65	0.7	0.094	9	10	34.1%	33.0%
ATP synthase subunit d, mitochondrial	ATP5H_RAT	19 kDa	43.61	50.15	1.2	0.28	9	8	75.8%	71.4%
Isoform 1 of SH3 and multiple ankyrin repeat domains protein 3	sp|Q9JLU4-2|SHAN3_RAT (+1)	192 kDa	37.38	54.97	1.5	0.042	13	18	16.1%	21.7%
Homer protein homolog 1	sp|Q9Z214|HOME1_RAT	41 kDa	38.42	45.33	1.2	0.26	9	11	30.6%	35.0%
Adenylate kinase isoenzyme 1	KAD1_RAT	22 kDa	37.38	49.19	1.3	0.12	6	8	49.5%	50.0%
Glutamine synthetase	GLNA_RAT	42 kDa	41.53	45.33	1.1	0.38	9	10	39.7%	48.3%

Synaptosomal-associated protein 25	sp|P60881|SNP25_RAT	23 kDa	46.72	39.54	0.8	0.25	7	9	43.2%	55.8%
Peroxiredoxin-5, mitochondrial (Fragment)	D3ZEN5_RAT	17 kDa	37.38	34.72	0.9	0.42	8	6	71.4%	47.8%
NADH-ubiquinone oxidoreductase 75 kDa subunit, mitochondrial	NDUS1_RAT	79 kDa	52.95	32.79	0.6	0.019	15	11	38.5%	25.9%
Dynactin subunit 2	DCTN2_RAT	44 kDa	32.19	49.19	1.5	0.038	10	9	43.8%	36.8%
Elongation factor Tu, mitochondrial	EFTU_RAT	50 kDa	36.34	43.40	1.2	0.25	10	11	37.2%	43.4%
Succinyl-CoA ligase [ADP/GDP-forming] subunit alpha, mitochondrial	SUCA_RAT	36 kDa	44.65	35.68	0.8	0.19	5	5	23.1%	22.5%
Calcium/calmodulin-dependent protein kinase II, beta, isoform CRA_a	G3V9G3_RAT (+1)	60 kDa	85.14	75.23	0.9	0.24	6	7	32.3%	38.9%
Protein Tubb4a	B4F7C2_RAT	50 kDa	425.70	364.56	0.9	0.015	4	4	72.1%	69.4%
Elongation factor 1-alpha 1	EF1A1_RAT (+1)	50 kDa	35.30	26.04	0.7	0.15	10	12	40.3%	43.5%
Protein phosphatase 1 regulatory subunit 1B	PPR1B_RAT	23 kDa	27.00	44.36	1.6	0.026	6	6	48.8%	50.7%
Isocitrate dehydrogenase [NAD] subunit gamma 1, mitochondrial	IDHG1_RAT (+1)	43 kDa	48.80	41.47	0.8	0.25	8	9	33.6%	38.7%
Microtubule-associated protein	F1LNK0_RAT (+3)	202 kDa	36.34	39.54	1.1	0.4	14	18	14.5%	21.6%
Cytochrome b-c1 complex subunit 1, mitochondrial	QCR1_RAT	53 kDa	48.80	30.86	0.6	0.028	11	9	45.8%	36.3%
Rabphilin-3A	F1LPB9_RAT (+1)	76 kDa	35.30	40.51	1.1	0.32	8	10	21.8%	26.6%
Protein Sept6	B5DFG5_RAT	49 kDa	28.03	38.58	1.4	0.12	7	10	25.5%	32.3%
Calreticulin	CALR_RAT	48 kDa	34.26	37.61	1.1	0.39	11	9	48.3%	45.9%
Tropomodulin-2	TMOD2_RAT	39 kDa	30.11	43.40	1.4	0.076	5	7	26.2%	31.1%
Malate dehydrogenase, cytoplasmic	MDHC_RAT	36 kDa	38.42	25.08	0.7	0.06	7	7	30.8%	30.8%
Oxidation resistance protein 1	sp|Q4V8B0|OXR1_RAT	93 kDa	24.92	40.51	1.6	0.036	8	10	15.1%	19.9%
Sodium/potassium-transporting ATPase subunit alpha-3	AT1A3_RAT	112 kDa	49.84	19.29	0.4	0.00015	13	7	20.8%	14.3%
Endophilin-B2	D4A7V1_RAT (+1)	45 kDa	21.80	48.22	2.2	0.0011	3	11	13.9%	34.4%
NADH dehydrogenase [ubiquinone] flavoprotein 2, mitochondrial	NDUV2_RAT	27 kDa	25.96	25.08	1	0.51	6	6	37.9%	39.1%

Isoform Short of 14-3-3 protein beta/alpha	sp|P35213-2|1433B_RAT (+1)	28 kDa	43.61	38.58	0.9	0.33	7	7	46.7%	50.0%
10 kDa heat shock protein, mitochondrial	CH10_RAT	11 kDa	35.30	19.29	0.5	0.02	3	3	37.3%	37.3%
ATPase, H+ transporting, V1 subunit E isoform 1, isoform CRA_a	G3V7L8_RAT (+1)	26 kDa	35.30	23.15	0.7	0.071	6	5	26.1%	26.1%
Serine/threonine-protein phosphatase 2A catalytic subunit beta isoform	PP2AB_RAT	36 kDa	35.30	34.72	1	0.52	8	10	42.1%	53.1%
Stress-70 protein, mitochondrial	F1M953_RAT (+1)	74 kDa	38.42	27.00	0.7	0.098	10	10	21.2%	20.0%
Protein DJ-1	PARK7_RAT	20 kDa	19.73	24.11	1.2	0.31	5	8	38.1%	64.6%
Secernin-1	SCRN1_RAT	46 kDa	25.96	34.72	1.3	0.16	7	9	33.6%	35.7%
NADH dehydrogenase [ubiquinone] iron-sulfur protein 4, mitochondrial	NDUS4_RAT	20 kDa	14.54	18.32	1.3	0.31	4	6	25.7%	41.7%
Aspartate aminotransferase, cytoplasmic	AATC_RAT	46 kDa	43.61	21.22	0.5	0.0036	10	8	43.1%	35.8%
Protein phosphatase 1 regulatory subunit 7	PP1R7_RAT	41 kDa	28.03	38.58	1.4	0.12	6	11	23.6%	48.9%
ERC protein 2	sp|Q8K3M6|ERC2_RAT	111 kDa	21.80	31.83	1.5	0.11	8	10	13.9%	15.8%
Cytochrome c, somatic	CYC_RAT (+1)	12 kDa	29.07	27.97	1	0.49	5	6	41.9%	49.5%
ATP synthase subunit O, mitochondrial	ATPO_RAT	23 kDa	28.03	32.79	1.2	0.32	5	6	39.0%	39.9%
Protein Cttn	D3ZGE6_RAT (+1)	53 kDa	15.57	33.76	2.2	0.007	4	9	10.2%	22.0%
Tubulin beta-5 chain	sp|P69897|TBB5_RAT	50 kDa	422.58	374.20	0.9	0.044	4	4	71.2%	71.2%
Clathrin light chain B	sp|P08082|CLCB_RAT	25 kDa	21.80	32.79	1.5	0.089	3	4	19.7%	19.7%
Tubulin alpha-1B chain	TBA1B_RAT	50 kDa	242.96	192.89	0.8	0.0089	2	2	71.0%	73.4%
Protein LOC100912599	D3ZCZ9_RAT	13 kDa	25.96	28.93	1.1	0.4	6	6	54.3%	54.3%
Peroxiredoxin-2	PRDX2_RAT	22 kDa	30.11	27.00	0.9	0.39	6	6	41.4%	46.5%
Guanine nucleotide-binding protein G(o) subunit alpha	sp|P59215|GNAO_RAT	40 kDa	32.19	20.25	0.6	0.065	8	7	35.6%	32.2%
Isoform 2 of Dihydropyrimidinase-related protein 3	sp|Q62952-2|DPYL3_RAT (+1)	74 kDa	52.95	53.04	1	0.54	9	9	29.1%	30.0%
Complement component 1 Q subcomponent-binding protein, mitochondrial	C1QBP_RAT	31 kDa	25.96	28.93	1.1	0.4	5	5	29.0%	29.0%

Peptidylprolyl cis/trans isomerase, NIMA-interacting 1	B0BNL2_RAT	18 kDa	23.88	35.68	1.5	0.081	4	6	41.8%	59.4%
Coronin-1A	COR1A_RAT	51 kDa	17.65	22.18	1.3	0.29	6	6	26.0%	27.3%
Succinyl-CoA:3-ketoacid coenzyme A transferase 1, mitochondrial	SCOT1_RAT	56 kDa	20.77	18.32	0.9	0.41	6	6	21.9%	21.0%
Stathmin	STMN1_RAT	17 kDa	11.42	11.57	1	0.57	4	3	30.9%	24.8%
Tubulin alpha-4A chain	TBA4A_RAT	50 kDa	220.12	179.38	0.8	0.023	4	4	62.1%	63.4%
Stress-induced-phosphoprotein 1	R9PXW7_RAT (+1)	63 kDa	18.69	37.61	2	0.0082	7	13	20.4%	34.2%
N(G),N(G)-dimethylarginine dimethylaminohydrolase 1	DDAH1_RAT	31 kDa	22.84	27.97	1.2	0.28	8	8	61.1%	57.9%
Voltage-dependent anion-selective channel protein 1	VDAC1_RAT	31 kDa	17.65	17.36	1	0.55	5	6	26.5%	30.4%
Destrin	DEST_RAT	19 kDa	15.57	27.00	1.7	0.055	4	5	32.1%	38.8%
NADH dehydrogenase (Ubiquinone) Fe-S protein 5	B5DEL8_RAT	13 kDa	19.73	34.72	1.8	0.029	3	5	36.8%	57.5%
Adenylyl cyclase-associated protein 1	CAP1_RAT	52 kDa	20.77	21.22	1	0.53	7	6	27.6%	22.4%
Isoform 2 of Drebrin-like protein	sp|Q9JHL4-2|DBNL_RAT (+2)	48 kDa	27.00	28.93	1.1	0.45	6	7	21.5%	26.1%
Isoform 2 of Limbic system-associated membrane protein	sp|Q62813-2|LSAMP_RAT (+2)	40 kDa	24.92	18.32	0.7	0.2	4	5	17.2%	18.3%
Microtubule-associated protein 1B	F1LRL9_RAT	270 kDa	17.65	24.11	1.4	0.2	6	7	3.9%	4.5%
Protein disulfide-isomerase A3	PDIA3_RAT	57 kDa	21.80	13.50	0.6	0.11	7	8	16.8%	23.2%
Dihydropyrimidinase-related protein 4 (Fragment)	DPYL4_RAT (+1)	61 kDa	17.65	32.79	1.9	0.023	8	12	27.0%	38.3%
Guanine deaminase	Q9JKB7_RAT	51 kDa	23.88	13.50	0.6	0.061	12	8	41.4%	33.7%
Serine/threonine-protein phosphatase 2B catalytic subunit alpha isoform	sp|P63329|PP2BA_RAT	59 kDa	22.84	16.40	0.7	0.19	8	5	24.4%	18.2%
Complexin-2	CPLX2_RAT	15 kDa	20.77	23.15	1.1	0.42	5	6	40.3%	48.5%
Platelet-activating factor acetylhydrolase IB subunit alpha	LIS1_RAT	47 kDa	25.96	25.08	1	0.51	6	6	21.7%	22.7%

Acetyl-CoA acetyltransferase, mitochondrial	THIL_RAT	45 kDa	30.11	14.47	0.5	0.013	8	6	35.4%	27.8%
14-3-3 protein epsilon	1433E_RAT	29 kDa	31.15	16.40	0.5	0.022	4	4	32.5%	25.5%
Translationally-controlled tumor protein	TCTP_RAT	19 kDa	14.54	22.18	1.5	0.14	3	4	43.0%	50.6%
Drebrin	sp|Q07266|DREB_RAT	77 kDa	17.65	27.97	1.6	0.084	5	7	12.7%	19.7%
Isoform Glt-1A of Excitatory amino acid transporter 2	sp|P31596-2|EAA2_RAT (+1)	62 kDa	31.15	5.79	0.2	< 0.00010	7	3	14.6%	8.1%
Hebp1 protein	B4F7C7_RAT	21 kDa	19.73	19.29	1	0.54	5	7	47.4%	66.8%
Hyaluronan and proteoglycan link protein 1	sp|P03994|HPLN1_RAT	40 kDa	13.50	19.29	1.4	0.2	4	6	21.8%	29.7%
NSFL1 cofactor p47	NSF1C_RAT	41 kDa	16.61	20.25	1.2	0.33	6	6	24.3%	24.3%
Prostate leucine zipper variant 2	D0UFD0_RAT (+1)	24 kDa	15.57	23.15	1.5	0.15	5	7	33.0%	43.3%
Endophilin-A2	SH3G1_RAT	41 kDa	24.92	25.08	1	0.55	4	5	21.5%	26.4%
Protein LOC100911918 (Fragment)	M0R7M8_RAT (+1)	30 kDa	20.77	18.32	0.9	0.41	3	6	15.8%	33.2%
Uncharacterized protein	D4A269_RAT (+1)	14 kDa	7.27	17.36	2.4	0.033	3	4	46.0%	65.9%
NADH dehydrogenase (Ubiquinone) flavoprotein 1	Q5XIH3_RAT	51 kDa	21.80	7.72	0.4	0.0071	6	5	24.1%	22.6%
Protein kinase, cAMP-dependent, regulatory, type 2, alpha, isoform CRA_a	G3V8Q6_RAT (+1)	45 kDa	23.88	36.65	1.5	0.065	5	6	28.4%	30.7%
Glutamate dehydrogenase 1, mitochondrial	DHE3_RAT	61 kDa	17.65	11.57	0.7	0.17	7	5	21.5%	15.8%
Neural cell adhesion molecule L1	D3ZPC4_RAT	140 kDa	14.54	16.40	1.1	0.44	5	8	8.8%	15.6%
14-3-3 protein eta	1433F_RAT	28 kDa	29.07	14.47	0.5	0.019	3	4	23.6%	24.8%
Heat shock 70 kDa protein 4	F1LRV4_RAT (+1)	94 kDa	21.80	12.54	0.6	0.078	6	8	10.7%	18.2%
Peroxiredoxin-6	PRDX6_RAT	25 kDa	17.65	17.36	1	0.55	4	7	31.7%	54.9%
Tyrosine 3-monooxygenase	TY3H_RAT	56 kDa	17.65	12.54	0.7	0.23	6	5	23.7%	20.1%
Protein-L-isoaspartate(D-aspartate) O-methyltransferase	PIMT_RAT	25 kDa	22.84	8.68	0.4	0.0086	5	3	44.9%	28.6%
Ketimine reductase mu-crystallin	CRYM_RAT	34 kDa	18.69	14.47	0.8	0.29	6	4	28.1%	25.6%
Isoform 5 of Tropomyosin alpha-1 chain	sp|P04692-5|TPM1_RAT	28 kDa	27.00	54.01	2	0.0018	4	5	40.8%	47.3%
ADP/ATP translocase 1	ADT1_RAT (+1)	33 kDa	18.69	9.64	0.5	0.064	5	5	25.5%	23.8%

Serine/threonine-protein kinase PAK 1	PAK1_RAT	61 kDa	4.15	25.08	6	< 0.00010	2	7	6.1%	20.8%
LIM and SH3 domain protein 1	LASP1_RAT	30 kDa	11.42	14.47	1.3	0.35	3	3	14.8%	14.8%
Protein Ubqln2	D4AA63_RAT	67 kDa	12.46	26.04	2.1	0.021	4	7	10.3%	15.7%
S-formylglutathione hydrolase	ESTD_RAT	31 kDa	13.50	12.54	0.9	0.5	4	6	29.1%	50.0%
Heat shock protein HSP 90-alpha	HS90A_RAT	85 kDa	14.54	12.54	0.9	0.42	6	5	12.1%	9.7%
Nucleosome assembly protein 1-like 4	NP1L4_RAT	44 kDa	11.42	17.36	1.5	0.18	4	5	18.9%	20.2%
Aldehyde dehydrogenase family 5, subfamily A1	G3V945_RAT	56 kDa	16.61	11.57	0.7	0.22	8	5	24.5%	15.5%
Myristoylated alanine-rich C-kinase substrate	F1LMW7_RAT (+1)	30 kDa	12.46	17.36	1.4	0.24	4	5	34.4%	38.0%
Aspartate aminotransferase, mitochondrial	AATM_RAT	47 kDa	18.69	8.68	0.5	0.041	8	3	21.2%	10.0%
Transgelin-3	TAGL3_RAT	23 kDa	12.46	16.40	1.3	0.29	4	4	29.1%	30.2%
Prostaglandin E synthase 3 (Fragment)	R9PXR7_RAT (+1)	19 kDa	12.46	14.47	1.2	0.42	3	4	26.4%	35.8%
Fatty acid-binding protein, epidermal	FABP5_RAT	15 kDa	15.57	19.29	1.2	0.32	4	5	47.4%	63.0%
Profilin	D3ZDU5_RAT (+1)	16 kDa	12.46	9.64	0.8	0.35	3	2	19.6%	19.6%
Protein RGD1559864	D3ZB78_RAT (+1)	41 kDa	6.23	17.36	2.8	0.018	5	6	29.2%	37.2%
Dihydropyrimidinase-related protein 5	DPYL5_RAT (+1)	62 kDa	21.80	10.61	0.5	0.035	6	6	20.4%	24.6%
Peptidyl-prolyl cis-trans isomerase FKBP1A	FKB1A_RAT	12 kDa	16.61	13.50	0.8	0.35	6	5	41.7%	41.7%
Rho GDP-dissociation inhibitor 1	GDIR1_RAT	23 kDa	15.57	15.43	1	0.56	4	3	34.3%	22.1%
ES1 protein homolog, mitochondrial	ES1_RAT	28 kDa	13.50	9.64	0.7	0.28	2	3	15.0%	18.8%
Dihydrolipoyl dehydrogenase, mitochondrial	DLDH_RAT	54 kDa	21.80	7.72	0.4	0.0071	6	2	24.8%	10.2%
Fascin	FSCN1_RAT	54 kDa	13.50	8.68	0.6	0.21	7	3	23.3%	12.2%
Eukaryotic translation initiation factor 5A-1	IF5A1_RAT	17 kDa	15.57	15.43	1	0.56	4	4	35.1%	35.1%
RCG61894, isoform CRA_a	G3V6L8_RAT	86 kDa	6.23	20.25	3.3	0.0052	3	8	9.4%	23.1%
ATPase, H+ transporting, V1 subunit G isoform 2	Q8R2H0_RAT	14 kDa	12.46	12.54	1	0.57	3	3	44.1%	48.3%
Neurabin-1	NEB1_RAT	123 kDa	10.38	15.43	1.5	0.21	4	7	6.0%	10.6%
Coiled-coil-helix-coiled-coil-helix domain containing 3 (Predicted), isoform CRA_a	D3ZUX5_RAT	26 kDa	11.42	15.43	1.4	0.28	3	5	11.5%	29.1%

Serine/threonine-protein phosphatase 2A 55 kDa regulatory subunit B alpha isoform	2ABA_RAT	52 kDa	5.19	23.15	4.5	0.0005 5	3	7	9.4%	23.5%
EF-hand domain-containing protein D2	EFHD2_RAT	27 kDa	12.46	15.43	1.2	0.36	3	3	30.1%	30.1%
Actin, alpha cardiac muscle 1	ACTC_RAT (+1)	42 kDa	73.72	65.58	0.9	0.27	2	3	31.8%	31.8%
Synaptopodin	B1VKB4_RAT (+2)	97 kDa	11.42	15.43	1.4	0.28	5	6	8.8%	10.2%
4-nitrophenylphosphatase domain and non-neuronal SNAP25-like protein homolog 1 (C. elegans), isoform CRA_b	G3V728_RAT (+1)	33 kDa	8.31	11.57	1.4	0.31	5	4	38.7%	31.0%
AP2-associated protein kinase 1	AAK1_RAT (+1)	104 kDa	16.61	12.54	0.8	0.28	6	5	15.6%	12.7%
78 kDa glucose-regulated protein	GRP78_RAT	72 kDa	24.92	33.76	1.4	0.15	4	7	13.0%	16.8%
ATP synthase subunit delta, mitochondrial	G3V7Y3_RAT	18 kDa	11.42	11.57	1	0.57	3	2	37.5%	32.1%
Superoxide dismutase [Mn], mitochondrial	SODM_RAT	25 kDa	15.57	8.68	0.6	0.11	3	2	19.4%	13.1%
Protein Ranbp1	D4A2G9_RAT	24 kDa	9.34	17.36	1.9	0.088	2	5	16.7%	49.3%
Ubiquitin carboxyl-terminal hydrolase	D3ZVQ0_RAT	96 kDa	9.34	8.68	0.9	0.53	6	7	11.4%	15.9%
DnaJ (Hsp40) homolog, subfamily A, member 2	Q5M9H7_RAT	46 kDa	10.38	11.57	1.1	0.49	5	6	22.6%	20.6%
Isoform 2 of Disks large homolog 2	sp|Q63622-2|DLG2_RAT (+6)	96 kDa	15.57	12.54	0.8	0.35	4	4	7.3%	7.3%
F-box only protein 2	G3V774_RAT (+1)	34 kDa	13.50	12.54	0.9	0.5	4	3	17.2%	13.2%
Nucleosome assembly protein 1-like 1	G3V6H9_RAT (+1)	45 kDa	14.54	14.47	1	0.57	3	4	16.4%	24.6%
Isoform Crk-I of Adapter molecule crk	sp|Q63768-2|CRK_RAT (+1)	23 kDa	12.46	14.47	1.2	0.42	3	5	21.6%	37.7%
F-actin-capping protein subunit alpha-2	CAZA2_RAT	33 kDa	10.38	11.57	1.1	0.49	5	5	41.3%	31.1%
Dihydrolipoamide S-succinyltransferase (E2 component of 2-oxo-glutarate complex), isoform CRA_a	G3V6P2_RAT (+1)	49 kDa	10.38	6.75	0.7	0.26	3	1	9.3%	4.6%
Enoyl-CoA hydratase, mitochondrial	ECHM_RAT	32 kDa	16.61	11.57	0.7	0.22	3	3	17.6%	17.6%
Cysteine-rich protein 2	CRIP2_RAT	23 kDa	5.19	19.29	3.7	0.0035	2	4	26.0%	52.9%
Phosphatidylinositol transfer protein alpha isoform	PIPNA_RAT (+1)	32 kDa	12.46	10.61	0.9	0.43	2	3	13.3%	22.1%

Isoform 2 of SRC kinase signaling inhibitor 1	sp|Q9QXY2-2|SRCN1_RAT (+1)	127 kDa	11.42	13.50	1.2	0.42	4	5	6.2%	7.4%
Isoform 2 of Brain-specific angiogenesis inhibitor 1-associated protein 2	sp|Q6GMN2-2|BAIP2_RAT (+1)	58 kDa	12.46	4.82	0.4	0.053	4	3	12.8%	11.1%
Annexin A3	ANXA3_RAT (+1)	36 kDa	7.27	14.47	2	0.092	3	5	13.6%	21.6%
Dual specificity mitogen-activated protein kinase kinase 1	MP2K1_RAT	43 kDa	13.50	10.61	0.8	0.35	3	3	11.5%	12.2%
Astrocytic phosphoprotein PEA-15	PEA15_RAT	15 kDa	14.54	7.72	0.5	0.11	3	3	30.0%	30.0%
Thy-1 membrane glycoprotein	THY1_RAT	18 kDa	12.46	11.57	0.9	0.51	1	2	9.3%	18.0%
Cytochrome b-c1 complex subunit Rieske, mitochondrial	UCRI_RAT	29 kDa	11.42	3.86	0.3	0.044	3	2	19.0%	15.7%
Sodium/potassium-transporting ATPase subunit beta-1	AT1B1_RAT	35 kDa	10.38	6.75	0.7	0.26	2	3	7.9%	12.2%
Mitochondrial inner membrane protein (Fragment)	IMMT_RAT	67 kDa	15.57	4.82	0.3	0.014	4	3	12.2%	8.1%
Neuronal cell adhesion molecule long isoform Nc17	Q6PW34_RAT (+7)	143 kDa	14.54	5.79	0.4	0.041	6	4	12.0%	6.9%
SH3-containing GRB2-like protein 3-interacting protein 1	SGIP1_RAT	89 kDa	6.23	12.54	2	0.11	3	5	8.3%	14.8%
GTP-binding nuclear protein Ran	RAN_RAT	24 kDa	8.31	7.72	0.9	0.54	2	3	12.5%	17.6%
Thioredoxin (Fragment)	R4GNK3_RAT (+1)	12 kDa	4.15	3.86	0.9	0.6	1	1	12.5%	12.5%
Elongation factor 1-alpha 2	EF1A2_RAT	50 kDa	19.73	16.40	0.8	0.35	2	3	22.5%	27.4%
Neurocan core protein	G3V8R2_RAT (+1)	136 kDa	6.23	14.47	2.3	0.056	2	3	2.9%	3.1%
Dihydrolipoyllysine-residue acetyltransferase component of pyruvate dehydrogenase complex, mitochondrial	ODP2_RAT	67 kDa	9.34	7.72	0.8	0.44	5	5	14.9%	14.9%
Glia maturation factor beta	GMFB_RAT (+1)	17 kDa	9.34	5.79	0.6	0.25	3	4	43.0%	37.3%
Mitochondrial import receptor subunit TOM70	R9PXR4_RAT (+1)	62 kDa	7.27	4.82	0.7	0.34	4	3	13.9%	7.7%
Obg-like ATPase 1	OLA1_RAT	45 kDa	9.34	8.68	0.9	0.53	3	3	15.2%	9.9%
Tumor protein D54	TPD54_RAT	24 kDa	5.19	11.57	2.2	0.095	2	4	15.9%	26.8%

Isoform 3 of Neuroplastin	sp|P97546-3|NPTN_RAT (+1)	31 kDa	14.54	8.68	0.6	0.16	3	1	16.6%	6.5%
Neuronal-specific septin-3	D3ZPP8_RAT (+3)	39 kDa	10.38	6.75	0.7	0.26	4	3	19.1%	18.8%
Myl6 protein	B2GV99_RAT (+1)	17 kDa	8.31	6.75	0.8	0.44	3	3	27.0%	30.9%
Synaptophysin	SYPH_RAT	33 kDa	9.34	6.75	0.7	0.35	2	3	7.5%	12.1%
Coiled-coil-helix-coiled-coil-helix domain-containing protein 6, mitochondrial	CHCH6_RAT	29 kDa	6.23	10.61	1.7	0.21	3	4	15.3%	19.2%
Ras-related protein Rab-3A	RAB3A_RAT	25 kDa	8.31	5.79	0.7	0.34	4	3	26.4%	21.4%
NADH dehydrogenase (Ubiquinone) 1 beta subcomplex, 9	B2RYW3_RAT	22 kDa	6.23	10.61	1.7	0.21	3	3	33.5%	37.4%
Transcriptional activator protein Pur-alpha	F1LPS8_RAT	34 kDa	10.38	8.68	0.8	0.44	3	3	25.4%	25.4%
2',3'-cyclic-nucleotide 3'-phosphodiesterase	CN37_RAT	47 kDa	12.46	2.89	0.2	0.012	4	2	15.7%	8.6%
26S protease regulatory subunit 6B	PRS6B_RAT	47 kDa	9.34	9.64	1	0.56	2	4	8.9%	23.0%
Isoform 2 of Septin-11	sp|B3GNI6-2|SEP11_RAT (+2)	50 kDa	20.77	17.36	0.8	0.35	2	3	18.1%	21.1%
Hydroxyacyl glutathione hydrolase	F1LQI1_RAT	34 kDa	5.19	8.68	1.7	0.25	3	2	15.5%	12.6%
Protein RGD1304884	D4A3C2_RAT	46 kDa	8.31	7.72	0.9	0.54	2	3	6.6%	12.5%
Hsp90 co-chaperone Cdc37	CDC37_RAT	45 kDa	7.27	10.61	1.5	0.29	2	2	10.0%	10.0%
ATPase inhibitor, mitochondrial	ATIF1_RAT	12 kDa	0.00	7.72	INF	0.0052	0	4	0.0%	15.0%
Protein phosphatase 1, regulatory subunit 9B	B1H262_RAT (+1)	90 kDa	7.27	5.79	0.8	0.45	2	3	5.0%	7.2%
Clathrin heavy chain 1	CLH1_RAT (+1)	192 kDa	9.34	4.82	0.5	0.17	6	3	7.1%	3.8%
Clusterin	CLUS_RAT (+2)	51 kDa	7.27	3.86	0.5	0.23	3	3	11.0%	11.0%
UMP-CMP kinase	KCY_RAT	22 kDa	8.31	8.68	1	0.56	3	3	23.5%	23.0%
Myosin regulatory light chain 12B	ML12B_RAT (+2)	20 kDa	6.23	8.68	1.4	0.36	2	3	16.9%	28.5%
Wiskott-Aldrich syndrome protein family member 1	WASF1_RAT	62 kDa	5.19	6.75	1.3	0.44	2	4	7.0%	13.4%
Isoform 2 of Basigin	sp|P26453-2|BASI_RAT (+1)	30 kDa	6.23	8.68	1.4	0.36	2	2	10.3%	10.3%
Sept4 protein	A0JN02_RAT (+4)	41 kDa	5.19	8.68	1.7	0.25	1	3	3.9%	14.1%
Phosphatase and actin regulator 1	PHAR1_RAT	66 kDa	5.19	6.75	1.3	0.44	3	3	8.3%	7.9%

Protein Ppa1	F7EPH4_RAT (+1)	33 kDa	6.23	10.61	1.7	0.21	3	4	20.1%	27.7%
14-3-3 protein theta	1433T_RAT	28 kDa	18.69	4.82	0.3	0.0032	4	3	27.3%	19.6%
Erythrocyte protein band 4.1-like 3, isoform CRA_b	G3V874_RAT (+2)	107 kDa	9.34	6.75	0.7	0.35	4	3	6.8%	5.1%
Aldehyde dehydrogenase X, mitochondrial	AL1B1_RAT (+1)	58 kDa	4.15	9.64	2.3	0.11	1	5	2.7%	16.0%
Reticulocalbin-2	RCN2_RAT	37 kDa	5.19	10.61	2	0.13	2	5	13.1%	25.9%
26S protease regulatory subunit 6A	PRS6A_RAT (+1)	49 kDa	9.34	3.86	0.4	0.11	5	3	17.3%	11.4%
Septin-2	SEPT2_RAT	42 kDa	6.23	5.79	0.9	0.56	3	4	14.4%	20.8%
Elongation factor Ts, mitochondrial	EFTS_RAT	35 kDa	6.23	7.72	1.2	0.45	3	3	22.5%	23.5%
Clathrin coat assembly protein AP180	F1LRK0_RAT	94 kDa	6.23	6.75	1.1	0.55	3	3	5.3%	7.9%
Protein Omg	F7EYB9_RAT (+2)	50 kDa	2.08	2.89	1.4	0.53	2	2	4.5%	8.6%
Sept9 protein (Fragment)	B2GVB4_RAT	62 kDa	4.15	2.89	0.7	0.46	1	1	3.3%	3.3%
Single-stranded DNA-binding protein	G3V7K6_RAT (+1)	17 kDa	7.27	8.68	1.2	0.46	2	2	22.3%	22.3%
Lactoylglutathione lyase	LGUL_RAT	21 kDa	9.34	3.86	0.4	0.11	2	2	16.3%	16.3%
Protein Tom1	Q5XI21_RAT	54 kDa	3.11	10.61	3.4	0.039	2	4	5.9%	16.9%
Mitochondrial fission regulator 1-like	MFR1L_RAT	32 kDa	4.15	4.82	1.2	0.55	3	3	19.4%	19.4%
Isoform 2 of Cell adhesion molecule 2	sp|Q1WIM2-2|CADM2_RAT (+1)	44 kDa	6.23	5.79	0.9	0.56	3	3	17.2%	20.0%
Microtubule-associated protein RP/EB family member 3	MARE3_RAT	32 kDa	10.38	15.43	1.5	0.21	2	4	15.3%	30.2%
Opioid-binding protein/cell adhesion molecule	F1M2I5_RAT (+2)	23 kDa	8.31	6.75	0.8	0.44	2	3	18.8%	20.8%
Profilin-1	PROF1_RAT	15 kDa	5.19	10.61	2	0.13	2	3	21.4%	31.4%
UV excision repair protein RAD23 homolog B	RD23B_RAT	43 kDa	5.19	6.75	1.3	0.44	2	2	7.2%	7.2%
GRIP1-associated protein 1	D3ZGL0_RAT (+2)	91 kDa	7.27	8.68	1.2	0.46	3	5	9.0%	16.3%
Alpha-adducin	D3ZZ99_RAT (+2)	70 kDa	4.15	5.79	1.4	0.42	2	3	4.0%	8.2%
Glycine cleavage system H protein, mitochondrial	GCSH_RAT	18 kDa	6.23	6.75	1.1	0.55	2	3	29.4%	35.3%
Phosphohistidine phosphatase 1 (Predicted), isoform CRA_a	D3ZP47_RAT	14 kDa	5.19	7.72	1.5	0.34	2	2	21.8%	21.8%
Protein Psd3	D3ZFY7_RAT (+6)	112 kDa	5.19	4.82	0.9	0.58	1	2	1.8%	3.9%

Isoform V3 of Versican core protein	sp|Q9ERB4-2|CSPG2_RAT (+2)	74 kDa	3.11	11.57	3.7	0.025	1	6	4.1%	15.6%
Cofilin 2, muscle (Predicted), isoform CRA_b	M0RC65_RAT	19 kDa	36.34	51.12	1.4	0.07	1	1	28.9%	30.7%
Beta-adducin	F8WFS9_RAT (+2)	81 kDa	3.11	5.79	1.9	0.29	2	3	5.7%	8.6%
Prohibitin	PHB_RAT	30 kDa	10.38	1.93	0.2	0.014	3	2	22.4%	17.3%
A-kinase anchor protein 5	AKAP5_RAT (+1)	76 kDa	2.08	8.68	4.2	0.043	2	2	5.0%	5.3%
L-lactate dehydrogenase B chain	LDHB_RAT	37 kDa	6.23	1.93	0.3	0.12	4	1	15.6%	3.6%
Coactosin-like protein	COTL1_RAT	16 kDa	8.31	3.86	0.5	0.16	2	1	19.7%	11.3%
ADP-ribosylation factor GTPase activating protein 1 heart isoform	Q3S4A4_RAT (+5)	47 kDa	6.23	6.75	1.1	0.55	1	3	5.9%	20.0%
Toll-interacting protein	TOLIP_RAT	30 kDa	0.00	7.72	INF	0.0052	0	3	0.0%	15.0%
Kinesin light chain 1	D3ZHG2_RAT (+3)	62 kDa	4.15	4.82	1.2	0.55	3	3	9.6%	11.6%
Isoform B of Calcium/calmodulin-dependent protein kinase type II subunit gamma	sp|P11730-2|KCC2G_RAT (+2)	58 kDa	33.23	31.83	1	0.48	3	3	19.5%	25.0%
Protein Kbtbd11	D4A5J1_RAT	68 kDa	1.04	5.79	5.6	0.075	1	5	3.5%	16.7%
WD repeat-containing protein 1	WDR1_RAT	66 kDa	4.15	5.79	1.4	0.42	1	5	3.6%	18.6%
ATPase, H+ transporting, V1 subunit D, isoform CRA_c	Q6P503_RAT	28 kDa	8.31	2.89	0.3	0.091	2	2	11.3%	11.3%
Calmodulin	CALM_RAT (+1)	17 kDa	4.15	7.72	1.9	0.23	1	2	11.4%	26.2%
Fatty acid-binding protein, heart	FABPH_RAT	15 kDa	4.15	6.75	1.6	0.32	1	2	10.5%	21.1%
Protein Ppidl1	M0RB67_RAT (+1)	41 kDa	2.08	4.82	2.3	0.26	1	4	8.4%	18.9%
Protein IMPACT	IMPCT_RAT	36 kDa	5.19	5.79	1.1	0.55	1	3	4.1%	12.9%
ADP/ATP translocase 2	ADT2_RAT	33 kDa	13.50	10.61	0.8	0.35	1	2	20.8%	25.8%
Nucleoside diphosphate kinase A	NDKA_RAT	17 kDa	11.42	0.00	0	0.00032	4	0	42.8%	0.0%
Protein kinase C beta type (Fragment)	F1LS36_RAT (+3)	69 kDa	8.31	3.86	0.5	0.16	3	3	9.3%	6.1%
Cytosolic non-specific dipeptidase	CNDP2_RAT	53 kDa	2.08	6.75	3.3	0.11	2	3	8.0%	12.2%
Alcohol dehydrogenase [NADP(+)]	AK1A1_RAT	37 kDa	2.08	5.79	2.8	0.17	1	4	5.9%	26.2%
DnaJ homolog subfamily A member 1	DNJA1_RAT	45 kDa	4.15	6.75	1.6	0.32	1	3	6.3%	15.6%
Gamma-synuclein	F1LQ96_RAT (+1)	13 kDa	3.11	2.89	0.9	0.62	1	2	11.5%	29.5%

Isoform 2 of AP-2 complex subunit beta	sp|P62944-2|AP2B1_RAT (+2)	106 kDa	4.15	6.75	1.6	0.32	2	1	6.4%	4.2%
Protein Wdr37	D3ZQ02_RAT	50 kDa	2.08	6.75	3.3	0.11	1	4	2.7%	18.7%
Succinate dehydrogenase [ubiquinone] iron-sulfur subunit, mitochondrial	DHSB_RAT	32 kDa	2.08	4.82	2.3	0.26	1	2	3.9%	8.9%
Cathepsin B	CATB_RAT (+1)	37 kDa	6.23	4.82	0.8	0.45	2	1	9.1%	5.3%
Protein RGD1561252	D4ABI6_RAT (+1)	26 kDa	5.19	3.86	0.7	0.45	1	2	7.0%	13.9%
Protein unc-45 homolog A	M0RC57_RAT	48 kDa	3.11	8.68	2.8	0.092	1	2	2.7%	5.7%
Ubiquitin thioesterase OTUB1	OTUB1_RAT	31 kDa	7.27	5.79	0.8	0.45	1	2	5.5%	7.0%
Eukaryotic translation initiation factor 4H	IF4H_RAT	27 kDa	3.11	5.79	1.9	0.29	2	4	11.7%	18.5%
Neural cell adhesion molecule 1 (Fragment)	F1LNY3_RAT (+3)	93 kDa	9.34	1.93	0.2	0.025	4	2	6.9%	2.7%
Isoform 2 of Protein piccolo	sp|Q9JKS6-2|PCLO_RAT (+2)	530 kDa	4.15	1.93	0.5	0.31	2	2	1.2%	1.1%
Transcriptional activator protein Pur-beta	PURB_RAT	33 kDa	6.23	2.89	0.5	0.22	2	3	20.0%	25.4%
Calcium/calmodulin-dependent protein kinase type II subunit delta	F1LWF6_RAT (+7)	58 kDa	30.11	29.90	1	0.54	2	2	15.7%	15.7%
Pyridoxal kinase	G3V647_RAT (+1)	35 kDa	1.04	6.75	6.5	0.044	1	4	3.2%	26.9%
Isoform 2 of Cortactin-binding protein 2	sp|Q2IBD4-2|CTTB2_RAT (+1)	67 kDa	5.19	4.82	0.9	0.58	3	1	9.2%	1.9%
Protein Stam	B5DF55_RAT	60 kDa	2.08	6.75	3.3	0.11	1	3	2.2%	9.0%
Cytochrome c oxidase subunit 5B, mitochondrial	COX5B_RAT	14 kDa	4.15	3.86	0.9	0.6	1	2	14.0%	27.9%
Heat shock protein 105 kDa	HS105_RAT	96 kDa	4.15	8.68	2.1	0.16	3	3	7.0%	7.2%
Alpha-internexin	AINX_RAT (+1)	56 kDa	5.19	2.89	0.6	0.32	3	2	10.7%	5.9%
Cytochrome c oxidase subunit 5A, mitochondrial	COX5A_RAT	16 kDa	6.23	1.93	0.3	0.12	4	2	41.1%	28.1%
Guanylate kinase	Q71RR7_RAT	22 kDa	1.04	7.72	7.4	0.025	1	3	6.1%	25.3%
Polyadenylate-binding protein 1	PABP1_RAT	71 kDa	3.11	6.75	2.2	0.2	1	3	2.5%	8.7%
Charged multivesicular body protein 5	CHMP5_RAT	25 kDa	2.08	2.89	1.4	0.53	1	2	8.2%	17.8%
Disks large homolog 1	DLG1_RAT (+1)	101 kDa	8.31	10.61	1.3	0.38	1	2	3.4%	5.2%
Abi1 protein	A2VD09_RAT (+1)	52 kDa	2.08	5.79	2.8	0.17	1	2	3.7%	7.1%
Myelin proteolipid protein	MYPR_RAT (+2)	30 kDa	4.15	0.96	0.2	0.17	2	1	9.4%	4.7%

NAD-dependent protein deacylase sirtuin-5, mitochondrial	SIR5_RAT	34 kDa	0.00	7.72	INF	0.0052	0	4	0.0%	23.5%
Glutathione S-transferase omega-1	GSTO1_RAT (+1)	28 kDa	0.00	3.86	INF	0.072	0	2	0.0%	10.4%
Fumarylacetoacetate hydrolase domain-containing protein 2	FAHD2_RAT	35 kDa	2.08	3.86	1.9	0.38	1	3	4.8%	20.8%
Coronin	G3V940_RAT	54 kDa	3.11	3.86	1.2	0.54	2	2	15.1%	10.7%
T-complex protein 1 subunit gamma	TCPG_RAT	61 kDa	2.08	2.89	1.4	0.53	2	2	7.3%	6.2%
Electron transfer flavoprotein subunit alpha, mitochondrial	ETFA_RAT	35 kDa	2.08	2.89	1.4	0.53	2	2	12.9%	12.0%
Centrin 2, isoform CRA_a	G3V9W0_RAT	20 kDa	6.23	2.89	0.5	0.22	2	2	26.7%	26.7%
S-phase kinase-associated protein 1	SKP1_RAT	19 kDa	5.19	2.89	0.6	0.32	2	2	24.5%	24.5%
D-dopachrome decarboxylase	DOPD_RAT	13 kDa	3.11	1.93	0.6	0.47	1	2	12.7%	22.9%
Neuronal growth regulator 1	NEGR1_RAT	38 kDa	2.08	2.89	1.4	0.53	1	2	4.0%	11.8%
Syntaxin-1B	STX1B_RAT	33 kDa	1.04	3.86	3.7	0.21	1	2	5.2%	9.7%
Regulator of G-protein-signaling 7	D3ZWG2_RAT (+1)	52 kDa	3.11	4.82	1.5	0.4	1	4	5.0%	16.0%
Isoform 2 of Ermin	sp|Q5RJL0-2|ERMIN_RAT (+1)	30 kDa	3.11	4.82	1.5	0.4	1	3	7.1%	30.2%
Protein Rabl6	D3ZKQ4_RAT	80 kDa	3.11	5.79	1.9	0.29	1	2	1.5%	3.6%
Keratin, type I cytoskeletal 10	K1C10_RAT	57 kDa	5.19	0.00	0	0.026	2	0	5.3%	0.0%
Purine nucleoside phosphorylase (Fragment)	D3ZXK9_RAT (+1)	32 kDa	3.11	2.89	0.9	0.62	2	2	15.6%	15.6%
Heat shock 70 kDa protein 4L	B4F772_RAT	94 kDa	6.23	5.79	0.9	0.56	2	2	5.9%	5.9%
Guanine nucleotide-binding protein subunit beta-4	GBB4_RAT	37 kDa	36.34	38.58	1.1	0.44	2	2	34.4%	36.8%
Eukaryotic translation initiation factor 5	IF5_RAT	49 kDa	5.19	1.93	0.4	0.2	2	1	5.6%	3.5%
Protein disulfide-isomerase	PDIA1_RAT	57 kDa	1.04	5.79	5.6	0.075	1	4	3.1%	13.8%
Protein Ank2	F1LM42_RAT (+2)	434 kDa	4.15	0.96	0.2	0.17	3	1	1.6%	0.9%
CDC42 effector protein (Rho GTPase binding) 4 (Predicted), isoform CRA_a	B1WC33_RAT	38 kDa	2.08	5.79	2.8	0.17	1	3	4.6%	16.9%
Glutathione S-transferase pi	B6DYQ7_RAT (+1)	23 kDa	3.11	1.93	0.6	0.47	2	1	15.2%	7.6%
Synaptotagmin-1	SYT1_RAT	47 kDa	5.19	0.00	0	0.026	3	0	10.7%	0.0%

Keratin, type II cytoskeletal 1	K2C1_RAT	65 kDa	5.19	0.00	0	0.026	3	0	6.1%	0.0%
3-hydroxyisobutyrate dehydrogenase, mitochondrial	3HIDH_RAT	35 kDa	4.15	3.86	0.9	0.6	3	3	15.2%	15.2%
Cysteine and glycine-rich protein 1	CSRP1_RAT	21 kDa	2.08	3.86	1.9	0.38	2	3	17.6%	25.4%
Endoplasmic reticulum resident protein 29	ERP29_RAT	29 kDa	2.08	2.89	1.4	0.53	2	2	14.2%	14.2%
Eukaryotic translation initiation factor 4B	Q5RKG9_RAT	69 kDa	3.11	2.89	0.9	0.62	2	2	6.7%	6.7%
Septin 8 (Predicted)	G3V9Z6_RAT	50 kDa	3.11	1.93	0.6	0.47	2	1	10.5%	4.7%
Protein Chmp4bl1	D4A9Z8_RAT (+1)	25 kDa	2.08	4.82	2.3	0.26	1	2	7.1%	13.4%
Microtubule-associated protein 1 A, isoform CRA_c	G3V7U2_RAT (+1)	300 kDa	4.15	5.79	1.4	0.42	3	0	2.3%	0.4%
Acp1 protein	B0BNC1_RAT (+2)	18 kDa	2.08	1.93	0.9	0.66	1	1	7.6%	7.6%
Hsc70-interacting protein	F10A1_RAT	41 kDa	1.04	2.89	2.8	0.34	1	1	3.5%	3.5%
T-complex protein 1 subunit epsilon	TCPE_RAT	60 kDa	5.19	0.00	0	0.026	3	0	14.0%	0.0%
Protein kinase C and casein kinase substrate in neurons 2 protein	R9PXU3_RAT (+5)	52 kDa	1.04	5.79	5.6	0.075	1	3	5.8%	12.8%
Omega-amidase NIT2	NIT2_RAT	31 kDa	2.08	3.86	1.9	0.38	1	3	12.7%	31.2%
Isoform 2 of Microtubule-associated protein 4	sp|Q5M7W5-2|MAP4_RAT (+1)	103 kDa	1.04	5.79	5.6	0.075	1	2	1.7%	3.3%
Protein Rap1gap	F1LV89_RAT	81 kDa	4.15	3.86	0.9	0.6	1	2	2.3%	5.4%
Protein LOC683295 (Fragment)	F1M0B2_RAT (+1)	58 kDa	6.23	1.93	0.3	0.12	2	1	4.1%	2.2%
Carbonic anhydrase 2	CAH2_RAT	29 kDa	6.23	0.00	0	0.012	2	0	16.2%	0.0%
Serum albumin	ALBU_RAT	69 kDa	0.00	6.75	INF	0.01	0	3	0.0%	6.4%
Guanine nucleotide-binding protein subunit beta-5	GBB5_RAT	39 kDa	5.19	0.00	0	0.026	2	0	8.5%	0.0%
Vesicle-fusing ATPase (Fragment)	F1LQ81_RAT (+1)	83 kDa	3.11	0.96	0.3	0.29	2	1	3.6%	2.0%
CD166 antigen	CD166_RAT	65 kDa	2.08	1.93	0.9	0.66	1	1	5.0%	5.0%
Clathrin light chain A	sp|P08081|CLCA_RAT	27 kDa	1.04	0.00	0	0.48	1	0	13.7%	0.0%
Protein Setd7	D4ADE5_RAT	41 kDa	4.15	1.93	0.5	0.31	1	2	4.9%	12.3%
Kinesin heavy chain isoform 5A	F1M8F2_RAT (+1)	117 kDa	3.11	3.86	1.2	0.54	1	2	1.7%	2.9%
Major prion protein	PRIO_RAT	28 kDa	2.08	3.86	1.9	0.38	1	2	4.7%	19.7%

Isoform 2 of Elongation factor 1-delta	sp|Q68FR9-2|EF1D_RAT (+1)	72 kDa	1.04	4.82	4.6	0.13	1	2	1.9%	3.9%
Syntaphilin	SNPH_RAT	54 kDa	1.04	1.93	1.9	0.53	1	1	3.8%	3.8%
Protein Mrps36	M0R776_RAT	11 kDa	2.08	3.86	1.9	0.38	1	1	12.6%	12.6%
F-actin-capping protein subunit beta	CAPZB_RAT	31 kDa	4.15	1.93	0.5	0.31	1	1	5.2%	5.2%
MOB-like protein phocein	PHOCN_RAT	26 kDa	0.00	4.82	INF	0.037	0	3	0.0%	35.1%
Cytochrome c oxidase subunit 2	COX2_RAT	26 kDa	6.23	0.00	0	0.012	2	0	26.0%	0.0%
NADH dehydrogenase (Ubiquinone) flavoprotein 3-like, isoform CRA_a	G3V644_RAT (+1)	49 kDa	2.08	3.86	1.9	0.38	2	3	7.2%	12.0%
Protein LOC100911774	Q1RP74_RAT (+1)	27 kDa	3.11	2.89	0.9	0.62	2	3	16.8%	25.0%
NADH dehydrogenase (Ubiquinone) Fe-S protein 3 (Predicted), isoform CRA_c	D3ZG43_RAT	30 kDa	1.04	1.93	1.9	0.53	1	2	5.3%	10.2%
Actin-related protein 3	ARP3_RAT	47 kDa	4.15	1.93	0.5	0.31	2	2	9.8%	9.8%
C-terminal-binding protein 1	CTBP1_RAT (+1)	47 kDa	3.11	1.93	0.6	0.47	2	2	11.9%	11.9%
Isoform 2 of Prostaglandin reductase 2	sp|Q5BK81-2|PTGR2_RAT (+1)	30 kDa	2.08	2.89	1.4	0.53	2	2	16.0%	16.0%
Transcription elongation factor B polypeptide 2	ELOB_RAT	13 kDa	4.15	0.96	0.2	0.17	2	1	31.4%	31.4%
RIMS-binding protein 2	D4A2L1_RAT (+2)	119 kDa	3.11	1.93	0.6	0.47	2	1	4.3%	1.6%
Crk-like protein	CRKL_RAT	34 kDa	1.04	3.86	3.7	0.21	1	2	5.6%	10.9%
Citrate synthase, mitochondrial	CISY_RAT (+1)	52 kDa	1.04	2.89	2.8	0.34	1	2	3.4%	11.8%
Heat shock protein HSP 90-beta	HS90B_RAT	83 kDa	7.27	9.64	1.3	0.37	2	1	7.9%	6.2%
Isocitrate dehydrogenase [NADP] cytoplasmic	IDHC_RAT (+1)	47 kDa	4.15	0.96	0.2	0.17	3	1	10.1%	2.9%
Antisecretory factor	O88321_RAT (+1)	41 kDa	1.04	3.86	3.7	0.21	1	3	7.4%	15.8%
Protein kinase C	F1LMV8_RAT (+2)	83 kDa	2.08	2.89	1.4	0.53	1	2	3.4%	4.8%
Neurotrimin	G3V964_RAT (+1)	38 kDa	5.19	1.93	0.4	0.2	2	2	9.6%	11.3%
Fumarate hydratase 1	Q5M964_RAT (+2)	54 kDa	2.08	1.93	0.9	0.66	1	2	5.5%	11.2%
BWK4	Q5VLR5_RAT	47 kDa	1.04	3.86	3.7	0.21	1	2	5.7%	9.1%
Cytochrome b-c1 complex subunit 2, mitochondrial	QCR2_RAT	48 kDa	5.19	0.96	0.2	0.093	2	1	6.0%	3.5%
Cystatin-B	CYTB_RAT	11 kDa	1.04	0.96	0.9	0.73	1	1	24.5%	30.6%

Aldose reductase	ALDR_RAT	36 kDa	0.00	3.86	INF	0.072	0	2	0.0%	8.2%
Stomatin-like protein 2, mitochondrial	STML2_RAT	38 kDa	2.08	0.96	0.5	0.47	2	1	13.0%	4.8%
Endophilin-A3 (Fragment)	F1M8F8_RAT (+2)	38 kDa	1.04	3.86	3.7	0.21	1	3	6.0%	16.0%
Protein Shisa7	D3ZPJ0_RAT	58 kDa	2.08	2.89	1.4	0.53	1	2	3.4%	6.3%
Protein Nebl	F1LVX3_RAT	31 kDa	2.08	2.89	1.4	0.53	2	1	13.3%	8.2%
Phosphate carrier protein, mitochondrial	G3V741_RAT (+1)	40 kDa	4.15	0.96	0.2	0.17	2	1	11.0%	7.6%
Thioredoxin-like protein 1	TXNL1_RAT	32 kDa	3.11	1.93	0.6	0.47	1	2	5.2%	16.3%
Glutaminase kidney isoform, mitochondrial	sp|P13264|GLSK_RAT	74 kDa	3.11	1.93	0.6	0.47	2	1	5.2%	2.5%
Isoform 2 of Protein NDRG2	sp|Q8VBU2-2|NDRG2_RAT (+1)	39 kDa	1.04	1.93	1.9	0.53	1	1	6.2%	8.7%
Protein Ndufb10	D4A0T0_RAT	21 kDa	3.11	0.96	0.3	0.29	1	1	8.0%	8.0%
Synaptosomal-associated protein	Q9JI56_RAT (+1)	29 kDa	2.08	1.93	0.9	0.66	1	1	4.3%	5.5%
Protein LOC100911422 (Fragment)	M0R9U5_RAT (+1)	38 kDa	2.08	1.93	0.9	0.66	1	1	4.6%	4.6%
Calretinin	CALB2_RAT	31 kDa	0.00	0.96	INF	0.52	0	1	0.0%	4.8%
Discs, large (Drosophila) homolog-associated protein 3, isoform CRA_b	G3V7T8_RAT	106 kDa	0.00	2.89	INF	0.14	0	2	0.0%	7.5%
SH3 and multiple ankyrin repeat domains protein 2	M0R5T5_RAT (+6)	162 kDa	0.00	1.93	INF	0.27	0	1	0.0%	1.0%
Inositol-trisphosphate 3-kinase A	IP3KA_RAT	51 kDa	0.00	1.93	INF	0.27	0	1	0.0%	5.2%
Protein kinase C gamma type	KPCG_RAT	78 kDa	0.00	4.82	INF	0.037	0	2	0.0%	4.2%
Protein LOC501282	M0R3V4_RAT	18 kDa	0.00	1.93	INF	0.27	0	1	0.0%	9.1%
Peroxiredoxin 3	G3V7I0_RAT (+1)	28 kDa	2.08	0.00	0	0.23	1	0	4.3%	0.0%
Cell cycle exit and neuronal differentiation protein 1	CEND_RAT (+1)	15 kDa	4.15	0.00	0	0.054	1	0	12.8%	0.0%
Isoform 2 of Growth factor receptor-bound protein 2	sp|P62994-2|GRB2_RAT (+1)	24 kDa	1.04	1.93	1.9	0.53	1	2	5.9%	10.3%
V-type proton ATPase subunit F	VATF_RAT	13 kDa	1.04	1.93	1.9	0.53	1	2	14.3%	34.5%
Protein Pgp	D3ZDK7_RAT	35 kDa	1.04	2.89	2.8	0.34	1	2	4.7%	9.7%
Ab2-417	Q7TMC7_RAT (+2)	107 kDa	2.08	0.96	0.5	0.47	2	1	4.3%	1.9%
Rab GTPase-binding effector protein 1	RABE1_RAT	99 kDa	2.08	1.93	0.9	0.66	1	2	2.2%	5.8%

Ndufa7 protein	A9UMV9_RAT	13 kDa	1.04	0.96	0.9	0.73	1	1	9.8%	9.8%
Protein Actbl2	D3ZRN3_RAT	42 kDa	22.84	30.86	1.4	0.17	1	1	11.7%	11.7%
Breast carcinoma-amplified sequence 1 homolog	F1LN49_RAT (+6)	62 kDa	1.04	0.00	0	0.48	1	0	2.7%	0.0%
Eukaryotic translation elongation factor 1 beta 2	B5DEN5_RAT	25 kDa	0.00	1.93	INF	0.27	0	1	0.0%	10.7%
Isoform 2 of Synaptosomal-associated protein 25	sp|P60881-2|SNP25_RAT	23 kDa	38.42	36.65	1	0.46	0	2	31.1%	62.1%
N(G),N(G)-dimethylarginine dimethylaminohydrolase 2	DDAH2_RAT	30 kDa	0.00	2.89	INF	0.14	0	2	0.0%	14.4%
Centaurin alpha	Q63629_RAT (+2)	48 kDa	0.00	1.93	INF	0.27	0	1	0.0%	4.5%
Dusp3 protein	B5DFF7_RAT (+1)	20 kDa	1.04	1.93	1.9	0.53	1	2	9.7%	18.9%
Platelet-activating factor acetylhydrolase IB subunit beta	PA1B2_RAT	26 kDa	2.08	0.96	0.5	0.47	2	1	12.2%	10.5%
Microtubule-associated proteins 1A/1B light chain 3A	MLP3A_RAT	14 kDa	1.04	0.96	0.9	0.73	1	1	11.6%	11.6%
CREB-regulated transcription coactivator 1	CRTC1_RAT	67 kDa	1.04	0.96	0.9	0.73	1	1	4.6%	4.6%
Glycogenin-1 (Fragment)	F8WFR6_RAT (+1)	37 kDa	0.00	0.96	INF	0.52	0	1	0.0%	10.9%
Neurofilament medium polypeptide	G3V7S2_RAT (+2)	94 kDa	0.00	1.93	INF	0.27	0	2	0.0%	4.6%
Coronin (Fragment)	F1LMV9_RAT	55 kDa	0.00	0.96	INF	0.52	0	1	0.0%	2.7%
Pdxp protein	B2GV79_RAT (+2)	31 kDa	0.00	1.93	INF	0.27	0	2	0.0%	12.7%
Protein RGD1310819 (Fragment)	D3ZBU7_RAT	119 kDa	0.00	1.93	INF	0.27	0	2	0.0%	4.8%
Protein Ankrd63	D3ZKY6_RAT	41 kDa	0.00	2.89	INF	0.14	0	2	0.0%	11.8%
Growth arrest-specific protein 7	GAS7_RAT (+2)	48 kDa	0.00	2.89	INF	0.14	0	2	0.0%	9.7%
Rab GDP dissociation inhibitor beta	GDIB_RAT	51 kDa	0.00	26.04	INF	< 0.00010	0	2	0.0%	15.5%
SH3 domain-containing kinase-binding protein 1 (Fragment)	M0RBZ7_RAT (+6)	47 kDa	0.00	2.89	INF	0.14	0	2	0.0%	8.2%
Pdhx protein (Fragment)	Q5BJX2_RAT (+1)	41 kDa	3.11	0.00	0	0.11	2	0	7.9%	0.0%
Apolipoprotein O-like	Q5U1W6_RAT	28 kDa	0.00	2.89	INF	0.14	0	2	0.0%	16.1%
Syntaxin 1A	Q9QXG3_RAT (+1)	33 kDa	2.08	0.00	0	0.23	2	0	15.6%	0.0%

RCG25732, isoform CRA_b	B5DEM5_RAT (+2)	24 kDa	0.00	0.96	INF	0.52	0	1	0.0%	5.5%
Regulating synaptic membrane exocytosis protein 1	F1LYS1_RAT (+2)	173 kDa	0.00	0.96	INF	0.52	0	1	0.0%	1.9%
Tyrosine-protein phosphatase non-receptor type 5	PTN5_RAT (+1)	42 kDa	0.00	0.96	INF	0.52	0	1	0.0%	10.6%
Protein Psmc6	G3V6W6_RAT (+1)	46 kDa	0.00	0.96	INF	0.52	0	1	0.0%	3.5%
Protein LOC100911453	M0R5M2_RAT (+2)	38 kDa	0.00	1.93	INF	0.27	0	1	0.0%	4.9%
1-phosphatidylinositol 4,5-bisphosphate phosphodiesterase beta-1 (Fragment)	F1M084_RAT (+2)	109 kDa	1.04	0.00	0	0.48	1	0	2.3%	0.0%
Dihydropteridine reductase	DHPR_RAT	26 kDa	2.08	0.00	0	0.23	2	0	15.4%	0.0%
Glutathione peroxidase 1	GPX1_RAT	22 kDa	0.00	1.93	INF	0.27	0	2	0.0%	23.4%
p55 protein	Q5BK33_RAT (+1)	51 kDa	0.00	1.93	INF	0.27	0	2	0.0%	5.8%
Anamorsin	CPIN1_RAT	33 kDa	0.00	0.96	INF	0.52	0	1	0.0%	12.0%
