# Supplementary material for: Proteomics Analysis of Dorsal Striatum Reveals Changes in Synaptosomal Proteins following Methamphetamine Self-Administration in Rats
Source: PLoS One. 2015 Oct 20;10(10):e0139829. doi: 10.1371/journal.pone.0139829 (PMC4618287; doi:10.1371/journal.pone.0139829)
Supplement: S2 Table — (PDF) [file pone.0139829.s005.pdf]

Supplementary Table 2. Differentially expressed proteins

Identified protein	Accession number	Molecular weight	Quantitative value (Normalized spectral count)		Fold Change (Meth/Control)	P-Value	Unique peptide number		Protein sequence coverage	
			Control	Meth			Control	Meth	Control	Meth
Nucleoside diphosphate kinase A	NDKA_RAT	17 kDa	11.42	0.00	−INF	0.00032	4	0	42.8%	0.0%
Carbonic anhydrase 2	CAH2_RAT	29 kDa	6.23	0.00	−INF	0.012	2	0	16.2%	0.0%
Cytochrome c oxidase subunit 2	COX2_RAT	26 kDa	6.23	0.00	−INF	0.012	2	0	26.0%	0.0%
Keratin, type I cytoskeletal 10	K1C10_RAT	57 kDa	5.19	0.00	−INF	0.026	2	0	5.3%	0.0%
Synaptotagmin-1	SYT1_RAT	47 kDa	5.19	0.00	−INF	0.026	3	0	10.7%	0.0%
Keratin, type II cytoskeletal 1	K2C1_RAT	65 kDa	5.19	0.00	−INF	0.026	3	0	6.1%	0.0%
T-complex protein 1 subunit epsilon	TCPE_RAT	60 kDa	5.19	0.00	−INF	0.026	3	0	14.0%	0.0%
Guanine nucleotide-binding protein subunit beta-5	GBB5_RAT	39 kDa	5.19	0.00	−INF	0.026	2	0	8.5%	0.0%
2',3'-cyclic-nucleotide 3'-phosphodiesterase	CN37_RAT	47 kDa	12.46	2.89	-5.0	0.012	4	2	15.7%	8.6%
Prohibitin	PHB_RAT	30 kDa	10.38	1.93	-5.0	0.014	3	2	22.4%	17.3%
Neural cell adhesion molecule 1 (Fragment)	F1LNY3_RAT (+3)	93 kDa	9.34	1.93	-5.0	0.025	4	2	6.9%	2.7%
Isoform Glt-1A of Excitatory amino acid transporter 2	sp|P31596-2|EAA2_RAT (+1)	62 kDa	31.15	5.79	-5.0	< 0.00010	7	3	14.6%	8.1%
14-3-3 protein theta	1433T_RAT	28 kDa	18.69	4.82	-3.3	0.0032	4	3	27.3%	19.6%
Mitochondrial inner membrane protein (Fragment)	IMMT_RAT	67 kDa	15.57	4.82	-3.3	0.014	4	3	12.2%	8.1%
Cytochrome b-c1 complex subunit Rieske, mitochondrial	UCRI_RAT	29 kDa	11.42	3.86	-3.3	0.044	3	2	19.0%	15.7%
Sodium/potassium-transporting ATPase subunit alpha-3	AT1A3_RAT	112 kDa	49.84	19.29	-2.5	0.00015	13	7	20.8%	14.3%
NADH dehydrogenase (Ubiquinone) flavoprotein 1	Q5XIH3_RAT	51 kDa	21.80	7.72	-2.5	0.0071	6	5	24.1%	22.6%
Dihydrolipoyl dehydrogenase, mitochondrial	DLDH_RAT	54 kDa	21.80	7.72	-2.5	0.0071	6	2	24.8%	10.2%
Protein-L-isoaspartate(D-aspartate) O-methyltransferase	PIMT_RAT	25 kDa	22.84	8.68	-2.5	0.0086	5	3	44.9%	28.6%
Neuronal cell adhesion molecule long isoform Nc17	Q6PW34_RAT (+7)	143 kDa	14.54	5.79	-2.5	0.041	6	4	12.0%	6.9%
Aspartate aminotransferase, cytoplasmic	AATC_RAT	46 kDa	43.61	21.22	-2.0	0.0036	10	8	43.1%	35.8%
Acetyl-CoA acetyltransferase, mitochondrial	THIL_RAT	45 kDa	30.11	14.47	-2.0	0.013	8	6	35.4%	27.8%
14-3-3 protein eta	1433F_RAT	28 kDa	29.07	14.47	-2.0	0.019	3	4	23.6%	24.8%
10 kDa heat shock protein, mitochondrial	CH10_RAT	11 kDa	35.30	19.29	-2.0	0.02	3	3	37.3%	37.3%
14-3-3 protein epsilon	1433E_RAT	29 kDa	31.15	16.40	-2.0	0.022	4	4	32.5%	25.5%
Dihydropyrimidinase-related protein 5	DPYL5_RAT (+1)	62 kDa	21.80	10.61	-2.0	0.035	6	6	20.4%	24.6%
Aspartate aminotransferase, mitochondrial	AATM_RAT	47 kDa	18.69	8.68	-2.0	0.041	8	3	21.2%	10.0%
NADH-ubiquinone oxidoreductase 75 kDa subunit, mitochondrial	NDUS1_RAT	79 kDa	52.95	32.79	-1.7	0.019	15	11	38.5%	25.9%
Cytochrome b-c1 complex subunit 1, mitochondrial	QCR1_RAT	53 kDa	48.80	30.86	-1.7	0.028	11	9	45.8%	36.3%
Aconitate hydratase, mitochondrial	ACON_RAT	85 kDa	125.63	87.76	-1.4	0.0055	23	20	47.1%	37.9%
Synapsin-2	G3V733_RAT (+1)	61 kDa	90.33	59.80	-1.4	0.0076	12	12	44.1%	41.5%
Malate dehydrogenase, mitochondrial	MDHM_RAT	36 kDa	133.94	98.37	-1.4	0.011	12	12	50.6%	45.9%
Synapsin-1	sp|P09951|SYN1_RAT	74 kDa	127.71	95.48	-1.4	0.018	22	19	57.0%	48.4%
Tubulin alpha-1B chain	TBA1B_RAT	50 kDa	242.96	192.89	-1.3	0.0089	2	2	71.0%	73.4%
Tubulin alpha-1A chain	TBA1A_RAT	50 kDa	230.50	187.10	-1.3	0.018	19	20	71.0%	73.6%
Tubulin alpha-4A chain	TBA4A_RAT	50 kDa	220.12	179.38	-1.3	0.023	4	4	62.1%	63.4%
Calcium/calmodulin-dependent protein kinase type II subunit alpha	KCC2A_RAT	54 kDa	125.63	95.48	-1.3	0.024	12	12	38.3%	39.5%
Actin, cytoplasmic 1	ACTB_RAT	42 kDa	232.58	192.89	-1.3	0.029	19	18	65.6%	65.6%
Dynamin-1	sp|P21575|DYN1_RAT	97 kDa	123.56	95.48	-1.3	0.033	27	27	40.3%	40.3%
Glyceraldehyde-3-phosphate dehydrogenase	G3P_RAT	36 kDa	201.43	168.78	-1.3	0.049	14	15	52.9%	55.3%
Endophilin-A1 (Fragment)	F1LQ05_RAT (+1)	38 kDa	94.48	72.33	-1.3	0.05	10	10	37.0%	47.6%
ATP synthase subunit beta, mitochondrial	ATPB_RAT (+1)	56 kDa	500.46	384.81	-1.3	< 0.00010	25	24	71.1%	71.1%
V-type proton ATPase subunit B, brain isoform	VATB2_RAT	57 kDa	248.15	303.80	1.2	0.0095	23	27	71.8%	75.9%
Cofilin-1	COF1_RAT	19 kDa	124.59	155.27	1.2	0.038	10	13	49.4%	62.7%
Triosephosphate isomerase	TPIS_RAT	27 kDa	196.24	245.93	1.3	0.0099	15	15	83.1%	76.7%
Phosphatidylethanolamine-binding protein 1	PEBP1_RAT	21 kDa	118.36	156.24	1.3	0.013	10	15	82.4%	87.2%
Peptidyl-prolyl cis-trans isomerase A	PPIA_RAT	18 kDa	101.75	130.20	1.3	0.035	9	8	66.5%	66.5%
Protein Tppp	D3ZQL7_RAT	24 kDa	70.60	96.44	1.4	0.027	8	10	33.9%	45.0%
Protein bassoon	G3V984_RAT	418 kDa	47.76	68.48	1.4	0.034	17	25	8.8%	12.4%
Fructose-bisphosphate aldolase A	ALDOA_RAT	39 kDa	240.88	332.73	1.4	< 0.00010	19	20	74.5%	73.1%
Ubiquitin carboxyl-terminal hydrolase isozyme L1	UCHL1_RAT	25 kDa	74.76	113.80	1.5	0.0027	10	10	70.9%	64.1%
Transitional endoplasmic reticulum ATPase	TERA_RAT	89 kDa	52.95	81.98	1.5	0.0078	18	21	38.1%	40.4%

Tropomyosin alpha-3 chain	sp|Q63610|TPM3_RAT	29 kDa	41.53	61.72	1.5	0.029	11	15	47.6%	52.4%
Dynactin subunit 2	DCTN2_RAT	44 kDa	32.19	49.19	1.5	0.038	10	9	43.8%	36.8%
Isoform 1 of SH3 and multiple ankyrin repeat domains protein 3	sp|Q9JLU4-2|SHAN3_RAT (+1)	192 kDa	37.38	54.97	1.5	0.042	13	18	16.1%	21.7%
Amphiphysin	AMPH_RAT (+1)	75 kDa	59.18	94.52	1.6	0.0027	15	20	32.9%	36.0%
Protein phosphatase 1 regulatory subunit 1B	PPR1B_RAT	23 kDa	27.00	44.36	1.6	0.026	6	6	48.8%	50.7%
Oxidation resistance protein 1	sp|Q4V8B0|OXR1_RAT	93 kDa	24.92	40.51	1.6	0.036	8	10	15.1%	19.9%
Superoxide dismutase [Cu-Zn]	Q6LDS4_RAT (+1)	16 kDa	45.69	81.98	1.8	0.00085	5	10	64.5%	80.9%
NADH dehydrogenase (Ubiquinone) Fe-S protein 5	B5DEL8_RAT	13 kDa	19.73	34.72	1.8	0.029	3	5	36.8%	57.5%
Dihydropyrimidinase-related protein 4 (Fragment)	DPYL4_RAT (+1)	61 kDa	17.65	32.79	1.9	0.023	8	12	27.0%	38.3%
Isoform 5 of Tropomyosin alpha-1 chain	sp|P04692-5|TPM1_RAT	28 kDa	27.00	54.01	2.0	0.0018	4	5	40.8%	47.3%
Stress-induced-phosphoprotein 1	R9PXW7_RAT (+1)	63 kDa	18.69	37.61	2.0	0.0082	7	13	20.4%	34.2%
Protein Ubqln2	D4AA63_RAT	67 kDa	12.46	26.04	2.1	0.021	4	7	10.3%	15.7%
Endophilin-B2	D4A7V1_RAT (+1)	45 kDa	21.80	48.22	2.2	0.0011	3	11	13.9%	34.4%
Protein Cttn	D3ZGE6_RAT (+1)	53 kDa	15.57	33.76	2.2	0.007	4	9	10.2%	22.0%
Uncharacterized protein	D4A269_RAT (+1)	14 kDa	7.27	17.36	2.4	0.033	3	4	46.0%	65.9%
Protein RGD1559864	D3ZB78_RAT (+1)	41 kDa	6.23	17.36	2.8	0.018	5	6	29.2%	37.2%
RCG61894, isoform CRA_a	G3V6L8_RAT	86 kDa	6.23	20.25	3.3	0.0052	3	8	9.4%	23.1%
Protein Tom1	Q5XI21_RAT	54 kDa	3.11	10.61	3.4	0.039	2	4	5.9%	16.9%
Cysteine-rich protein 2	CRIP2_RAT	23 kDa	5.19	19.29	3.7	0.0035	2	4	26.0%	52.9%
Isoform V3 of Versican core protein	sp|Q9ERB4-2|CSPG2_RAT (+2)	74 kDa	3.11	11.57	3.7	0.025	1	6	4.1%	15.6%
A-kinase anchor protein 5	AKAP5_RAT (+1)	76 kDa	2.08	8.68	4.2	0.043	2	2	5.0%	5.3%
Serine/threonine-protein phosphatase 2A 55 kDa regulatory subunit B alp	2ABA_RAT	52 kDa	5.19	23.15	4.5	0.00055	3	7	9.4%	23.5%
Serine/threonine-protein kinase PAK 1	PAK1_RAT	61 kDa	4.15	25.08	6.0	< 0.00010	2	7	6.1%	20.8%
Pyridoxal kinase	G3V647_RAT (+1)	35 kDa	1.04	6.75	6.5	0.044	1	4	3.2%	26.9%
Guanylate kinase	Q71RR7_RAT	22 kDa	1.04	7.72	7.4	0.025	1	3	6.1%	25.3%
ATPase inhibitor, mitochondrial	ATIF1_RAT	12 kDa	0.00	7.72	INF	0.0052	0	4	0.0%	15.0%
Toll-interacting protein	TOLIP_RAT	30 kDa	0.00	7.72	INF	0.0052	0	3	0.0%	15.0%
NAD-dependent protein deacylase sirtuin-5, mitochondrial	SIR5_RAT	34 kDa	0.00	7.72	INF	0.0052	0	4	0.0%	23.5%
MOB-like protein phocein	PHOCN_RAT	26 kDa	0.00	4.82	INF	0.037	0	3	0.0%	35.1%
Protein kinase C gamma type	KPCG_RAT	78 kDa	0.00	4.82	INF	0.037	0	2	0.0%	4.2%
Serum albumin	ALBU_RAT	69 kDa	0.00	6.75	INF	0.01	0	3	0.0%	6.4%
Rab GDP dissociation inhibitor beta	GDIB_RAT	51 kDa	0.00	26.04	INF	< 0.00010	0	2	0.0%	15.5%
