# Supplementary material for: Proteomics Analysis of Dorsal Striatum Reveals Changes in Synaptosomal Proteins following Methamphetamine Self-Administration in Rats
Source: PLoS One. 2015 Oct 20;10(10):e0139829. doi: 10.1371/journal.pone.0139829 (PMC4618287; doi:10.1371/journal.pone.0139829)
Supplement: S3 Table — (PDF) [file pone.0139829.s006.pdf]

**Supplementary Table S3.** Diseases and functions associated with the differentially expresssed proteins in methamphetamine self-administrated rats annotated by IPA. P-Value was set at≤ 1.00E-3.			
**Category**	**Diseases or functions annotation**	**p-Value**	**Differentially expressed proteins**
Neurological Disease	disorder of basal ganglia	8.64E-13	ACAT1,ACTB,ATP1A3,ATP6V1B2,CAMK2A,CNP,OXR1,PAK1,PCMT1,PEBP1,PPIA,PPP1R1B,SLC1A2,SOD1,SYN1,SYN2,TUBA1A,TUBA1B,TUBA4A,UCHL1,VCAN
Psychological Disorders	disorder of basal ganglia	8.64E-13	ACAT1,ACTB,ATP1A3,ATP6V1B2,CAMK2A,CNP,OXR1,PAK1,PCMT1,PEBP1,PPIA,PPP1R1B,SLC1A2,SOD1,SYN1,SYN2,TUBA1A,TUBA1B,TUBA4A,UCHL1,VCAN
Neurological Disease	Movement Disorders	8.30E-12	ACAT1,ACTB,ATP1A3,ATP6V1B2,CAMK2A,CNP,NCAM1,NRCAM,OXR1,PAK1,PCMT1,PEBP1,PPIA,PPP1R1B,SLC1A2,SOD1,SYN1,SYN2,TUBA1A,TUBA1B,TUBA4A,UCHL1,VCAN
Cellular Assembly and Organization	microtubule dynamics	1.57E-10	ACTB,AKAP5,BSN,CAMK2A,CFL1,CNP,CTTN,DNM1,DPYSL4,DPYSL5,NCAM1,NRCAM,PAK1,SOD1,STIP1,STRN,SYN1,Tpm1,TPPP,UCHL1,YWHAH
Cellular Function and Maintenance	microtubule dynamics	1.57E-10	ACTB,AKAP5,BSN,CAMK2A,CFL1,CNP,CTTN,DNM1,DPYSL4,DPYSL5,NCAM1,NRCAM,PAK1,SOD1,STIP1,STRN,SYN1,Tpm1,TPPP,UCHL1,YWHAH
Cellular Assembly and Organization	formation of cellular protrusions	2.82E-10	ACTB,AKAP5,BSN,CAMK2A,CFL1,CNP,CTTN,DNM1,DPYSL4,DPYSL5,NCAM1,NRCAM,PAK1,STIP1,STRN,Tpm1,UCHL1,YWHAH
Cellular Function and Maintenance	formation of cellular protrusions	2.82E-10	ACTB,AKAP5,BSN,CAMK2A,CFL1,CNP,CTTN,DNM1,DPYSL4,DPYSL5,NCAM1,NRCAM,PAK1,STIP1,STRN,Tpm1,UCHL1,YWHAH
Cell Morphology	formation of cellular protrusions	2.82E-10	ACTB,AKAP5,BSN,CAMK2A,CFL1,CNP,CTTN,DNM1,DPYSL4,DPYSL5,NCAM1,NRCAM,PAK1,STIP1,STRN,Tpm1,UCHL1,YWHAH
Cellular Assembly and Organization	organization of cytoskeleton	4.58E-10	ACTB,AKAP5,ALDOA,BSN,CAMK2A,CFL1,CNP,CTTN,DNM1,DPYSL4,DPYSL5,NCAM1,NRCAM,PAK1,SOD1,STIP1,STRN,SYN1,Tpm1,TPPP,UCHL1,YWHAH
Cellular Function and Maintenance	organization of cytoskeleton	4.58E-10	ACTB,AKAP5,ALDOA,BSN,CAMK2A,CFL1,CNP,CTTN,DNM1,DPYSL4,DPYSL5,NCAM1,NRCAM,PAK1,SOD1,STIP1,STRN,SYN1,Tpm1,TPPP,UCHL1,YWHAH
Neurological Disease	neuromuscular disease	6.11E-10	ACAT1,ACTB,ATP6V1B2,CAMK2A,CNP,OXR1,PAK1,PCMT1,PEBP1,PPIA,PPP1R1B,SLC1A2,SOD1,SYN1,SYN2,TUBA1A,TUBA1B,UCHL1,VCAN
Skeletal and Muscular Disorders	neuromuscular disease	6.11E-10	ACAT1,ACTB,ATP6V1B2,CAMK2A,CNP,OXR1,PAK1,PCMT1,PEBP1,PPIA,PPP1R1B,SLC1A2,SOD1,SYN1,SYN2,TUBA1A,TUBA1B,UCHL1,VCAN
Cell-To-Cell Signaling and Interaction	neurotransmission	2.87E-08	AKAP5,AMPH,BSN,CAMK2A,CNP,DNM1,NRCAM,Shank3,SLC1A2,SYN1,SYN2,UCHL1
Nervous System Development and Function	neurotransmission	2.87E-08	AKAP5,AMPH,BSN,CAMK2A,CNP,DNM1,NRCAM,Shank3,SLC1A2,SYN1,SYN2,UCHL1
Cell-To-Cell Signaling and Interaction	synaptic transmission	3.36E-08	AKAP5,AMPH,BSN,CAMK2A,CNP,NRCAM,Shank3,SLC1A2,SYN1,SYN2,UCHL1
Nervous System Development and Function	synaptic transmission	3.36E-08	AKAP5,AMPH,BSN,CAMK2A,CNP,NRCAM,Shank3,SLC1A2,SYN1,SYN2,UCHL1
Neurological Disease	progressive motor neuropathy	4.28E-08	ACTB,CNP,PAK1,PEBP1,SLC1A2,SOD1,SYN1,SYN2,TUBA1A,TUBA1B,UBQLN2,UCHL1,VCP
Cellular Assembly and Organization	formation of plasma membrane projections	6.58E-08	ACTB,AKAP5,BSN,CAMK2A,CNP,DPYSL4,NCAM1,NRCAM,PAK1,STIP1,STRN,UCHL1,YWHAH
Cellular Function and Maintenance	formation of plasma membrane projections	6.58E-08	ACTB,AKAP5,BSN,CAMK2A,CNP,DPYSL4,NCAM1,NRCAM,PAK1,STIP1,STRN,UCHL1,YWHAH
Cell Morphology	formation of plasma membrane projections	6.58E-08	ACTB,AKAP5,BSN,CAMK2A,CNP,DPYSL4,NCAM1,NRCAM,PAK1,STIP1,STRN,UCHL1,YWHAH
Cellular Development	formation of plasma membrane projections	6.58E-08	ACTB,AKAP5,BSN,CAMK2A,CNP,DPYSL4,NCAM1,NRCAM,PAK1,STIP1,STRN,UCHL1,YWHAH
Behavior	behavior	7.92E-08	ACAT1,AKAP5,AMPH,ATP1A3,CAMK2A,CRIP2,DNM1,NCAM1,NRCAM,PAK1,PPP1R1B,Shank3,SLC1A2,SOD1,SYN1,SYN2,UCHL1
Cellular Assembly and Organization	neuritogenesis	1.66E-07	ACTB,AKAP5,BSN,CAMK2A,CNP,DPYSL4,NCAM1,PAK1,STIP1,STRN,UCHL1,YWHAH
Cellular Function and Maintenance	neuritogenesis	1.66E-07	ACTB,AKAP5,BSN,CAMK2A,CNP,DPYSL4,NCAM1,PAK1,STIP1,STRN,UCHL1,YWHAH
Cell Morphology	neuritogenesis	1.66E-07	ACTB,AKAP5,BSN,CAMK2A,CNP,DPYSL4,NCAM1,PAK1,STIP1,STRN,UCHL1,YWHAH
Nervous System Development and Function	neuritogenesis	1.66E-07	ACTB,AKAP5,BSN,CAMK2A,CNP,DPYSL4,NCAM1,PAK1,STIP1,STRN,UCHL1,YWHAH
Cellular Development	neuritogenesis	1.66E-07	ACTB,AKAP5,BSN,CAMK2A,CNP,DPYSL4,NCAM1,PAK1,STIP1,STRN,UCHL1,YWHAH
Tissue Development	neuritogenesis	1.66E-07	ACTB,AKAP5,BSN,CAMK2A,CNP,DPYSL4,NCAM1,PAK1,STIP1,STRN,UCHL1,YWHAH
Behavior	locomotion	1.67E-07	ATP1A3,CAMK2A,CNP,NCAM1,NRCAM,PAK1,PPP1R1B,SOD1,STRN,UCHL1
Neurological Disease	Parkinson's disease	1.88E-07	CNP,PAK1,PEBP1,SOD1,SYN1,SYN2,TUBA1A,TUBA1B,UCHL1
Psychological Disorders	Parkinson's disease	1.88E-07	CNP,PAK1,PEBP1,SOD1,SYN1,SYN2,TUBA1A,TUBA1B,UCHL1
Skeletal and Muscular Disorders	Parkinson's disease	1.88E-07	CNP,PAK1,PEBP1,SOD1,SYN1,SYN2,TUBA1A,TUBA1B,UCHL1

Cell-To-Cell Signaling and Interaction	synaptic transmission of cells	2.25E-07	AMPH,BSN,CNP,NRCAM,Shank3,SLC1A2,SYN1,SYN2,UCHL1
Nervous System Development and Function	synaptic transmission of cells	2.25E-07	AMPH,BSN,CNP,NRCAM,Shank3,SLC1A2,SYN1,SYN2,UCHL1
Cell Death and Survival	cell death	2.81E-07	ACAT1,ACTB,ALDOA,CAMK2A,CFL1,CNP,CTTN,DNM1,DPYSL4,IMMT,NCAM1,NDUFS1,NRCAM,PAK1,PCMT1,Pdxk/RGD1566085,PEBP1,Phb,PPIA,PPP1R1B,PPP2R2A,SLC1A2,SOD1,STIP1,TUBA1A,UCHL1,UQCRFS1,VCAN,VCP,YWHAE,YWHAH,YWHAQ
Neurological Disease	Schizophrenia	4.51E-07	AMPH,ATP1A3,ATP6V1B2,CNP,DNM1,NCAM1,NDUFV1,PPP1R1B,SLC1A2,SYN2,YWHAE,YWHAH
Psychological Disorders	Schizophrenia	4.51E-07	AMPH,ATP1A3,ATP6V1B2,CNP,DNM1,NCAM1,NDUFV1,PPP1R1B,SLC1A2,SYN2,YWHAE,YWHAH
Hereditary Disorder	Schizophrenia	4.51E-07	AMPH,ATP1A3,ATP6V1B2,CNP,DNM1,NCAM1,NDUFV1,PPP1R1B,SLC1A2,SYN2,YWHAE,YWHAH
Tissue Development	growth of nervous tissue	1.77E-06	CAMK2A,CFL1,CNP,DPYSL5,NCAM1,NRCAM,PAK1,SOD1,SYN1,Tpm3,TUBA1A,VCAN
Behavior	spatial learning	2.02E-06	AKAP5,AMPH,ATP1A3,CAMK2A,NCAM1,NRCAM,PPP1R1B
Cell-To-Cell Signaling and Interaction	long-term potentiation	2.06E-06	AKAP5,CAMK2A,DPYSL4,NCAM1,PAK1,PPP1R1B,Shank3,SOD1
Nervous System Development and Function	long-term potentiation	2.06E-06	AKAP5,CAMK2A,DPYSL4,NCAM1,PAK1,PPP1R1B,Shank3,SOD1
Cell-To-Cell Signaling and Interaction	synaptic depression	2.45E-06	AKAP5,CAMK2A,PEBP1,PPP1R1B,SYN1,SYN2
Cell Death and Survival	necrosis	2.54E-06	ACAT1,ACTB,ALDOA,CNP,CTTN,DNM1,DPYSL4,IMMT,NCAM1,NRCAM,PAK1,PEBP1,PPIA,PPP1R1B,PPP2R2A,SLC1A2,SOD1,STIP1,TUBA1A,UCHL1,UQCRFS1,VCAN,VCP,YWHAE,YWHAH,YWHAQ
Neurological Disease	Huntington's Disease	2.60E-06	ACAT1,ACTB,ATP6V1B2,CAMK2A,OXR1,PCMT1,PPIA,PPP1R1B,SLC1A2,TUBA1A,UCHL1,VCAN
Psychological Disorders	Huntington's Disease	2.60E-06	ACAT1,ACTB,ATP6V1B2,CAMK2A,OXR1,PCMT1,PPIA,PPP1R1B,SLC1A2,TUBA1A,UCHL1,VCAN
Skeletal and Muscular Disorders	Huntington's Disease	2.60E-06	ACAT1,ACTB,ATP6V1B2,CAMK2A,OXR1,PCMT1,PPIA,PPP1R1B,SLC1A2,TUBA1A,UCHL1,VCAN
Hereditary Disorder	Huntington's Disease	2.60E-06	ACAT1,ACTB,ATP6V1B2,CAMK2A,OXR1,PCMT1,PPIA,PPP1R1B,SLC1A2,TUBA1A,UCHL1,VCAN
Cell Morphology	morphology of neurons	3.79E-06	CAMK2A,DNM1,DPYSL4,NCAM1,NRCAM,PCMT1,SYN1,SYN2,TUBA1A,VCAN
Nervous System Development and Function	morphology of neurons	3.79E-06	CAMK2A,DNM1,DPYSL4,NCAM1,NRCAM,PCMT1,SYN1,SYN2,TUBA1A,VCAN
Tissue Morphology	morphology of neurons	3.79E-06	CAMK2A,DNM1,DPYSL4,NCAM1,NRCAM,PCMT1,SYN1,SYN2,TUBA1A,VCAN
Behavior	conditioning	4.32E-06	AMPH,CAMK2A,NCAM1,PAK1,PPP1R1B,SYN1,UCHL1
Cellular Assembly and Organization	quantity of vesicles	9.10E-06	BSN,CAMK2A,DNM1,SOD1,SYN1
Nervous System Development and Function	morphology of nervous tissue	9.45E-06	CAMK2A,DNM1,DPYSL4,NCAM1,NRCAM,PCMT1,SOD1,SYN1,SYN2,TUBA1A,VCAN
Tissue Morphology	morphology of nervous tissue	9.45E-06	CAMK2A,DNM1,DPYSL4,NCAM1,NRCAM,PCMT1,SOD1,SYN1,SYN2,TUBA1A,VCAN
Cellular Assembly and Organization	association of synaptic vesicles	9.64E-06	SYN1,SYN2
Cell-To-Cell Signaling and Interaction	association of synaptic vesicles	9.64E-06	SYN1,SYN2
Nervous System Development and Function	association of synaptic vesicles	9.64E-06	SYN1,SYN2
Behavior	social exploration	9.78E-06	NRCAM,Shank3,SYN1,SYN2
Cellular Assembly and Organization	quantity of cellular protrusions	1.03E-05	AKAP5,CNP,CTTN,DPYSL4,PAK1,SOD1
Cellular Function and Maintenance	quantity of cellular protrusions	1.03E-05	AKAP5,CNP,CTTN,DPYSL4,PAK1,SOD1
Nervous System Development and Function	morphology of nervous system	1.37E-05	CAMK2A,DNM1,DPYSL4,NCAM1,NRCAM,PCMT1,SLC1A2,SOD1,SYN1,SYN2,TUBA1A,VCAN,YWHAE
Cell Death and Survival	cell death of cervical cancer cell lines	1.42E-05	IMMT,PAK1,PPIA,PPP2R2A,SOD1,UCHL1,VCP,YWHAH
Behavior	cognition	1.58E-05	ACAT1,AKAP5,AMPH,ATP1A3,CAMK2A,NCAM1,NRCAM,PPP1R1B,UCHL1
Neurological Disease	sporadic amyotrophic lateral sclerosis	1.59E-05	ACTB,SLC1A2,SOD1
Neurological Disease	paralysis	1.83E-05	ATP1A3,NRCAM,SLC1A2,SOD1,TUBA1A,TUBA4A
Cell Morphology	morphology of cells	2.09E-05	AKAP5,AMPH,ATP6V1B2,CAMK2A,CFL1,CTTN,DNM1,DPYSL4,NCAM1,NDUFS1,NRCAM,PAK1,PCMT1,PEBP1,SOD1,SYN1,SYN2,Tpm1,Tpm3,TUBA1A,VCAN
Cellular Assembly and Organization	outgrowth of neurites	2.22E-05	DPYSL5,NCAM1,NRCAM,PAK1,SOD1,SYN1,Tpm3,TUBA1A,VCAN
Cell Morphology	outgrowth of neurites	2.22E-05	DPYSL5,NCAM1,NRCAM,PAK1,SOD1,SYN1,Tpm3,TUBA1A,VCAN
Nervous System Development and Function	outgrowth of neurites	2.22E-05	DPYSL5,NCAM1,NRCAM,PAK1,SOD1,SYN1,Tpm3,TUBA1A,VCAN
Tissue Development	outgrowth of neurites	2.22E-05	DPYSL5,NCAM1,NRCAM,PAK1,SOD1,SYN1,Tpm3,TUBA1A,VCAN
Cellular Assembly and Organization	synaptogenesis	2.28E-05	BSN,NCAM1,NRCAM,PAK1,SYN1,SYN2

Cellular Function and Maintenance	synaptogenesis	2.28E-05	BSN,NCAM1,NRCAM,PAK1,SYN1,SYN2
Cell-To-Cell Signaling and Interaction	synaptogenesis	2.28E-05	BSN,NCAM1,NRCAM,PAK1,SYN1,SYN2
Nervous System Development and Function	synaptogenesis	2.28E-05	BSN,NCAM1,NRCAM,PAK1,SYN1,SYN2
Tissue Development	synaptogenesis	2.28E-05	BSN,NCAM1,NRCAM,PAK1,SYN1,SYN2
Cancer	choriocarcinoma	2.30E-05	PAK1,TUBA1A,TUBA4A
Organismal Injury and Abnormalities	choriocarcinoma	2.30E-05	PAK1,TUBA1A,TUBA4A
Reproductive System Disease	choriocarcinoma	2.30E-05	PAK1,TUBA1A,TUBA4A
Cellular Function and Maintenance	transmembrane potential	3.01E-05	DLD,IMMT,NDUFS1,Phb,PPP1R1B,SOD1,YWHAE
Cell Morphology	transmembrane potential	3.01E-05	DLD,IMMT,NDUFS1,Phb,PPP1R1B,SOD1,YWHAE
Neurological Disease	tauopathy	3.49E-05	ACTB,AKAP5,CAMK2A,CNP,PAK1,SLC1A2,SOD1,TUBA1A,TUBA4A,UCHL1
Psychological Disorders	tauopathy	3.49E-05	ACTB,AKAP5,CAMK2A,CNP,PAK1,SLC1A2,SOD1,TUBA1A,TUBA4A,UCHL1
Cell Death and Survival	cell death of neuroblastoma cell lines	3.65E-05	NCAM1,PAK1,PEBP1,SOD1,TUBA1A,YWHAE
Cellular Assembly and Organization	recycling of synaptic vesicles	4.31E-05	AMPH,DNM1,SYN1
Nervous System Development and Function	recycling of synaptic vesicles	4.31E-05	AMPH,DNM1,SYN1
Nucleic Acid Metabolism	metabolism of nucleoside triphosphate	4.55E-05	ALDOA,ATP5B,GUK1,NDUFS1,SOD1,VCP
Small Molecule Biochemistry	metabolism of nucleoside triphosphate	4.55E-05	ALDOA,ATP5B,GUK1,NDUFS1,SOD1,VCP
Cell Death and Survival	cell death of tumor cell lines	4.79E-05	CTTN,DNM1,IMMT,NCAM1,PAK1,PEBP1,PPIA,PPP1R1B,PPP2R2A,SOD1,TUBA1A,UCHL1,UQCRFS1,VCAN,VCP,YWHAE,YWHAH
Cell Death and Survival	apoptosis	5.39E-05	ALDOA,CAMK2A,CFL1,CNP,CTTN,DNM1,IMMT,NCAM1,NDUFS1,PAK1,PCMT1,Pdxk/RGD1566085,PEBP1,Phb,PPIA,PPP1R1B,PPP2R2A,SOD1,STIP1,UCHL1,VCAN,VCP,YWHAE,YWHAQ
Neurological Disease	seizure disorder	5.71E-05	AKAP5,AMPH,BSN,NCAM1,PCMT1,PPP1R1B,SLC1A2,SYN1,SYN2
Cell Morphology	abnormal morphology of neurons	6.42E-05	CAMK2A,DNM1,NCAM1,NRCAM,PCMT1,SYN1,SYN2,VCAN
Nervous System Development and Function	abnormal morphology of neurons	6.42E-05	CAMK2A,DNM1,NCAM1,NRCAM,PCMT1,SYN1,SYN2,VCAN
Tissue Morphology	abnormal morphology of neurons	6.42E-05	CAMK2A,DNM1,NCAM1,NRCAM,PCMT1,SYN1,SYN2,VCAN
Cellular Function and Maintenance	transmembrane potential of mitochondria	6.88E-05	IMMT,NDUFS1,Phb,PPP1R1B,SOD1,YWHAE
Cell Morphology	transmembrane potential of mitochondria	6.88E-05	IMMT,NDUFS1,Phb,PPP1R1B,SOD1,YWHAE
Cellular Movement	homing of embryonic cell lines	7.21E-05	DNM1,PAK1,PPIA
Embryonic Development	homing of embryonic cell lines	7.21E-05	DNM1,PAK1,PPIA
Cellular Movement	homing of epithelial cell lines	7.21E-05	DNM1,PAK1,PPIA
Hair and Skin Development and Function	homing of epithelial cell lines	7.21E-05	DNM1,PAK1,PPIA
Nervous System Development and Function	morphology of cerebral cortex	7.23E-05	NCAM1,PCMT1,SLC1A2,SYN1,TUBA1A,YWHAE
Organ Morphology	morphology of cerebral cortex	7.23E-05	NCAM1,PCMT1,SLC1A2,SYN1,TUBA1A,YWHAE
Organismal Development	morphology of cerebral cortex	7.23E-05	NCAM1,PCMT1,SLC1A2,SYN1,TUBA1A,YWHAE
Behavior	learning	7.39E-05	ACAT1,AKAP5,AMPH,ATP1A3,CAMK2A,NCAM1,NRCAM,PPP1R1B
Cancer	small cell lung cancer	8.94E-05	NCAM1,TUBA1A,TUBA4A,YWHAE
Respiratory Disease	small cell lung cancer	8.94E-05	NCAM1,TUBA1A,TUBA4A,YWHAE
Neurological Disease	delay in amyotrophic lateral sclerosis	9.58E-05	SLC1A2,SOD1
Respiratory Disease	allergic pulmonary eosinophilia	1.12E-04	ACO2,ACTB,ALDOA
Hematological Disease	allergic pulmonary eosinophilia	1.12E-04	ACO2,ACTB,ALDOA
Immunological Disease	allergic pulmonary eosinophilia	1.12E-04	ACO2,ACTB,ALDOA
Inflammatory Disease	allergic pulmonary eosinophilia	1.12E-04	ACO2,ACTB,ALDOA
Inflammatory Response	allergic pulmonary eosinophilia	1.12E-04	ACO2,ACTB,ALDOA
Nucleic Acid Metabolism	metabolism of purine nucleotide	1.13E-04	ATP5B,GUK1,MDH2,NDUFS1,VCP
Small Molecule Biochemistry	metabolism of purine nucleotide	1.13E-04	ATP5B,GUK1,MDH2,NDUFS1,VCP
Neurological Disease	seizures	1.23E-04	AKAP5,AMPH,BSN,NCAM1,PCMT1,SLC1A2,SYN1,SYN2

Neurological Disease	bipolar disorder	1.24E-04	ATP1A3,CAMK2A,NCAM1,NDUFS1,SYN1,SYN2,YWHAH
Psychological Disorders	bipolar disorder	1.24E-04	ATP1A3,CAMK2A,NCAM1,NDUFS1,SYN1,SYN2,YWHAH
Neurological Disease	amyotrophic lateral sclerosis	1.26E-04	ACTB,SLC1A2,SOD1,TUBA1A,UBQLN2,VCP
Cellular Movement	homing of kidney cell lines	1.36E-04	DNM1,PAK1,PPIA
Renal and Urological System Development and Function	homing of kidney cell lines	1.36E-04	DNM1,PAK1,PPIA
Cell Death and Survival	apoptosis of tumor cell lines	1.37E-04	CTTN,DNM1,IMMT,NCAM1,PAK1,PEBP1,PPIA,PPP1R1B,PPP2R2A,SOD1,UCHL1,VCAN,VCP,YWHAE
Nervous System Development and Function	abnormal morphology of nervous system	1.39E-04	CAMK2A,DNM1,NCAM1,NRCAM,PCMT1,SLC1A2,SOD1,SYN1,SYN2,VCAN,YWHAE
Cellular Assembly and Organization	neuritogenesis of hippocampal neurons	1.43E-04	NCAM1,STIP1
Cellular Function and Maintenance	neuritogenesis of hippocampal neurons	1.43E-04	NCAM1,STIP1
Cell Morphology	neuritogenesis of hippocampal neurons	1.43E-04	NCAM1,STIP1
Nervous System Development and Function	neuritogenesis of hippocampal neurons	1.43E-04	NCAM1,STIP1
Cellular Development	neuritogenesis of hippocampal neurons	1.43E-04	NCAM1,STIP1
Tissue Development	neuritogenesis of hippocampal neurons	1.43E-04	NCAM1,STIP1
Embryonic Development	neuritogenesis of hippocampal neurons	1.43E-04	NCAM1,STIP1
Organismal Development	neuritogenesis of hippocampal neurons	1.43E-04	NCAM1,STIP1
Organ Development	neuritogenesis of hippocampal neurons	1.43E-04	NCAM1,STIP1
Behavior	contextual conditioning	1.66E-04	AMPH,CAMK2A,NCAM1,UCHL1
Cellular Assembly and Organization	quantity of plasma membrane projections	1.66E-04	CNP,DPYSL4,PAK1,SOD1
Cellular Function and Maintenance	quantity of plasma membrane projections	1.66E-04	CNP,DPYSL4,PAK1,SOD1
Neurological Disease	Dementia	1.79E-04	ACTB,AKAP5,CAMK2A,CNP,PAK1,SLC1A2,SOD1,UCHL1,VCP
Psychological Disorders	Dementia	1.79E-04	ACTB,AKAP5,CAMK2A,CNP,PAK1,SLC1A2,SOD1,UCHL1,VCP
Cell Morphology	abnormal morphology of axons	1.81E-04	NCAM1,NRCAM,PCMT1,VCAN
Nervous System Development and Function	abnormal morphology of axons	1.81E-04	NCAM1,NRCAM,PCMT1,VCAN
Tissue Morphology	abnormal morphology of axons	1.81E-04	NCAM1,NRCAM,PCMT1,VCAN
Cell Morphology	morphology of neurites	1.91E-04	DPYSL4,NCAM1,NRCAM,PCMT1,VCAN
Nervous System Development and Function	morphology of neurites	1.91E-04	DPYSL4,NCAM1,NRCAM,PCMT1,VCAN
Tissue Morphology	morphology of neurites	1.91E-04	DPYSL4,NCAM1,NRCAM,PCMT1,VCAN
Cellular Assembly and Organization	size of dendritic spines	2.00E-04	AKAP5,CAMK2A
Cellular Function and Maintenance	size of dendritic spines	2.00E-04	AKAP5,CAMK2A
Cell Morphology	size of dendritic spines	2.00E-04	AKAP5,CAMK2A
Nervous System Development and Function	size of dendritic spines	2.00E-04	AKAP5,CAMK2A
Cellular Development	size of dendritic spines	2.00E-04	AKAP5,CAMK2A
Tissue Development	size of dendritic spines	2.00E-04	AKAP5,CAMK2A
Embryonic Development	size of dendritic spines	2.00E-04	AKAP5,CAMK2A
Neurological Disease	type 1 lissencephaly	2.00E-04	TUBA1A,YWHAE
Hereditary Disorder	type 1 lissencephaly	2.00E-04	TUBA1A,YWHAE
Organismal Injury and Abnormalities	type 1 lissencephaly	2.00E-04	TUBA1A,YWHAE
Developmental Disorder	type 1 lissencephaly	2.00E-04	TUBA1A,YWHAE
Molecular Transport	transport of inorganic cation	2.06E-04	ATP1A3,ATP5B,ATP6V1B2,CAMK2A,SLC1A2,YWHAE,YWHAH
Cellular Assembly and Organization	morphogenesis of neurites	2.16E-04	AKAP5,BSN,CAMK2A,DPYSL4,NCAM1,PAK1,YWHAH
Cellular Function and Maintenance	morphogenesis of neurites	2.16E-04	AKAP5,BSN,CAMK2A,DPYSL4,NCAM1,PAK1,YWHAH

Cell Morphology	morphogenesis of neurites	2.16E-04	AKAP5,BSN,CAMK2A,DPYSL4,NCAM1,PAK1,YWHAH
Nervous System Development and Function	morphogenesis of neurites	2.16E-04	AKAP5,BSN,CAMK2A,DPYSL4,NCAM1,PAK1,YWHAH
Cellular Development	morphogenesis of neurites	2.16E-04	AKAP5,BSN,CAMK2A,DPYSL4,NCAM1,PAK1,YWHAH
Tissue Development	morphogenesis of neurites	2.16E-04	AKAP5,BSN,CAMK2A,DPYSL4,NCAM1,PAK1,YWHAH
Cellular Assembly and Organization	branching of neurites	2.16E-04	AKAP5,BSN,CAMK2A,DPYSL4,NCAM1,PAK1
Cellular Function and Maintenance	branching of neurites	2.16E-04	AKAP5,BSN,CAMK2A,DPYSL4,NCAM1,PAK1
Cell Morphology	branching of neurites	2.16E-04	AKAP5,BSN,CAMK2A,DPYSL4,NCAM1,PAK1
Nervous System Development and Function	branching of neurites	2.16E-04	AKAP5,BSN,CAMK2A,DPYSL4,NCAM1,PAK1
Cellular Development	branching of neurites	2.16E-04	AKAP5,BSN,CAMK2A,DPYSL4,NCAM1,PAK1
Tissue Development	branching of neurites	2.16E-04	AKAP5,BSN,CAMK2A,DPYSL4,NCAM1,PAK1
Embryonic Development	branching of neurites	2.16E-04	AKAP5,BSN,CAMK2A,DPYSL4,NCAM1,PAK1
Cell-To-Cell Signaling and Interaction	long term depression	2.52E-04	AKAP5,CAMK2A,PEBP1,PPP1R1B
Nervous System Development and Function	long term depression	2.52E-04	AKAP5,CAMK2A,PEBP1,PPP1R1B
Cellular Assembly and Organization	formation of cytoskeleton	2.55E-04	ACTB,CFL1,CNP,CTTN,PAK1,Tpm3,TPPP
Cellular Movement	cell movement	2.62E-04	ACTB,ALDOA,CFL1,CNP,CRIP2,CTTN,DNM1,NCAM1,NRCAM,PAK1,PEBP1,PPIA,SLC1A2,SOD1,Tpm1,TUBA1A,VCAN,VCP,YWHAE
Cell-To-Cell Signaling and Interaction	long term depression of cells	2.66E-04	CAMK2A,PEBP1,PPP1R1B
Nervous System Development and Function	long term depression of cells	2.66E-04	CAMK2A,PEBP1,PPP1R1B
Cell Morphology	morphology of cerebral cortex cells	2.66E-04	PCMT1,SYN1,TUBA1A
Nervous System Development and Function	morphology of cerebral cortex cells	2.66E-04	PCMT1,SYN1,TUBA1A
Organ Morphology	morphology of cerebral cortex cells	2.66E-04	PCMT1,SYN1,TUBA1A
Organismal Development	morphology of cerebral cortex cells	2.66E-04	PCMT1,SYN1,TUBA1A
Molecular Transport	accumulation of hydrogen peroxide	2.67E-04	NDUFS1,SOD1
Free Radical Scavenging	accumulation of hydrogen peroxide	2.67E-04	NDUFS1,SOD1
Cellular Assembly and Organization	growth of filopodia	2.67E-04	DPYSL5,PAK1
Nucleic Acid Metabolism	metabolism of nucleic acid component or derivative	2.85E-04	AKAP5,ALDOA,ATP5B,GUK1,MDH2,NDUFS1,PCMT1,SOD1,VCP
Cellular Assembly and Organization	quantity of synaptic vesicles	2.86E-04	CAMK2A,DNM1,SYN1
Nervous System Development and Function	quantity of synaptic vesicles	2.86E-04	CAMK2A,DNM1,SYN1
Cellular Movement	cell movement of embryonic cell lines	2.94E-04	CTTN,DNM1,PAK1,PPIA
Embryonic Development	cell movement of embryonic cell lines	2.94E-04	CTTN,DNM1,PAK1,PPIA
Free Radical Scavenging	production of reactive oxygen species	3.19E-04	ACTB,CTTN,IMMT,NDUFS1,PPIA,SOD1,UQCRFS1
Post-Translational Modification	carbonylation of protein	3.42E-04	Phb,SOD1
Nucleic Acid Metabolism	metabolism of ATP	3.78E-04	ATP5B,GUK1,NDUFS1,VCP
Small Molecule Biochemistry	metabolism of ATP	3.78E-04	ATP5B,GUK1,NDUFS1,VCP
Energy Production	metabolism of ATP	3.78E-04	ATP5B,GUK1,NDUFS1,VCP
Neurological Disease	progressive supranuclear palsy	4.03E-04	SLC1A2,TUBA1A,TUBA4A
Psychological Disorders	progressive supranuclear palsy	4.03E-04	SLC1A2,TUBA1A,TUBA4A
Ophthalmic Disease	progressive supranuclear palsy	4.03E-04	SLC1A2,TUBA1A,TUBA4A
Cellular Assembly and Organization	formation of neurites	4.13E-04	AKAP5,NRCAM,PAK1,STRN,YWHAH
Cellular Function and Maintenance	formation of neurites	4.13E-04	AKAP5,NRCAM,PAK1,STRN,YWHAH

Cell Morphology	formation of neurites	4.13E-04	AKAP5,NRCAM,PAK1,STRN,YWHAH
Nervous System Development and Function	formation of neurites	4.13E-04	AKAP5,NRCAM,PAK1,STRN,YWHAH
Cellular Development	formation of neurites	4.13E-04	AKAP5,NRCAM,PAK1,STRN,YWHAH
Molecular Transport	transport of ion	4.15E-04	AKAP5,ATP1A3,ATP5B,ATP6V1B2,CAMK2A,SLC1A2,YWHAE,YWHAH
Cellular Movement	cell movement of epithelial cell lines	4.19E-04	CTTN,DNM1,PAK1,PPIA
Hair and Skin Development and Function	cell movement of epithelial cell lines	4.19E-04	CTTN,DNM1,PAK1,PPIA
Neurological Disease	autosomal dominant motor neuron disease	4.27E-04	SOD1,UCHL1
Cellular Assembly and Organization	dendritic growth/branching	4.32E-04	AKAP5,BSN,CAMK2A,DPYSL4,PAK1
Cellular Function and Maintenance	dendritic growth/branching	4.32E-04	AKAP5,BSN,CAMK2A,DPYSL4,PAK1
Cell Morphology	dendritic growth/branching	4.32E-04	AKAP5,BSN,CAMK2A,DPYSL4,PAK1
Nervous System Development and Function	dendritic growth/branching	4.32E-04	AKAP5,BSN,CAMK2A,DPYSL4,PAK1
Cellular Development	dendritic growth/branching	4.32E-04	AKAP5,BSN,CAMK2A,DPYSL4,PAK1
Tissue Development	dendritic growth/branching	4.32E-04	AKAP5,BSN,CAMK2A,DPYSL4,PAK1
Embryonic Development	dendritic growth/branching	4.32E-04	AKAP5,BSN,CAMK2A,DPYSL4,PAK1
Cancer	benign neoplasia	4.38E-04	ACAT1,ALDOA,CNP,DPYSL4,NRCAM,PAK1,TUBA1A,TUBA4A,VCAN,VCP
Nucleic Acid Metabolism	metabolism of nucleotide	4.50E-04	AKAP5,ALDOA,ATP5B,GUK1,MDH2,NDUFS1,SOD1,VCP
Small Molecule Biochemistry	metabolism of nucleotide	4.50E-04	AKAP5,ALDOA,ATP5B,GUK1,MDH2,NDUFS1,SOD1,VCP
Cancer	Waldenstrom's macroglobulinemia	4.73E-04	AKAP5,AMPH,STIP1,STRN,YWHAE
Hematological Disease	Waldenstrom's macroglobulinemia	4.73E-04	AKAP5,AMPH,STIP1,STRN,YWHAE
Immunological Disease	Waldenstrom's macroglobulinemia	4.73E-04	AKAP5,AMPH,STIP1,STRN,YWHAE
Nervous System Development and Function	morphology of brain	4.81E-04	NCAM1,NRCAM,PCMT1,SLC1A2,SYN1,SYN2,TUBA1A,YWHAE
Organ Morphology	morphology of brain	4.81E-04	NCAM1,NRCAM,PCMT1,SLC1A2,SYN1,SYN2,TUBA1A,YWHAE
Organismal Development	morphology of brain	4.81E-04	NCAM1,NRCAM,PCMT1,SLC1A2,SYN1,SYN2,TUBA1A,YWHAE
Nervous System Development and Function	abnormal morphology of cerebral cortex	4.83E-04	NCAM1,PCMT1,SLC1A2,SYN1,YWHAE
Organ Morphology	abnormal morphology of cerebral cortex	4.83E-04	NCAM1,PCMT1,SLC1A2,SYN1,YWHAE
Organismal Development	abnormal morphology of cerebral cortex	4.83E-04	NCAM1,PCMT1,SLC1A2,SYN1,YWHAE
Small Molecule Biochemistry	metabolism of oxalacetic acid	5.21E-04	GOT2,MDH2
Cellular Assembly and Organization	size of plasma membrane projections	5.21E-04	CNP,SOD1
Small Molecule Biochemistry	synthesis of acidic amino acid	5.21E-04	GOT2,VCAN
Amino Acid Metabolism	synthesis of acidic amino acid	5.21E-04	GOT2,VCAN
Cell-To-Cell Signaling and Interaction	excitatory postsynaptic potential	5.44E-04	CAMK2A,DNM1,Shank3,SYN1
Nervous System Development and Function	excitatory postsynaptic potential	5.44E-04	CAMK2A,DNM1,Shank3,SYN1
Cellular Assembly and Organization	formation of dendrites	5.44E-04	AKAP5,PAK1,STRN,YWHAH
Cellular Function and Maintenance	formation of dendrites	5.44E-04	AKAP5,PAK1,STRN,YWHAH
Cell Morphology	formation of dendrites	5.44E-04	AKAP5,PAK1,STRN,YWHAH
Nervous System Development and Function	formation of dendrites	5.44E-04	AKAP5,PAK1,STRN,YWHAH
Cellular Development	formation of dendrites	5.44E-04	AKAP5,PAK1,STRN,YWHAH
Tissue Development	formation of dendrites	5.44E-04	AKAP5,PAK1,STRN,YWHAH
Cell-To-Cell Signaling and Interaction	efflux of dopamine	6.24E-04	CAMK2A,CNP
Small Molecule Biochemistry	efflux of dopamine	6.24E-04	CAMK2A,CNP

Molecular Transport	efflux of dopamine	6.24E-04	CAMK2A,CNP
Drug Metabolism	efflux of dopamine	6.24E-04	CAMK2A,CNP
Tissue Development	formation of neural fold	6.24E-04	CFL1,NCAM1
Embryonic Development	formation of neural fold	6.24E-04	CFL1,NCAM1
Organismal Development	formation of neural fold	6.24E-04	CFL1,NCAM1
Cellular Assembly and Organization	size of nerve ending	6.24E-04	NCAM1,SYN1
Cell Morphology	size of nerve ending	6.24E-04	NCAM1,SYN1
Nervous System Development and Function	size of nerve ending	6.24E-04	NCAM1,SYN1
Cellular Assembly and Organization	stabilization of actin filaments	6.24E-04	CFL1,CTTN
Cellular Function and Maintenance	receptor-mediated endocytosis	6.35E-04	ATP5B,ATP6V1B2,CTTN,DNM1
Cell Death and Survival	cell death of pheochromocytoma cell lines	6.54E-04	PPIA,SOD1,VCAN,VCP
Cell Morphology	morphology of brain cells	6.74E-04	NRCAM,PCMT1,SYN1,TUBA1A
Nervous System Development and Function	morphology of brain cells	6.74E-04	NRCAM,PCMT1,SYN1,TUBA1A
Organ Morphology	morphology of brain cells	6.74E-04	NRCAM,PCMT1,SYN1,TUBA1A
Organismal Development	morphology of brain cells	6.74E-04	NRCAM,PCMT1,SYN1,TUBA1A
Molecular Transport	transport of H+	6.82E-04	ATP1A3,ATP5B,ATP6V1B2
Neurological Disease	Alzheimer's disease	7.02E-04	ACTB,AKAP5,CAMK2A,CNP,PAK1,SLC1A2,SOD1,UCHL1
Psychological Disorders	Alzheimer's disease	7.02E-04	ACTB,AKAP5,CAMK2A,CNP,PAK1,SLC1A2,SOD1,UCHL1
Metabolic Disease	Alzheimer's disease	7.02E-04	ACTB,AKAP5,CAMK2A,CNP,PAK1,SLC1A2,SOD1,UCHL1
Nervous System Development and Function	abnormal morphology of stratum pyramidale	7.36E-04	NCAM1,YWHAE
Organ Morphology	abnormal morphology of stratum pyramidale	7.36E-04	NCAM1,YWHAE
Organismal Development	abnormal morphology of stratum pyramidale	7.36E-04	NCAM1,YWHAE
Cellular Assembly and Organization	abnormal morphology of synaptic vesicles	7.36E-04	CAMK2A,DNM1
Cell Morphology	abnormal morphology of synaptic vesicles	7.36E-04	CAMK2A,DNM1
Nervous System Development and Function	abnormal morphology of synaptic vesicles	7.36E-04	CAMK2A,DNM1
Tissue Morphology	abnormal morphology of synaptic vesicles	7.36E-04	CAMK2A,DNM1
Organ Morphology	relaxation of vascular smooth muscle	7.36E-04	CNP,SOD1
Skeletal and Muscular System Development and Function	relaxation of vascular smooth muscle	7.36E-04	CNP,SOD1
Cellular Function and Maintenance	endocytosis	8.00E-04	AMPH,ATP5B,ATP6V1B2,CTTN,DNM1,PAK1
Molecular Transport	transport of monovalent inorganic cation	8.23E-04	ATP1A3,ATP5B,ATP6V1B2,SLC1A2,YWHAH
Cell-To-Cell Signaling and Interaction	excitatory postsynaptic potential of neurons	8.38E-04	CAMK2A,Shank3,SYN1
Nervous System Development and Function	excitatory postsynaptic potential of neurons	8.38E-04	CAMK2A,Shank3,SYN1
Organismal Injury and Abnormalities	Letterer-Siwe disease	8.56E-04	TUBA1A,TUBA4A
Immunological Disease	Letterer-Siwe disease	8.56E-04	TUBA1A,TUBA4A
Cancer	advanced testicular carcinoma	8.56E-04	TUBA1A,TUBA4A
Organismal Injury and Abnormalities	advanced testicular carcinoma	8.56E-04	TUBA1A,TUBA4A
Reproductive System Disease	advanced testicular carcinoma	8.56E-04	TUBA1A,TUBA4A
Endocrine System Disorders	advanced testicular carcinoma	8.56E-04	TUBA1A,TUBA4A
Neurological Disease	familial amyotrophic lateral sclerosis	8.56E-04	SLC1A2,SOD1
Hereditary Disorder	familial amyotrophic lateral sclerosis	8.56E-04	SLC1A2,SOD1

Cancer	histiocytic lymphoma	8.56E-04	TUBA1A,TUBA4A
Hematological Disease	histiocytic lymphoma	8.56E-04	TUBA1A,TUBA4A
Immunological Disease	histiocytic lymphoma	8.56E-04	TUBA1A,TUBA4A
Cell-To-Cell Signaling and Interaction	synaptic transmission of hippocampal neurons	8.56E-04	Shank3,UCHL1
Nervous System Development and Function	synaptic transmission of hippocampal neurons	8.56E-04	Shank3,UCHL1
Cancer	teratocarcinoma	8.56E-04	TUBA1A,TUBA4A
Organismal Injury and Abnormalities	teratocarcinoma	8.56E-04	TUBA1A,TUBA4A
Reproductive System Disease	teratocarcinoma	8.56E-04	TUBA1A,TUBA4A
Cell Death and Survival	cell death of carcinoma cell lines	8.71E-04	CTTN,PPP1R1B,SOD1,UCHL1,UQCRFS1
Cellular Movement	invasion of fibroblast cell lines	9.23E-04	ACAT1,CTTN,NRCAM
Cellular Assembly and Organization	quantity of actin stress fibers	9.23E-04	CFL1,PAK1,Tpm1
Cell-To-Cell Signaling and Interaction	quantity of actin stress fibers	9.23E-04	CFL1,PAK1,Tpm1
Tissue Development	quantity of actin stress fibers	9.23E-04	CFL1,PAK1,Tpm1
Cancer	plasma cell dyscrasia	9.79E-04	AKAP5,AMPH,NCAM1,STIP1,STRN,YWHAE
Hematological Disease	plasma cell dyscrasia	9.79E-04	AKAP5,AMPH,NCAM1,STIP1,STRN,YWHAE
Immunological Disease	plasma cell dyscrasia	9.79E-04	AKAP5,AMPH,NCAM1,STIP1,STRN,YWHAE
Cell Death and Survival	cell death of pyramidal neurons	9.86E-04	SLC1A2,SOD1
Neurological Disease	metastatic pheochromocytoma	9.86E-04	TUBA1A,TUBA4A
Cancer	metastatic pheochromocytoma	9.86E-04	TUBA1A,TUBA4A
Cancer	refractory Philadelphia chromosome-negative acute lymphoblastic leukemia	9.86E-04	TUBA1A,TUBA4A
Hematological Disease	refractory Philadelphia chromosome-negative acute lymphoblastic leukemia	9.86E-04	TUBA1A,TUBA4A
Immunological Disease	refractory Philadelphia chromosome-negative acute lymphoblastic leukemia	9.86E-04	TUBA1A,TUBA4A
Cancer	relapsed Philadelphia chromosome-negative acute lymphoblastic leukemia	9.86E-04	TUBA1A,TUBA4A
Hematological Disease	relapsed Philadelphia chromosome-negative acute lymphoblastic leukemia	9.86E-04	TUBA1A,TUBA4A
Immunological Disease	relapsed Philadelphia chromosome-negative acute lymphoblastic leukemia	9.86E-04	TUBA1A,TUBA4A
Cancer	unresectable, advanced non-small cell lung cancer	9.86E-04	TUBA1A,TUBA4A
Respiratory Disease	unresectable, advanced non-small cell lung cancer	9.86E-04	TUBA1A,TUBA4A
